# Immunogenicity of NVX-CoV2373 heterologous boost against SARS-CoV-2 variants

**DOI:** 10.1038/s41541-023-00693-z

**Published:** 2023-07-11

**Authors:** Kirsten E. Lyke, Robert L. Atmar, Clara Dominguez Islas, Christine M. Posavad, Meagan E. Deming, Angela R. Branche, Christine Johnston, Hana M. El Sahly, Srilatha Edupuganti, Mark J. Mulligan, Lisa A. Jackson, Richard E. Rupp, Christina A. Rostad, Rhea N. Coler, Martín Bäcker, Angelica C. Kottkamp, Tara M. Babu, David Dobrzynski, Judith M. Martin, Rebecca C. Brady, Robert W. Frenck, Kumaravel Rajakumar, Karen Kotloff, Nadine Rouphael, Daniel Szydlo, Rahul PaulChoudhury, Janet I. Archer, Sonja Crandon, Brian Ingersoll, Amanda Eaton, Elizabeth R. Brown, M. Juliana McElrath, Kathleen M. Neuzil, David S. Stephens, Diane J. Post, Bob C. Lin, Leonid Serebryannyy, John H. Beigel, David C. Montefiori, Paul C. Roberts, the DMID 21-0012 Study Group, Evan J. Anderson, Megan Berman, Kristen W. Cohen, Stephen De Rosa, Michelle Dickey, Jennifer Lee Dong, Madison Ellis, Ann R. Falsey, Andrew B. Fleming, Katharine Floyd, Stephanie L. Foster, Daniel Graciaa, Ahsen Kahn, Satoshi Kamidani, Wendy A. Keitel, Lilin Lai, Sasha E. Larsen, Marina Lee, Kelly Manning, Kathryn M. Moore, Vivian Mulholland, Gysella B. Muniz, Seema Nayak, Asif Noor, Mit Patel, Laura Porterfield, Angie Price, Ian Shannon, Timothy R. Shope, Amber Stanford, Mehul S. Suthar, Anna Wald, Jennifer A. Whitaker

**Affiliations:** 1 Center for Vaccine Development and Global Health, University of Maryland School of Medicine, Baltimore, MD USA; 2 Departments of Medicine and Molecular Virology & Microbiology, Baylor College of Medicine, Houston, TX USA; 3 Vaccine and Infectious Disease Division, Fred Hutchinson Cancer Center, Seattle, WA USA; 4 Department of Laboratory Medicine & Pathology, Fred Hutchinson Cancer Research Center, Seattle, WA USA; 5 Department of Medicine, Division of Infectious Diseases, University of Rochester, Rochester, NY USA; 6 Department of Medicine, University of Washington, Seattle, WA USA; 7 Department of Medicine, Emory University School of Medicine, Atlanta, USA; 8 Hope Clinic of Emory Vaccine Center, Atlanta, GA USA; 9 NYU Langone Vaccine Center and Division of Infectious Diseases and Immunology, Department of Medicine, NYU Grossman School of Medicine, New York, NY USA; 10 Kaiser Permanente Washington Health Research Institute, Seattle, WA USA; 11 Sealy Institute for Vaccine Sciences, University of Texas Medical Branch, Galveston, TX USA; 12 Department of Pediatrics and Center for Childhood Infections and Vaccines, Emory University School of Medicine and Children’s Healthcare of Atlanta, Atlanta, GA USA; 13 Seattle Children’s Research Institute, University of Washington School of Medicine, Seattle, WA USA; 14 Department of Pediatrics, University of Washington School of Medicine, Seattle, WA USA; 15 NYU Langone Hospital—Long Island Vaccine Center Research Clinic and Division of Infectious Disease, Department of Medicine, NYU Long Island School of Medicine, Mineola, NY USA; 16 Department of Pediatrics, University of Pittsburgh School of Medicine, Pittsburgh, PA USA; 17 Cincinnati Children’s Hospital Medical Center, Division of Infectious Diseases, University of Cincinnati College of Medicine, Cincinnati, OH USA; 18 Statistical Center for HIV/AIDS Research and Prevention (SCHARP), Fred Hutchinson Cancer Research Center, Seattle, WA USA; 19 FHI360, Durham, NC 27701 USA; 20 Division of Microbiology and Infectious Diseases, National Institute of Allergy and Infectious Diseases, National Institutes of Health, Bethesda, MD USA; 21 Department of Surgery, Duke University Medical Center, Durham, NC USA; 22 Vaccine Immunology Program, Vaccine Research Center, National Institute of Allergy and Infectious Diseases, National Institutes of Health, Bethesda, MD USA; 23 Duke Human Vaccine Institute, Duke University Medical Center, Durham, NC USA; 24 Emory University Laboratory (FRNT), Atlanta, GA USA

**Keywords:** Viral infection, Translational research

## Abstract

As part of a multicenter study evaluating homologous and heterologous COVID-19 booster vaccines, we assessed the magnitude, breadth, and short-term durability of binding and pseudovirus-neutralizing antibody (PsVNA) responses following a single booster dose of NVX-CoV2373 in adults primed with either Ad26.COV2.S, mRNA-1273, or BNT162b2 vaccines. NVX-CoV2373 as a heterologous booster was immunogenic and associated with no safety concerns through Day 91. Fold-rises in PsVNA titers from baseline (Day 1) to Day 29 were highest for prototypic D614G variant and lowest for more recent Omicron sub-lineages BQ.1.1 and XBB.1. Peak humoral responses against all SARS-CoV-2 variants were lower in those primed with Ad26.COV2.S than with mRNA vaccines. Prior SARS CoV-2 infection was associated with substantially higher baseline PsVNA titers, which remained elevated relative to previously uninfected participants through Day 91. These data support the use of heterologous protein-based booster vaccines as an acceptable alternative to mRNA or adenoviral-based COVID-19 booster vaccines. This trial was conducted under ClinicalTrials.gov: NCT04889209.

## Introduction

New variants of the Severe Acute Respiratory Syndrome Coronavirus 2 (SARS-CoV-2) continue to emerge and circulate, with multiple sub-lineages of the Omicron variant (B.1.1.529) remaining dominant. Vaccination is a primary strategy for the prevention of SARS-CoV-2-associated illness and complications, and boosting following completion of primary immunization regimens is recommended to enhance vaccine effectiveness^1^. NVX-CoV2373, a recombinant nanoparticle vaccine consisting of the SARS-CoV-2 spike protein and Matrix-M adjuvant, has received emergency use authorization (EUA) for use as a vaccine in the United States^2,3^. A recombinant protein vaccine offers a third and more traditional option to mRNA and adenovirus-based vaccines. However, there are few published data on NVX-CoV2373’s immunogenicity when used as a heterologous boost after a primary immunization regimen^4,5^. As part of an ongoing study evaluating homologous and heterologous COVID-19 booster vaccines^6,7^, we assessed the magnitude, breadth, and short-term durability of neutralizing activity against prototypic SARS-CoV-2 (D614G mutation) and five Omicron sub-lineages following a single booster dose of NVX-CoV2373.

## Results

### Study population

Participants (*N* = 67) who had previously received, as their primary series, Ad26.COV2.S (Janssen, 1 or 2 doses; *n* = 20), mRNA-1273 (Moderna, 2 doses; n = 16) or BNT162b2 (Pfizer, 2 doses; *n* = 31) were enrolled in the study from March 17, 2022–May 11, 2022 and received a single dose of NVX-CoV2373 comprising 5-mcg recombinant spike protein co-formulated with 50 mcg of Matrix-M adjuvant as a heterologous boost an average of 32.8–42.9 weeks (dependent upon the group) after their previous COVID-19 vaccine (Table 1, Supplementary Fig. 1). Despite a known history of prior SARS CoV-2 infection being an exclusion criterion, 15–35% of participants had serologic evidence of prior infection at enrollment, as evidenced by a positive anti-nucleocapsid (N) antibody test, and an additional 3–5 persons per study group were infected during the 90-day follow-up period which coincided with successive waves of Omicron sublineages.

**Table 1:** Baseline characteristics.

	Janssen Ad26COV-2	Moderna mRNA-1273	Pfizer BNT162b2
No. of participants	20	16	31
Sex – no. (%)			
Female	9 (45)	6 (37)	16 (52)
Male	11 (55)	10 (63)	15 (48)
Age, years			
18–55 years old – no. (%)	11 (55)	9 (56)	25 (81)
56+ years old – no. (%)	9 (45)	7 (44)	6 (19)
Mean ± SD	50.3 ± 15.9	48.4 ± 14.7	43.2 ± 11.9
Range	25–77	19–66	19–62
Race and ethnic group – no. (%)^a^			
American or Alaska Native	0 (0)	0 (0)	0 (0)
Asian	0 (0)	0 (0)	4 (13)
Black or African American	3 (15)	5 (31)	10 (32)
Multiracial	3 (15)	1 (6)	1 (3)
Native Hawaiian or other Pacific Islander	0 (0)	0 (0)	0 (0)
White	14 (70)	10 (63)	15 (49)
Other race	0 (0)	0 (0)	1 (3)
Hispanic or Latino ethnicity	2 (10)	2 (13)	3 (10)
Ethnic group not reported or unknown	1 (5)	0 (0)	0 (3)
Vaccine doses prior to Novavax boost^b^			
One dose	4	NA	NA
Two doses	16	16	31
The interval from last prior vaccine dose, weeks^c^			
Mean ± SD	32.8 ± 8.9	42.2 ± 13.3	42.9 ± 13.1
Range	12.7–54.1	14.3–59.0	18.7–76.1
Interval between first and second prior vaccine doses, weeks^d^			
Mean ± SD	22.5 ± 8.2	4.8 ± 1.3	3.2 ± 0.5
Range	15–43	4–8.7	2.7–5.3
Serology anti N-protein Ab test at baseline			
Negative	17 (85)	11 (69)	20 (65)
Positive	3 (15)	5 (31)	11 (35)

Selected baseline characteristics and retention of participants enrolled who were primed with Ad26COV-2, mRNA-2373, and BNT162b2 and boosted with NVX-CoV2373.

^a^Race and ethnic group were reported by the participant and were collected as two categories, so percentages in each category do not total 100%.

^b^NA denotes not applicable because these participants received two vaccine doses as their EUA prime immunization regimen.

^c^The interval corresponds to the time since the one-dose Ad26.COV2.S primary vaccine for those who only received one vaccine dose before enrollment in study or the time since the second Ad26.COV2.S (boost) dose or the second mRNA-1273 or BNT162b2 (prime) dose.

^d^Only for those participants who received two doses of Ad26.COV2.S (prime and boost) vaccination or two doses of mRNA-1273 or BNT162b2 (prime) vaccination.

### Safety

**Fig. 1:**
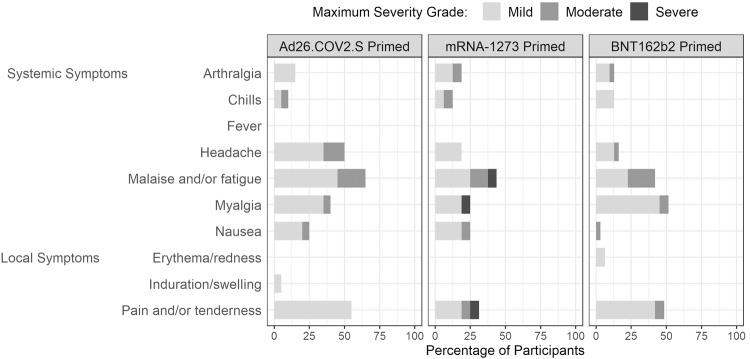
Vaccine reactogenicity. Maximum severity reported of injection site (local) and systemic solicited reactions for Ad26.COV2.S-primed, mRNA-1273-primed and BNT162b2-primed participants boosted with NVX-CoV2373. Symptoms were reported as absent, mild, moderate, or severe in the 7 days after NVX-CoV2373 vaccination.

The vaccine was well tolerated, with the most common solicited local adverse event (AE) being injection site discomfort and the most common solicited systemic AEs being malaise, myalgia and headache (Fig. 1, Supplementary Table 1). AEs were generally mild to moderate in severity. Unsolicited AEs that were deemed related to study product, including lymphadenopathy, axillary or neck pain, dizziness and night sweats, were reported by 3 (15%), 1 (6.3%), and 2 (6.5%) participants in the Ad26.COV2.S-, mRNA-1273-, and BNT162b2-primed groups, respectively (Supplementary Table 2). Most reported unsolicited AEs were mild or moderate, with three classified as severe (all elevated blood pressure not related to study product; one each per study group). One participant in the BNT162b2-primed group developed a heat stroke complicated by a seizure 103 days after vaccination that was reported as a serious AE and AE of special interest (seizure) deemed unrelated to study product.

### SARS-CoV-2 binding antibody by ECLIA

All participants had demonstrable WA-1-S-2P binding antibody before booster vaccination (Table 2). Persons who received one- or two-doses of Ad26.COV2.S as the primary series had Day 1 baseline titers against WA-1 that were 3.4–4.4-fold lower, and baseline titers against B.1.1.529 that were 6.1–6.6-fold lower than baseline titers in persons who received mRNA vaccines for the primary series. All groups had increased binding antibody following NVX-CoV2373 boost with geometric mean titers (GMT) peaking at Day 15. The geometric mean fold rises in binding antibody against WA-1 at Day 15 ranged from 2.8–5.2 and were greatest in those who received Ad26.COV2.S primary vaccination, likely due to the lower baseline values. Among persons primed with mRNA-1273 or BNT162b2, similar binding antibody responses to the NVX-CoV2373 boost were noted.

**Table 2:** SARS-CoV-2 IgG binding antibody responses IgG serum antibody titers to vaccine (S-2P-WA-1 and Omicron B.1.1.529 strains) and reported as areas under the curve (AUC) via a 10-plex ECLIA assay.

Group	15	16	17
Primary EUA immunization Vaccine	Janssen	Moderna	Pfizer/BioNTech
	Ad26.COV2-S	mRNA-1273	BNT162b2
	5 × 10^10^ vp	100-mcg	30-mcg
Booster	Novavax (NVX-CoV2373) 5-mcg plus 50-mcg Matrix M		
Vaccine (S-2P-WA-1) Strain			
Day 1 GMT^a^ (95% CI)^b^	4123.0	18,310.7	14,083.8
	(2352.67–7225.51)	(11,647.69-28,785.21)	(9270.68-21,395.63)
	*N* = 20	*N* = 16	*N* = 31
Day 15 GMT (95% CI)	22,969.7	50,130.9	44,295.7
	(15,905.3–33,171.7)	(44,100.3–56,986.2)	(39,894.3–49,182.7)
	*N* = 19	*N* = 15	*N* = 29
Day 29 GMT (95% CI)	20,950.5	46,053.9	42,857.8
	(13,923.5–31,524.0)	(38,716.7–54,781.5)	(38,031.1–48,297.0)
	*N* = 18	*N* = 14	*N* = 29
Day 91 GMT (95% CI)	15,093.62	44,220.6	38,321.0
	(9651.7–23,603.8)	(35,889.0–54,486.3)	(30,415.6–48,281.0)
	*N* = 19	*N* = 13	*N* = 25
Percentage with twofold rise at Day 15 (95% CI)	84.2%	60.0%	62.1%
	(60.4–96.6%)	(32.3–83.7%)	(42.3–79.3%)
Geometric mean fold rise at Day 15 (95% CI)	5.2	2.8	3.2
	(3.3–8.3)	(1.8–4.3)	(2.2–4.8)
Omicron B.1.1.529			
Day 1 GMT (95% CI)	535.5	3511.1	3269.6
	(277.0–1035.0)	(1750.2–7043.9)	(1828.8–5845.6)
	*N* = 20	*N* = 16	*N* = 31
Day 15 GMT (95% CI) (*n*)	5968.6	21,524.8	16,656.8
	(3459.4–10,297.6)	(15,803.9–29,316.7)	(13,114.4–21,156.1)
	*N* = 19	*N* = 15	*N* = 29
Day 29 GMT (95% CI) (*n*)	5164.3	17,202.8	15,484.3
	(2788.6-9564.0)	(1129.70–26,210.7)	(11,969.5–20,031.4)
	*N* = 18	*N* = 14	*N* = 29
Day 91 GMT (95% CI) (*n*)	3184.4	14,911.9	13,089.4
	(1790.0-5665.1)	(8938.6–24,877.0)	(8831.1–19,401.0)
	*N* = 19	*N* = 13	*N* = 25
GM fold decrease relative to WA-1 at Day 15 (95% CI)	4.7	3.0	2.9
	(3.8–5.9)	(2.2–4.1)	(2.4–3.5)
Day 15 geometric mean fold rise (95% CI)	11.8	6.0	5.4
	(6.6–21.1)	(3.1–11.4)	(3.3–8.7)

Results are reported by primary immunization vaccine and timepoint relative to administration of the booster vaccine.

^a^*GMT* Geometric mean titers.

^b^*CI* Confidence intervals. The confidence intervals have not been adjusted for multiplicity, so the intervals should not be used to infer definitive treatment effects for secondary outcomes.

At baseline (Day 1), the binding antibody levels to the Omicron variant were 77–87% lower than binding Ab levels to WA-1 using the same 10-plex assay. Following boost, 94.7% (Ad26.COV2.S-primed), 73.3% (mRNA-1273-primed) and 65.5% (BNT162b2-primed) of participants had at least a twofold rise in binding antibody to Omicron and the levels were 53–75% lower at peak, Day 15, compared to the WA-1 strain. Serologic responses to WA-1 and Beta S-2P by 4-plex ECLIA and for WA-1, Beta, Delta and Omicron S-2P proteins on the 10-plex23 ECLIA are reported in the Supplementary Appendix (Supplementary Tables 3–14). In general, sera from the older age groups (≥56 years) had reduced binding antibody levels (Supplementary Appendix).

### SARS-CoV-2 neutralizing antibody responses

**Fig. 2:**
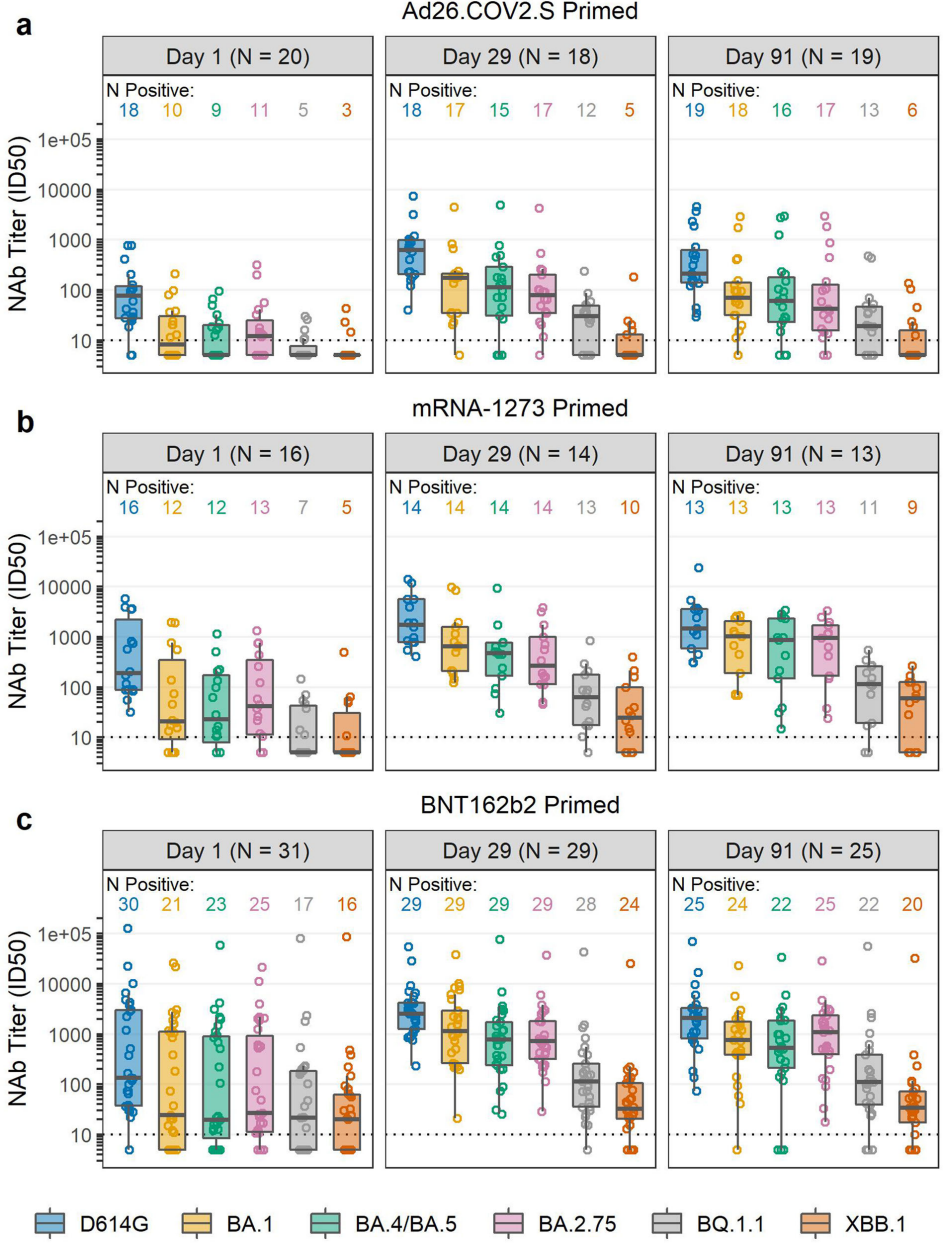
Pseudovirus neutralization antibodies expressed as 50% inhibitory dilution (ID50) to the D614G variant and Omicron sub-lineages at Day 1 (pre-booster) and Days 29 and 91 post-NVX-CoV2373 booster. The boxplot panels represent (**a**) Ad26.COV2.S-primed, (**b**) mRNA-1273-primed; and (**c**) BNT162b2-primed participants boosted with NVX-CoV2373. The number of participants with serum samples collected and assayed at each visit are shown at the top of each panel. The number of positive samples against each of the variant strains are shown above each box plot. Box plots represent median (horizontal line within the box) and 25th and 75th percentiles (lower and upper borders of the box), with the whiskers drawn to the value nearest to, but within, 1.5× interquartile range above and below the borders of the box and individual results depicted in open circles. The lower level of detection is 10 and technical duplicates were performed.

Pseudovirus neutralizing antibodies (PsVNA) were assessed against the prototypic D614G variant and five Omicron sub-lineages (BA.1, BA.4/BA.5, BA.2.75, BQ.1.1 and XBB.1) in all participants. At baseline, 90% of Ad26.COV2.S-primed and 97–100% of mRNA-primed recipients had detectable pre-booster PsVNA to the prototypic D614G variant, with geometric mean ID50 titers (GMTs) ranging from 64.5–355 across primed groups (Table 3, Supplementary Tables 15, 16). Baseline positive response rates and PsVNA GMTs were lower for all Omicron and variant sub-lineages tested, and especially for BQ.1.1 and XBB.1 (Supplementary Tables 17–28). Following the NVX-CoV1273 booster, 100% of participants in all three groups had detectable PsVNA against D614G, which remained detectable in all participants through Day 91 (Table 3). PsVNA GMTs to D614G and BA.1 peaked at Day 15; however, this time point was not tested for the Omicron sub-lineages; therefore Day 29 results are reported as the estimated peak response (Table 3, Fig. 2). PsVNA against BA.1 and BA.4/BA.5 exhibited the highest geometric mean fold rise at Day 29 in all three groups (7.8–17.7-fold GMT rise), although GMTs were 2.8–5.3 times lower than those to D614G. The geometric mean fold rise in PsVNA at Day 29 was lowest for the more recent Omicron BQ.1.1 and XBB.1 sub-lineages (1.2–5.1-fold GMT rise) in all three groups, with GMTs that were 21.2–72.5 times lower than those to D614G. GMTs against all variants declined <2-fold from Day 29 to Day 91 in all three groups.

**Table 3:** SARS-CoV-2 neutralizing antibody responses (ID50) following Novavax NVX-CoV2373 boost geometric mean ID50 neutralizing antibody titers to pseudovirus D614G and to B.1.1.529 sublineages.

SARS-CoV-2 neutralizing antibody responses (ID50)							
Variant	N	D614G	BA.1	BA.4/5	BA.2.75	BQ1.1	XBB.1
Prime- Janssen Ad26.COV2.S 5 × 10^10^ vp (1 or 2 doses)							
Day 1 GMT^a^ (95% CI)^)b^	20	64.5	13.4	11.03	13.8	6.8	6.3
		(33.7–123.6)	(7.6–23.4)	(6.9–17.7)	(7.6–24.8)	(5.2–8.9)	(4.8–8.4)
Day 15 GMT (95% CI)	19	619.8	126.8	ND	ND	ND	ND
		(342.7–1121.0)	(61.8–260.2)				
Day 29 GMT (95% CI)	18	499.3	114.8	93.8	80.1	21.7	8.1
		(269.7–924.3)	(51.7–254.8)	(37.7–233.1)	(37.5–171.2)	(12.0–39.2)	(5.0–13.0)
Day 91 GMT (95% CI)	19	305.5	82.9	69.7	64.7	22.7	9.6
		(149.4–624.5)	(38.0–180.8)	(27.6–176.4)	(26.5–158.4)	(11.5–44.5)	(5.6–16.3)
GM fold decrease relative to D614G at Day 29 (95% CI)		–	4.4	5.3	6.2	23.0	61.7
			(3.3–5.8)	(3.5–8.1)	(4.1–9.4)	(15.4–34.3)	(37.4–101.9)
Geometric mean fold rise at Day 29 (95% CI)		6.4	8.6	7.8	5.5	3.1	1.2
		(4.2–9.9)	(5.3–13.9)	(3.8–15.8)	(3.2–9.5)	(1.8–5.2)	(0.9–1.7)
Prime- Moderna mRNA-1273 100-mcg							
Day 1 GMT (95% CI)	16	347.4	47.7	36.9	56.0	12.4	11.0
		(138.0–874.6)	(15.3–148.3)	(14.2–96.2)	(20.2–155.6)	(6.5–23.6)	(5.2–23.1)
Day 15 GMT (95% CI)	15	2431.3	749.5	ND	ND	ND	ND
		(1407.8–4199.0)	(323.2–1738.0)				
Day 29 GMT (95% CI)	14	1978.3	704.3	400.7	351.3	56.5	27.3
		(1024.8–3819.0)	(314.0–1579.8)	(177.5–904.4)	(153.1–806.2)	(24.8–128.4)	(11.5–64.5)
Day 91 GMT (95% CI)	14	1661.4	589.3	415.5	522.7	76.2	37.0
		(776.8–3553.2)	(248.6–1396.7)	(135.4–1275.3)	(201.0–1359.1)	(29.3–198.0)	(15.0–91.4)
GM fold decrease relative to D614G at Day 29 (95% CI)		–	2.8	4.9	5.6	35.0	72.5
			(1.8–4.4)	(3.0–8.2)	(3.5–9.0)	(18.0–68.0)	(38.5–136.4)
Geometric mean fold rise at Day 29 (95% CI)		6.4	17.7	11.3	6.3	5.1	3.1
		(2.4–17.6)	(7.2–43.4)	(4.7–27.5)	(2.9–13.9)	(2.8–9.4)	(1.6–5.8)
Prime- Pfizer/BioNTech BNT162b2 30-mcg							
Day 1 GMT (95% CI)	31	355.3	87.5	75.5	96.5	38.4	24.0
		(142.0–889.2)	(30.8–248.8)	(28.1–202.9)	(36.8–253.4)	(15.8–93.4)	(11.0–52.0)
Day 15 GMT (95% CI)	29	2839.9	816.0	ND	ND	ND	ND
		(1786.3–4515.1)	(472.8–1408.6)				
Day 29 GMT (95% CI)	29	2681.8	1181.1	657.4	766.3	126.2	40.7
		(1762.6–4080.3)	(639.0–2183.1)	(349.4–1236.6)	(451.4–1300.7)	(64.4–247.5)	(21.3–77.6)
Day 91 GMT (95% CI)	25	1741.7	627.3	449.5	731.5	128.2	40.3
		(949.2–3195.8)	(307.7–1278.9)	(187.9–1075.7)	(370.4–1444.6)	(53.8–305.8)	(18.8–86.2)
GM fold decrease relative to D614G at Day 29 (95% CI)		–	2.3	4.1	3.5	21.2	65.9
			(1.6–3.2)	(2.9–5.9)	(2.6–4.7)	(14.3–31.5)	(44.6–97.3)
Geometric mean fold rise at Day 29 (95% CI)		7.8	14.1	9.2	8.4	3.4	1.8
		(3.9–15.9)	(6.6–30.1)	(4.9–17.2)	(4.1–17.1)	(1.8–6.5)	(1.0–3.1)

Results are reported by primary immunization EUA vaccine and timepoint.

^a^*GMT* Geometric mean titers.

^b^*CI* Confidence intervals. The confidence intervals have not been adjusted for multiplicity, so the intervals should not be used to infer definitive treatment effects for secondary outcomes.

### SARS CoV-2 infection determined by nucleocapsid-specific antibody responses

**Fig. 3:**
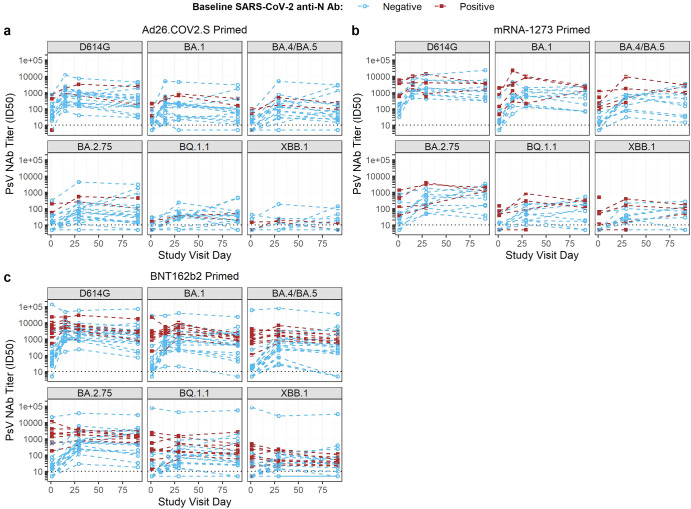
Pseudovirus neutralization antibodies (ID50) to the D614G variant and Omicron sub-lineages at Day 1 (pre-booster) and Days 29 and 91 post-NVX-CoV2373 booster in individuals, stratified by prior exposure to SARS-CoV-2 as detected by N protein. The spaghetti plot depicts (**a**) Ad26.COV2.S-primed, (**b**) mRNA-1273-primed; and (**c**) BNT162b2-primed participants boosted with NVX-CoV2373. Each line represents the PsVNA at Days 1, 29 and 91 in individuals with detectable anti-N protein antibody by ELISA prior to NVX-CoV2373 boost (red) or without detectable N protein (blue). The dotted gray line depicts the lower level of detection.

Nucleocapsid (N)-specific antibody was detected in 28% of participants at the time of enrollment [Ad26.COV2.S (3 of 20); mRNA-1273 (5 of 15); and BNT162b2 (11 of 31)]. Twelve additional participants had intercurrent infections as evidenced by development of SARS CoV-2 N-specific antibody and/or a confirmed SARS CoV-2 virus detection test (PCR or antigen) through Day 91 post-boost: Ad26.COV2.S [2 antibody only, 3 virus (identified on Day 91, two subsequently developing detectable N antibody)], mRNA-1273 (1 antibody only, 2 both antibody and virus), BNT162b2 (2 antibody only, 2 both antibody and virus) (Supplementary Fig. 3). The effect of pre-boost exposure to Covid-19 increased the detectable post-boost PsVNA ID50 titers to D614G, and B.1.1.529 (Omicron) sublineages BA.1, BA.4/BA.5, BA.2.75, BQ1.1, and XBB.1 (Fig. 3). All but one participant who received an mRNA primary series (mRNA-1273 or BNT162b2) and were N-antibody positive at baseline had detectable baseline responses to all Omicron sublineages (one mRNA-1273 participant had BQ.1.1 and XBB.1 GMTs below the lower limit of detection (LLD)). Participants who had received Ad26.COV2.S as a primary (1 or 2 doses) and were N antibody-positive at baseline developed PsVNA titers to BA.1, BA.4/BA.5 and BA.2.75 but had PsVNA titers at or near the LLD for BQ.1.1 and XBB.1.

### SARS CoV-2 -specific T cell responses

Spike-specific Th1 cytokine-expressing CD4+ T cells (interferon-γ [IFN-γ], interleukin-2 [IL-2], or both) were detected at baseline in 50%–88% of participants (Supplementary Fig. 4, Supplementary Tables 29, 30) and were present in all but two participants (BNT162b2-primed) at Day 15. Spike-specific Th2 cytokine expressing CD4+ T cells (interleukin-4, interleukin-5 or interleukin-13) were largely negative at baseline, and although there was an increase at Day 15 in many participants across all groups, the magnitude was too low to be determined as positive in most of these (maximum response rate of 20%, with a tendency for more positive responses in the younger age group (Supplementary Tables 4, 31, 32). The numbers of spike-specific CD8+ cells expressing IFN-γ and/or IL-2 were higher at baseline in the Ad26CoV2.S-primed participants (55%) than in those primed with a mRNA vaccine (13–28%), with little difference in response by Day 15 between groups (Supplementary Tables 4, 33, 34).

## Discussion

NVX-CoV2373 was immunogenic and well-tolerated with no identified safety concerns when used as a heterologous COVID-19 booster in persons who previously had received Ad26.COV2.S, mRNA-1273 or BNT162b2 vaccines for primary immunization. The protein-based NVX-CoV2373 offers a more traditional vaccine option as a booster dose, but little is known of its ability to elicit sufficient neutralizing responses against the emerging Omicron sub-lineages. As part of an ongoing multicenter study evaluating homologous and heterologous booster dose immunological responses, we were able to examine antibody responses in participants who were primed with adenoviral-vectored or mRNA-based vaccines. We found a modest booster response (6.4–7.8-fold rise in GMT at Day 29) to ancestral strain D614G, with a more robust response to B.1.1.529 (Omicron) BA.1 (8.6–17.7-fold GMT rise) and BA.4/BA.5 (7.8–11.3-fold GMT rise), and lowest rise to more recent circulating sub-lineages such as BQ1.1 (3.1–5.1-fold GMT rise) and XBB.1 (1.2–3.1-fold GMT rise).

NVX-CoV2373, while authorized as a two-dose primary series, has a potentially practical niche in high resource countries as a first booster dose at least 6 months following a primary series. It also serves an important role internationally as a prime vaccine or an alternative to individuals with safety concerns related to mRNA or adenovirus-vectored vaccines. The data within expands upon the use of NVX-CoV2373 as a heterologous booster. It is currently FDA-authorized for use in persons 18 years of age or greater when an mRNA bivalent booster vaccine is not accessible or for those who would choose to avoid a booster in the absence of a protein booster option^3^. In general, the safety profile suggested that there were less local and systemic adverse events with the protein-based NVX-CoV2373, as compared to booster dosing with mRNA or adenoviral-vectored vaccines. Similar to our previous findings, a trend towards slightly more severe events was noted in mRNA-1273-primed participants but the trial was not designed to compare between groups^6^. The sample size limits the detection of rare events that may occur on a population level.

As part of a study examining heterologous and homologous first booster doses, we examined NVX-CoV2373 following mRNA or adenovirus-prime dosing. Previously, in a study performed by the manufacturer, homologous booster doses of NVX-CoV2373 resulted in ~34- and 96-fold increases, respectively, in IgG titers and microneutralization antibodies against ancestral strains and a 20-fold increase in IgG titers against Omicron (BA.1)^8^. In another study, two doses of Novavax as a priming vaccine followed by a Pfizer/BioNTech booster vaccine resulted in an ~100-fold increase in anti-S IgG against SARS-CoV-2^9^. Similar to previous findings, our study confirms that the vaccine prime heavily influences the titers following boost as PsVNA titers in mRNA-primed participants were much higher than those in those detected in Ad26.COV2.S -primed individuals^6^. Notably, despite a difference in total mRNA administered between the 100-μg mRNA-1273 and 30-μg BNT162b prime vaccines, the pre-boost PsVNA titers were nearly identical and little difference was noted in peak titers following NVX-CoV2373 booster dosing. This contrasts with the low pre-boost and peak PsVNA titers of the Ad26.COV2.S-primed group despite a similar geometric mean fold rise (6.4-fold) in titers. In addition, while all three primed groups had evidence of spike-specific Th1 cytokine expression from CD4 + T cells at baseline and responded modestly to a booster dose of NVX-CoV2373, only the Ad26.COV2.S- primed participants (11/20) had a high proportion of spike-specific Th1 cytokine expression from CD8+ T cells at baseline with little to no boosting effect noted in any primed group.

Participants received the NVX-CoV2373 booster dose in March 2022 following the BA.1 Omicron wave of infections. Up to one third (15–35%) of participants had evidence of baseline anti-N-protein antibody despite participant confirmation that they had not been diagnosed with a SARS-CoV-2 infection prior to boost. The BNT162b2-primed group was heavily skewed towards individuals <55 years of age (81%), in contrast to the other two prime groups, and the interval between prime and boost was the longest, which may account for the high prevalence of N protein antibodies (35%). The baseline PsVNA titers were markedly increased in N antibody-positive as compared to N antibody-negative participants. In the case of the Pfizer/BioNTech primed participants, a 36-fold differential was noted to ancestral strain D614G at baseline [PsVNA ID50 GMTs 99 vs 3620 for N antibody-negative vs N antibody-positive, respectively]. The baseline differential was even more extreme for Omicron sub-lineages. The effect of booster dosing in the setting of pre-existing infection to SARS-CoV-2 was an increase in the detectable endpoint PsVNA titers as compared to previously uninfected participants, but a reduced geometric mean fold rise in N antibody-positive participants compared to N antibody-negative participants resulting in a differential PsVNA ID50 titer at Day 91 that narrowed to a ~1.8–7.25-fold difference stratified by baseline N protein seroreactivity. An additional 12 participants (18%) experienced a documented SARS-CoV-2 infection by Day 91. This suggests limited benefit following additional booster doses for individuals with pre-existing hybrid immunity.

Limitations of this study included the individual variability in boosting intervals and high exposure rates to SARS-CoV-2 both before and during trial follow-up. In addition, at the time of study recruitment, much of the population willing to receive Covid-19 booster vaccinations had already received an mRNA or adenovirus-based booster vaccine (one or two doses), complicating enrollment. Thus, while our sample size goal was 60 per vaccine prime group, stratified equally between those aged less than or greater than 56 years (*n* = 180), recruitment was limited to a small sample size of 67 individuals across the three vaccine prime groups. We did not enroll a NVX-CoV2373-primed group for a homologous comparison. We also did not directly compare NVX-CoV2373 booster responses to mRNA boosts, although substantial neutralization escape by BQ.1.1 and XBB.1 sublineages following monovalent and bivalent mRNA booster vaccines have also been described^10^. Finally, we do not report B or T cell responses durability (studies planned), which may add additional nuanced understanding of the immunological responses to vaccination and in particular cross-strain protective efficacy. T cell responses, notably induced by COVID-19 boosters, may provide protection against severe disease and death^11,12^.

These data support the use of protein-based booster vaccines as a heterologous booster option and as an alternative to mRNA or adenoviral-based COVID-19 vaccines. NVX-CoV2373 modestly increased humoral immunity to Omicron sub-lineages BA.1, BA.4/BA.5, and BA.2.75 in those primed with an mRNA vaccine, but reduced peak humoral responses were noted in those primed with Ad26.COV2.S, and a low-level response was noted to newer circulating Omicron sub-lineages such as BQ1.1 and XBB.1 in all combinations. This may limit clinical effectiveness given recent circulating strains. The presence of anti-N-protein antibodies, indicative of a preceding SARS-CoV-2 infection, contributed to dramatically higher baseline PsVNA GMT, which remained elevated relative to the unexposed participants through Day 91, providing real-world insight to vaccine responses considering the numerous and sequential Omicron strain circulation. Additional studies are needed to determine whether improved variant-specific immune responses can be achieved with updated protein-based vaccines.

## Methods

### Study design

This phase 1/2 study (NCT04889209) is an open-label, adaptive design evaluation of different booster vaccines among persons who previously completed one of three COVID-19 vaccine priming regimens under EUA at least 12 weeks prior to enrollment: one (or two) dose(s) of Ad26.COV2.S (5 × 10^10^ viral particles), two doses of 100-mcg mRNA-1273 at least 4 weeks apart, or two doses of 30-mcg BNT162b2 at least 3 weeks apart. This was stage 6 of the study and evaluated NVX-COV2373 as a booster vaccine. All but 4 of the Ad26.COV2.S primed group had received a second dose of the same vaccine based upon CDC recommendations^13^ at the time study enrollment was conducted (March 17-May 11, 2022). Planned enrollment was up to 60 per priming vaccine group.

### Study participants

Study participants were males and females who were 18 years of age or older and were in good health. Females agreed to practice adequate contraception from 28 days before enrollment to 3 months afterwards, had a negative pregnancy test and were not breastfeeding. Exclusion criteria included acute illness at the time of vaccine dosing, known history of SARS CoV-2 infection, prior receipt of investigational coronavirus vaccines or SARS-CoV2 monoclonal antibody, receipt of other investigational products, plans to receive a vaccine within 28 days of enrollment, current or recent participation in an interventional trial, significant bleeding disorder, immunocompromising condition or immunosuppressive treatment, and receipt of blood products within 90 days.

### Human Participant Research

Research was conducted in accordance with the Declaration of Helsinki. The trial (NIH/NIAID/DMID – 21-0012; Pro00053376) was reviewed and approved by a central institutional review board, Advarra (Columbia, MD), and overseen by an independent safety monitoring committee. All participants provided written informed consent before undergoing any study-related activities.

### Study vaccine

NVX-CoV2373 is a vaccine containing 5-mcg of the recombinant spike protein from the prototype Wuhan strain co-formulated with 50-mcg of Matrix-M adjuvant in a 0.5 mL volume. Vaccine (0.5 mL) was administered intramuscularly into the deltoid.

### Study outcomes

The primary objectives of the study are to evaluate the safety and reactogenicity of delayed heterologous booster vaccine doses after a primary series of an EUA or approved vaccine, and to evaluate the breadth of the humoral immune responses following administration of the booster vaccines. The latter is assessed by evaluation of the response rate and magnitude of SARS COV-2-specific antibody binding and neutralization titer in serum samples collected at serial time points after vaccination.

### Safety assessment

Solicited injection site and systemic adverse reactions, including oral temperature, were assessed daily by study participants for 7 days after vaccination and were recorded on a memory aid. This information was reviewed during a phone interview on study Day 8 and again on Day 15. Unsolicited adverse events were documented for 28 days following vaccination and relationship to study vaccine determined. Serious adverse events, new onset chronic medical conditions, adverse events of special interest, and medically attended adverse events (related to study product) were also recorded for 12 months after vaccination. We report these latter data through 3 months. Respiratory illnesses during the study were assessed for SARS CoV-2 infection using molecular assays available at each clinical site.

### Nasal or nasopharyngeal swabs for PCR

Participants with symptoms compatible with COVID-19 presented for unscheduled Illness Visits and a nasal/nasopharyngeal swab was obtained to assess for the presence of SARS-CoV-2 virus. In addition, we included participants self-reported positive COVID-19 results obtained outside the study to include antigen and PCR assays.

### Pseudovirus neutralization

Pseudotyped lentiviruses expressing full length SARS-CoV-2 spike proteins on their surface and containing a firefly luciferase reporter gene were used in neutralization assays. Sera were assessed for their ability to prevent infection by the pseudotyped virus of 293 T/ACE2 cells as determined by the reduction in luciferase reporter activity, and the dilution leading to a 50% reduction in relative light units (RLUs) after subtraction of background RLUs determined the ID50^7,14^. Pseudotyped lentiviruses were produced and characterized for the following variants: D614G, beta (B.1.351), and omicron sublineages BA.1, BA.4/BA.5, BA.2.75, BQ.1.1, and XBB.1^6,7,15^.

### Binding antibody by ECLIA

As previously described, serum IgG binding antibody levels against the spike (S) protein with proline modification (S-2P) were evaluated by means of the 384-well Meso Scale Discovery (MSD; Rockville, MD) Electrochemiluminescence immunoassay, version 2 (4-plex ECLIA V.2) and the 10-plex ECLIA, Panel 23, for emerging SARS-CoV-2 variant spike proteins^6,16^. Plates are blocked and incubated with dilutions of serum. Bound antibody is detected with Sulfo-tag labeled anti-IgG antibody, and signal is detected after washing and application of a read solution containing an electrochemiluminescence substrate using an MSD Sector instrument. The 4-Plex readouts are expressed as arbitrary unit per mL (AU/mL) assigned by MSD reference standard and 10-plex readouts are expressed as Area Under the Curve (AUC).

### Nucleocapsid antibody ELISA

The presence of anti-nucleocapsid antibody was detected using a commercially available electrochemiluminescence immunoassay (ELISA; ELECSYS, Roche, Indianapolis, IN) with sera samples collected on Days 1 (baseline) and D29 and D91 post-booster vaccination. PsVNA ID50 titers to variants D614G and B.1.1.529 (Omicron) BA.1, BA.4/BA.5, BA.2.75, BQ1.1, and XBB.1 were stratified by anti-N antibody presence at Day 1 (baseline), age and group.

### Sars-CoV-2-specific T cell flow cytometry

The presence and frequency of SARS CoV-2 spike-specific CD4+ and CD8 + T cells were determined following ex vivo stimulation of cryopreserved peripheral blood mononuclear cells using intracellular cytokine staining as described previously^6,17^.

### Statistical analyses

The primary objectives of the study for participants enrolled in these groups is to provide estimates of the safety and immunogenicity of the Novavax boost. For practical reasons, a sample size of 60 participants per group (30 per age stratum) was targeted, consistent with phase 1–2 studies. Reported summaries are descriptive and no tests of hypothesis were planned for comparisons between groups.

All enrolled participants received the intended boost and are included in the analyses of safety and immunogenicity endpoints, which include information collected up to the Day 91 visit. Safety is evaluated as number and proportion of participants reporting solicited and unsolicited AEs. Immunogenicity endpoints, presented as unadjusted Geometric Mean (GM) titers with 95% confidence intervals, include all participants with collected and assayed serum or PBMC samples at each visit. To aid in the interpretation of the results, available evidence of SARS-CoV-2 infections prior to enrollment (presence of anti-nucleocapsid antibody) or of interval infections up to Day 91 (SARS-CoV-2 tests, presence of anti-nucleocapsid antibody) is shown in some of the graphical displays of the data.

### Reporting summary

Further information on research design is available in the Nature Research Reporting Summary linked to this article.

## Supplementary information

Supplementary Information

REPORTING SUMMARY

## Data Availability

All data generated or analyzed during this study are included in this published article and its supplementary information files.
