# Supplementary material for: Immunogenicity of NVX-CoV2373 heterologous boost against SARS-CoV-2 variants
Source: NPJ Vaccines. 2023 Jul 11;8:98. doi: 10.1038/s41541-023-00693-z (PMC10336079; doi:10.1038/s41541-023-00693-z)
Supplement: Supplementary file 1 — Supplementary Information [file 41541_2023_693_MOESM1_ESM.pdf]

Supplementary Information

This Supplementary Information is submitted by the authors to provide additional information about their work.

Supplement to: Lyke KE*, Atmar RL*, et al. Immunogenicity of NVX-CoV2373 Heterologous Boost Against SARS-CoV-2 Variants

*Contributed equally

Supplementary Information to Manuscript Entitled

Immunogenicity of NVX-CoV2373 Heterologous Boost Against SARS-CoV-2 Variants

Table of Contents

Mix and Match Study Team Members 4

Supplementary Figures

Supplementary Figure 1. CONSORT DIAGRAM 8
Supplementary Figure 2. Pseudovirion Neutralization Antibody (ID50) to VoC by Primed Group 9
Supplementary Figure 3. Pseudovirion Neutralization Antibody (ID50) to VoC by Group, Subject and Nab 10
Supplementary Figure 4. T cell Cytokine Expression by Group 11

Supplementary Tables

Supplementary Table 1: Solicited Reactogenicity by Study Group 12
Supplementary Table 2: Unsolicted Adverse Events to Day 91 by Group and Severity 13
Supplementary Table 3: IgG Serum Binding Antibody Response to S-2P-WA-1 by 4-plex ECLIA by Group 14
Supplementary Table 4: IgG Serum Binding Antibody Response to S-2P-WA-1 by 4-plex ECLIA by Group & Age 16
Supplementary Table 5: IgG Serum Binding Antibody Response to S-2P-B.351 by 4-plex ECLIA by Group 18
Supplementary Table 6: IgG Serum Binding Antibody Response to S-2P-B.351 by 4-plex ECLIA by Group & Age 20
Supplementary Table 7: IgG Serum Binding Antibody Response to S-2P-WA-1 by 10-plex23 ECLIA by Group 22
Supplementary Table 8: IgG Serum Binding Antibody Response to S-2P-WA-1 by 10-plex23 ECLIA by Group & Age 24
Supplementary Table 9: IgG Serum Binding Antibody Response to S-2P-B.351 by 10-plex23 ECLIA by Group 26
Supplementary Table 10: IgG Serum Binding Antibody Response to S-2P-B.351 by 10-plex23 ECLIA by Group & Age 28
Supplementary Table 11: IgG Serum Binding Antibody Response to S-2P-B.1.617 by 10-plex23 ECLIA by Group 30
Supplementary Table 12: IgG Serum Binding Antibody Response to S-2P-B.1.617 by 10-plex23 ECLIA by Group&Age… .32
Supplementary Table 13: IgG Serum Binding Antibody Response to S-2P-B.1.1.529 by 10-plex23 ECLIA by Group 34
Supplementary Table 14: IgG Serum Binding Antibody Response to S-2P-B.1.1.529 by 10-plex23 ECLIA by Group & Age 36
Supplementary Table 15: Pseudovirion Neutralization Antibody (ID50) to D614G 38
Supplementary Table 16: Pseudovirion Neutralization Antibody (ID50) to D614G by Age 40
Supplementary Table 17: Pseudovirion Neutralization Antibody (ID50) to B.1.351 42
Supplementary Table 18: Pseudovirion Neutralization Antibody (ID50) to B.1.351 by Age 44
Supplementary Table 19: Pseudovirion Neutralization Antibody (ID50) to B.1.1.529 BA.1 46
Supplementary Table 20: Pseudovirion Neutralization Antibody (ID50) to B.1.1.529 BA.1 by Age 48
Supplementary Table 21: Pseudovirion Neutralization Antibody (ID50) to B.1.1.529 BA.4/BA.5 50
Supplementary Table 22: Pseudovirion Neutralization Antibody (ID50) to B.1.1.529 BA.4/BA.5 by Age 52
Supplementary Table 23: Pseudovirion Neutralization Antibody (ID50) to B.1.1.529 BA.2.75 54
Supplementary Table 24: Pseudovirion Neutralization Antibody (ID50) to B.1.1.529 BA.2.75 by Age 56
Supplementary Table 25: Pseudovirion Neutralization Antibody (ID50) to B.1.1.529 BQ.1.1 58
Supplementary Table 26: Pseudovirion Neutralization Antibody (ID50) to B.1.1.529 BQ.1.1 by Age 60

Supplementary Table 27: Pseudovirion Neutralization Antibody (ID50) to B.1.1.529 XBB.1................................62
Supplementary Table 28: Pseudovirion Neutralization Antibody (ID50) to B.1.1.529 XBB.1 by Age..........................64
Supplementary Table 29: CD4 T cell IFNγ/IL-2 Cytokine Expression to Spike Peptide Pool..................................66
Supplementary Table 30: CD4 T cell IFNg/IL-2 Cytokine Expression to Spike Peptide Pool by Age.......................67
Supplementary Table 31: CD4 T cell IL4 or IL5/IL13 and CD154 Cytokine Expression to Spike Peptide Pool........68
Supplementary Table 32: CD4 T cell IL4 or IL5/IL13 and CD154 Cytokine Expression to Spike Peptide Pool by Age...69
Supplementary Table 33: CD8 T cell IFNg/IL-2 Cytokine Expression to Spike Peptide Pool......................................70
Supplementary Table 34: CD8 T cell IFNg/IL-2 Cytokine Expression to Spike Peptide Pool by Age.......................71

Mix and Match Study Team Members

University of Maryland, Institute of Human Virology and Center for Vaccine Development and Global Health, Baltimore, MD, VTEU

Kirsten E. Lyke, M.D., Meagan E. Deming, M.D., Ph.D., Karen Kotloff, M.D., Angie Price, D.N.P., M.S.N., C.R.N.P., Joel Chua, M.D., Myounghee Lee, Pharm.D., Ph.D., Lisa Anderson, R.N., B.S., Amy Nelson, R.N., M.S., Salma Sharaf, Young Chae Jessica Yoo, Lisa Langer, Pharm.D., Alyson Kwon, M.S., CCRC, ACRP-PM., Sophie Harper, M.Sci., Suemoal Mathews, M.S.,M.Sci., CPhT., Paula Bernal, M.Sci, Ph.D., Eric Goldstein, Jeffrey Floyd, Marcelo Sztein, M.D., Ming Bell, M.D., Leslie Howe, Erika Stiles, Andrew Chi, Pharm.D., Phuong Tran Nguyen, Pharm.D., Yogitha Pazhani, Pharm.D., Christine Aggabao, Pharm.D., Jeannie Murray.

Baylor College of Medicine (BCM), Houston TX, Vaccine Training and Evaluation Unit (VTEU)

Robert L. Atmar, M.D.; Hana El Sahly, M.D.; Jennifer A. Whitaker, M.D.; Wendy A. Keitel, M.D.; Mary Healy, M.D.; Christine Akamine, M.D.; Pedro A. Piedra, M.D.; Chanei Henry, A.A.S.; Brandie Phillips, R.N.: Chianti Wade-Bowers, B.S.N, M.S.N.; Connie Rangel, R.N.; Logan Lee; Julia Guardado; Angelica Diaz, F.N.P.; Lisreina Toro; Tina Sierra; Janet Brown, J.D., R.Ph. : Cathy Faw, R.Ph.; Yvette Rugeley; Yolanda Rayford; Kayla Burrell; Jesus Banay; Tykel Eddy; Marinna Matta; Kathy Bosworth, B.A.

Kaiser Permanente Washington Health Research Institute, Seattle, WA, VTEU

Lisa A. Jackson, MD, MPH, Lee Barr, R.N., Cassandra Bryant, B.S., Roger Calvert, PA-C, Barbara Carste, M.P.H., Joe Choe, B.S., Maya Dunstan, M.S., R.N., Jana ffitch, L.P.N., Colin Fields, M.D., Lynn Gross, PA-C, Erika Kiniry, M.P.H., Bonnie Lam, PharmD, De Vona Lang, Rebecca Lau, PharmD, Stella Lee, B.A., Paula Lins, PA-C, M.P.H., Amy Mohelnitzky, PA-C, Marilyn Nguyen, B.S., Matthew Nguyen, M.P.H., Stephanie Pimienta, B.S., Melissa Resendiz Rivas, B.A., Melissa Boothe Scheer, PA-C, Janice Suyehira, M.D, and Stacie Wellwood, L.P.N.

Seattle Children's Research Institute, Seattle, WA subsidiary to Kaiser Permanente VTEU

Rhea N. Coler, M.Sc., Ph.D., Sasha E. Larsen, Ph.D., Evan Cross, B.Sc., Tiffany Pecor, B.S., Thomas Smytheman, B.Sc., Emma Johnson, B.S., Valerie A. Reese, M.S., Susan L. Baldwin, Ph.D., Brittany Williams, B.Sc., Suhavi Kaur, B.Sc., Zhiyi Zhu, Ph.D.

University of Rochester Medical Center, Rochester, NY, VTEU

Angela R. Branche, M.D., David Dobrzynski, M.D., Ann R. Falsey, M.D., Ian Shannon, M.S., R.N., Patrick Kingsley, Arthur Zemanek, R.N., Katherine Elena, R.N., Samuel Diehl, Spencer Obrecht, R.N., Amy Kaychalo, Tanya, Smith, Erin Nowicki, Sharon Moorehead, Kari Steinmetz, C.C.R.C., Doreen Francis, R.N., C.C.R.C., Jeanne Holden-Wiltse, M.P.H., M.B.A., Christopher Lane, Michael Peasley, Kyle Richards, Pharm.D., Nicole Dornbush, Pharm.D., Carol Cole, Pharm.D.

The Hope Clinic of Emory University (Hope), Atlanta, GA, VTEU

Srilatha Edupuganti, M.D., M.P.H., Daniel Graciaa, M.D., Pauline Rebolledo, MD, Zanthia Wiley, MD, Nadine Rouphael, M.D., M.Sc., Alexis Ahonen, M.S.N., Alicarmen Alvarez, R.N., Amy Anderson, R.N., Mary Atha, M.S.N., Sarah Bechnak, R.N., Mary Bower, R.N., Ellie Butler, Laura Clegg, R.N., Carla N. Cooke, P.A., Sharon Curate-Ingram, R.N., Renata Lynn Dennis, M.P.H., R.N., Francine Dyer, R.N., Srilatha Edupuganti, M.D., M.P.H., Tigisty Girmary,

R.N., Rebecca Gonzalez, Pharm.D., Natalie Gray, Cassie Grimsley-Ackerley, M.D., M.Sc., Lauren Hewitt, L.P.N., Christopher Huerta, Brandi Johnson, Colleen Kelley, M.D., M.P.H., Athena Koumanelis, Deborah Laryea, R.N., Cecilia Losada, Hollie Macenczak, R.N., Michelle Piane McCullough, Eileen Osinski, Bernadine Panganiban, Varun Phadke, M.D., Paulina A. Rebolledo, M.D., M.Sc., Nadine Rouphael, M.D., M.Sc., Erin Scherer, Ph.D., Jessica Traenkner, P.A., Kristen Unterberger, P.A., Jacob Usher, Michelle Wiles, R.N., T. Jean Winter, Jianguo Xu, R.Ph., Ph.D., Yongxian Xu

Emory Children's Center (ECC), Atlanta, GA, VTEU
Christina Rostad, M.D., Evan J. Anderson, M.D., Satoshi Kamidani, M.D., Amari Barrett, Julia Bartol, Leisa Bower, R.N., Jessica Bowman, R.N., Andres F. Camacho-Gonzalez, M.D., M.Sc., Victoria Curry, R.N., Alexandria Dreyer, N.P., Khadijah Francois, Theda Gibson, M.S., Felicia Glover, Cindy Hardison, R.N., Hui-Mien Hsiao, M.S., Amberly Hunter, Laila Hussaini, M.P.H., Inara Jooma, Peggy Kettle, R.N., Marcia Lewis, R.N., Wensheng Li, Cindy Lubbers, R.N., Lisa Macoy, R.N., M.S.N., Clair Martin, Molly Morrison, Amy Muchinsky, Lauren Nolan, P.A., Heather Nurse, R.N., Etza Peters, R.N., Sarah Pinheiro, Susan Rogers, R.Ph., Amber Samuel, Anna Siaw-Anim, Maya Stagg, Kathy Stephens, R.N., M.S.N., Kathryn Zaks, M.S.

University of Pittsburgh School of Medicine, Pittsburgh, PA, subsidiary to Vanderbilt University VTEU, Nashville, TN
Judith M. Martin, M.D., Kumaravel Rajakumar, M.D., M.S., Gysella B. Muniz M.D., Alejandro Hoberman, M.D., Timothy Shope M.D., M.P.H., Nader Shaikh, M.D., M.P.H., , Jennifer Opal R.N., Melissa Andrasko R.N., Emma Cribbs R.N., Amber Koah R.N., Kimberly McMurtry PharmD, MaryAnn Sieber R.N., John F. Alcorn Ph.D., Flavia Rago Goncalves Ph.D.

University of Washington, Seattle, WA, VTEU
Christine Johnston, M.D., M.P.H., Tara M. Babu, M.D., M.Sci., Anna Wald, M.D., M.P.H., Morissa Pertik, PA-C, T. Nui Pholsena, ARNP, Jina Taub, ARNP, Dana Varon, ARNP; Meredith Potochnic, Pharm.D.; Matthew Dustrude, Kerry Laing, Ph.D., David M. Koelle, M.D.; Alyssa Braun, Anya Mathur, Dolly Singh, Jessica Heimonen, Jessica Moreno, Linsey McClellan, Maddie Humphreys, Ray Larsen, Taylor Krause, Mary Kirk, M.P.H., Matthew Seymour, M.P.H., Kirsten Hauge, M.P.H., Lawrence Hemingway, Christopher McClurkan, Max Krist, Victoria Campbell

University of Texas Medical Branch (UTMB), League City, TX, subsidiary to BCM VTEU
Richard E. Rupp, M.D., Megan Berman, M.D., Laura Porterfield, M.D., Amber Stanford, PA-C, Kristin Pollock, R.N., Robert Cox, R.N., Hala Ghoson, Pharm.D., Claire Marsh, B.S.N., R.N., Amy McMahan, L.V.N., Esther Cox, Diane Barrett, M.S.

NYU VETU, New York, NY
New York University – Langone Vaccine Center, Manhattan, New York, NY, VTEU
Mark J. Mulligan, M.D., Ramin Herati, M.D., Vijaya Soma, M.D., Lalitha Parameswaran, M.D., Alexander McMeeking, M.D., Mary Olson, N.P., Celia Engelson, N.P., Tamia Davis, N.P., Irma Noriega, N.P., Heekoung Youn, R.N., Samantha Yip, R.N., Jacqueline Callahan, R.N., Aimee Edwin, R.N., Marie Samanovic, Ph.D., Michael Tuen, Amber Cornelius, M.S., Abdonnie Holder, James Wilson, Meron Tasissa, Shelby Goins, Trishala Karmacharya, Hibah Khan, Sajjad Hussein, Doaa Ayoubi, Pharm.D., Ph.D., Brian Ta, Philip Aziz, Pharm.D., Sadia Minhas,

Pharm.D., Doris Wong, Pharm.D., Mahnoor Ali, Pamela Suman, Kibret Yohannes, Lisa Zhao, Abdulwahab Abdulai, Amanda Dontino, Milani Yonatan.

NYU Langone Vaccine Center Research Clinic at Bellevue Hospital, New York, NY - subsidiary to NYU VTEU:
Angelica Kottkamp, M.D., Jennifer Dong, M.D., Damian Inlall, Ellie Carmody, M.D., Rebecca Boas, M.D., Jennifer Knishinsky, M.D., Reza Parungao, M.D., Adam Schwartz, M.D., Melinda Katz, M.D., Natella Aronova, N.P., Rita Mennuti, N.P., Athina Agbayani, R.N., Siham Akleh, R.N., Zeeshan Iqbal, Julia Wagner, Venissala Wongchai, Tiffany Salcito, Hye-Youn Kim, Leeja Abraham, Denise Dong, Lance Goodman, Nadia Tadros, Danhong Jiang, Anna Jacobs, Pharm.D., Alina Neganova, R.N.

New York University- Long Island Vaccine Center, Mineola, NY, subsidiary to NYU VTEU
Martín Bäcker, M.D., Kimberly Byrnes, LPN CCRC, Sajumon Joseph, N.P., Asif Noor, M.D., Andrew B. Fleming, M.D., Sigridh Muñoz-Gómez, M.D., Diana Badillo, M.D., Sarah J. Pastolero, R.N., Maung Aung, Alicia Vasile, B.S., R.Ph., April Correll, R.Ph., Steven E. Carsons, M.D., Louis Ragolia, Ph.D., Christopher Hall, Thomas Palaia, Lavern Harvey.

Cincinnati Children's Hospital Medical Center (CCHMC), Cincinnati, OH, VTEU
Rebecca C. Brady, M.D.; Robert W. Frenck, M.D.; Paul W. Spearman, M.D.; Grant C. Paulsen, M.D.; Eleanor Widdice, M.D.; Felicia A. Scaggs Huang, M.D.; Michelle Dickey, A.P.R.N.; Kristen Buschle, A.P.R.N.; Vivian Mulholland, R.N.; Susan Parker, R.N.; ; Margery Huron, R.N.; Sally McCartney, R.N.; Jamie Kidd, R.N.; Jennifer Whitaker, R.N.; Marian Crossman, R.N.; Kristie Price, Pharm.D.; Sarah Boland, R.Ph.; Jesse LePage, B.S.; Monica Malone McNeal, M.S.; Laura Pace, B.A.; Theresa Baker, M.S.; Robert Zoellner, B.S.; Mary Pat McKee, M.S.; Nicole Vollman, B.A.; Christina Quigley, Ph.D.

FHI360, Durham, NC
Janet I. Archer M.Sc., Linda McNeil MA PMP, Mary Briggs, Katlyn Hurst B.Sc ., Latrisha Farley B.S., M.B.A., Marcy Steigerwald B.S.

IDCRC Leadership and Administrative Team
Kathleen M. Neuzil, M.D., David S. Stephens, M.D., Monica M. Farley M.D., Jeanne Marrazzo, M.D., Robert L. Atmar M.D., Jeffery Lennox, M.D., Sidnee Paschal Young, MALS; Barbara E. Walsh, Kayla Smith, MPH; and Bridget A. Wynn

IDCRC Statistical and Data Science Unit (SDSU), Vaccine and Infectious Disease Division, Fred Hutchinson Cancer Research Center, Seattle, WA:
Elizabeth R. Brown, Sc.D., Clara Dominguez Islas, Ph.D.

Statistical Center for HIV/AIDS Research and Prevention (SCHARP), Fred Hutchinson Cancer Research Center, Seattle, WA:
Jillian Zemanek, M.P.H., Daniel Szydlo, M.S., Rahul PaulChoudhury, M.P.H., M.S., Brian Ingersoll, Wen-Min (Wendy) Hou, M.P.H., B.S.N, C.C.R.P., Kelly Maddox, Chloe Waters, Lauren Young, M.P.H., Karan A. Shah, M.S., Anisa C. Gravelle, M.S., Craig N Chin, Drew Edwards, Rudie Desravines, M.S., Mark Trumbull, M.S., Srikanth Nooney, Udhab Adhikari, Josh Larkin, Jackie Benson, Christine Thompson, Jean-Paul Pease.

IDCRC Laboratory Operations Unit (LOU): Christine M. Posavad, PhD; John Hural, PhD; Michael Stirewalt; Megan Meagher; Weston Lawler; Chyndhri Padmanabhan

Division of Microbiology and Infectious Diseases, National Institute of Allergy and Infectious Diseases, National Institutes of Health, Bethesda, MD.
Marina Lee, Ph.D., Mohamed Elsafy, M.D., Rhonda Pikaart-Tautges, B.S. Janice Arega, M.S., Binh Hoang, R. Ph., Dan Curtin, Olivia Sparer, B.A., Ranjodh Gill M.P.H, Hyung Koo, B.S.N., Elisa Sindall, B.S.N., Sonja Crandon, B.S.N., Seema Nayak, M.D., Diane J. Post, Ph. D., Paul C Roberts, Ph.D., John Beigel, M.D.

Vaccine Immunology Program, Vaccine Research Center, NIAID, NIH, Bethesda, MD.
Leonid Serebryannyy, Ph.D., Bob C. Lin

Thermo Fisher Scientific, Germantown, MD.
Jim Dunn

Fred Hutch, Seattle, WA (ICS)
M. Juliana McElrath, MD, PhD, Kristen W. Cohen, PhD, Stephen De Rosa, MD, Carol Marty, Todd Haight

Emory University Laboratory (FRNT), Atlanta, GA
Mehul S. Suthar, Ph.D., Lilin Lai, M.D., Katharine Floyd, B.S., Madison Ellis, B.S., Kathryn M. Moore, Ph.D., Kelly Manning, M.S., M.P.H., Stephanie L. Foster, Ph.D., B.S., Mit Patel

Duke University Laboratory – Durham, NC
David Montefiori, Ph.D., Amanda Eaton, M.B.A.

Supplementary Figure 1: CONSORT diagram. Disposition of participants enrolled in the study to receive a Novavax NVX-CoV1373 boost vaccination.

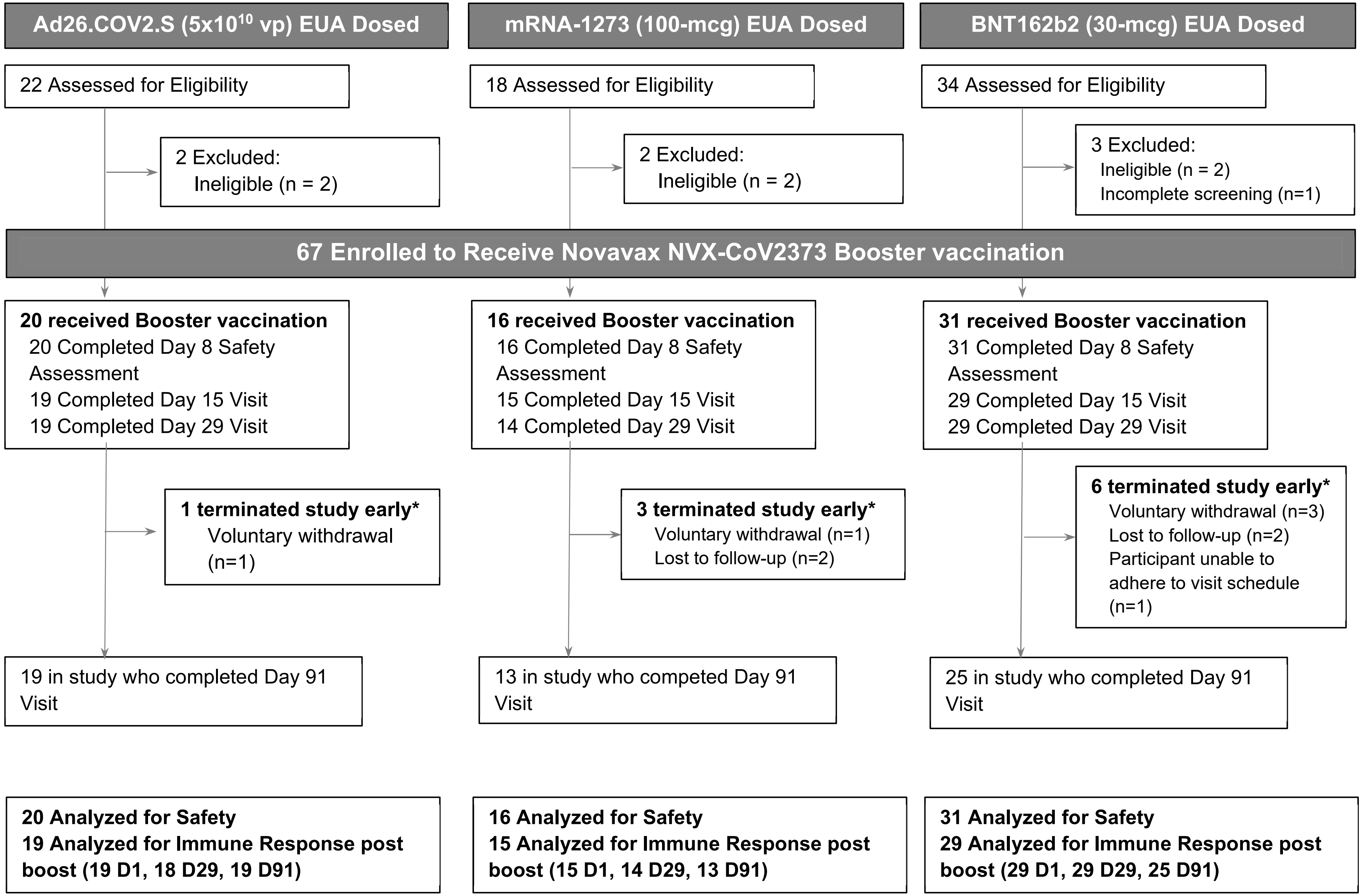

** Participants with (i) early termination recorded before Day 91 or (ii) early termination recorded after Day 91 but with last completed visit before Day 91.*

Supplementary Figure 2: Pseudovirus neutralizing antibody (PsVNA) geometric mean ID50 titers (with 95% CIs) by study day and spike variant for persons primed with (A) Ad26.COV2.S, (B) mRNA-1273, or (C) BMT162b2 SARS CoV-2 vaccines.

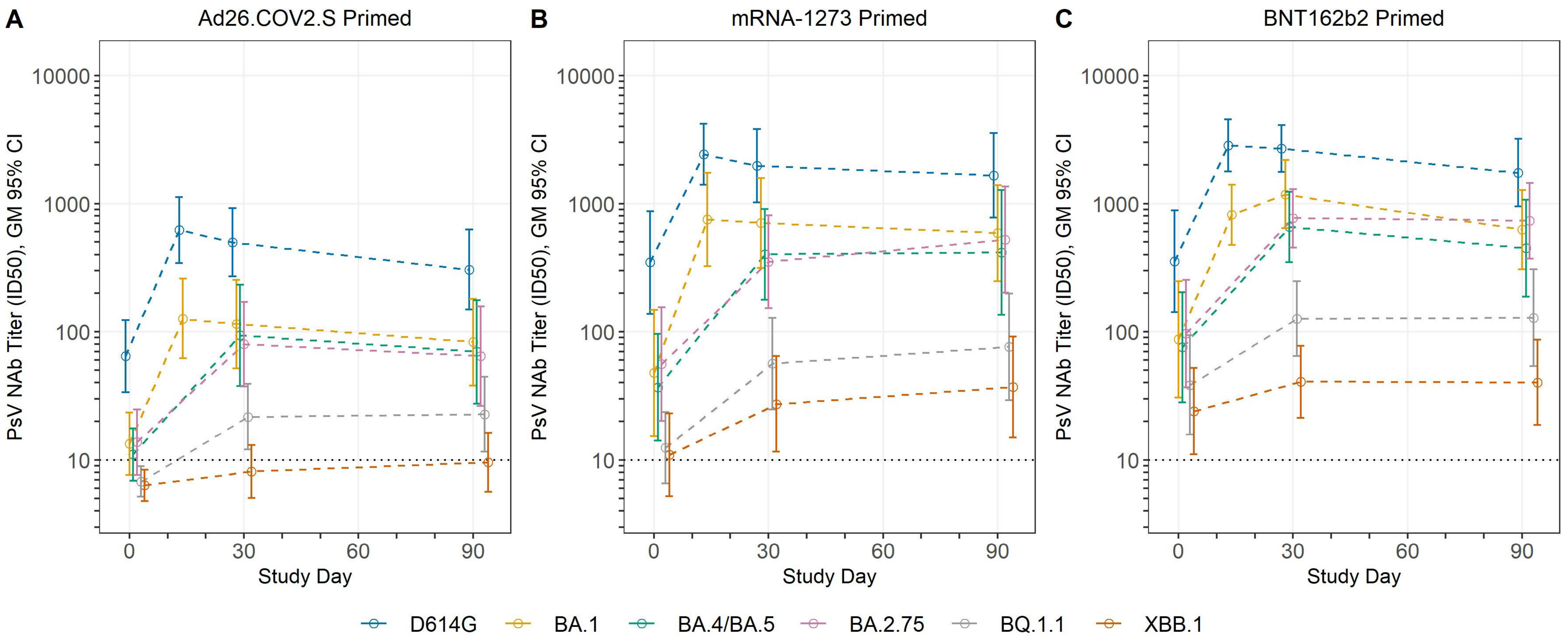

Supplementary Figure 3: Pseudovirus neutralizing antibody (PsVNA) ID50 titers by subject, infection status, study day and spike variant for persons primed with (A) Ad26.COV2.S, (B) mRNA-1273, or (C) BMT162b2 SARS CoV-2 vaccines.

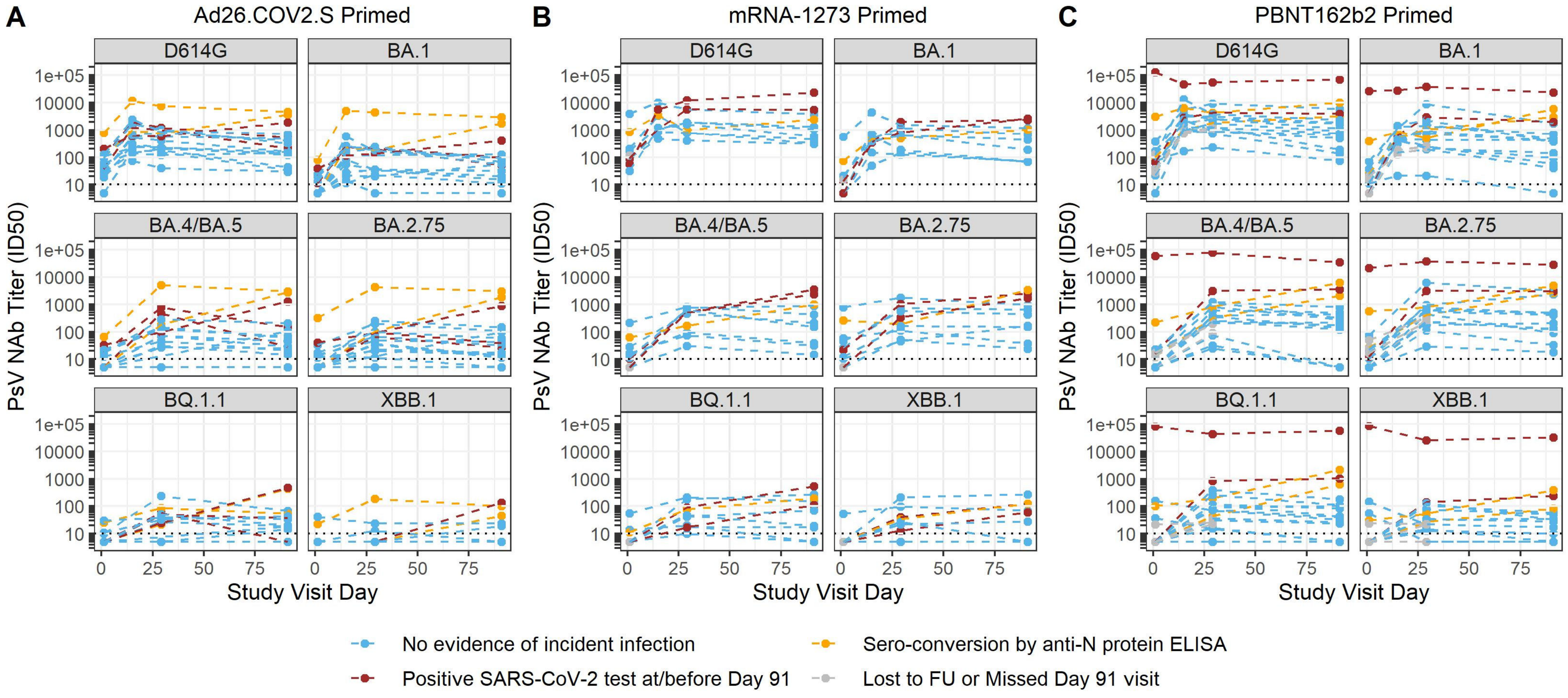

Note: This figure excludes those participants N antibody positive at baseline; of the seven participants with positive SARS-CoV-2 tests at/before Day 91 visit, 4 showed N-protein Ab at Day 91. Two volunteers had a positive SARS-CoV-2 test on Day 91 but remained N-protein Ab negative until Day 181 (not shown). One volunteer remained N-protein Ab negative at Day 181.

Supplementary Figure 4: CD4+ and CD8+ T-cell responses. Spike-specific T cell levels for (A) CD4+ Th1 (expressing interferon-gamma [IFNγ], interleukin-2 [IL-2] or both) Tcells; (B) CD4+ Th2 cells (expressing IL-4, IL-5 and/or IL-13 and co-expressing CD40L) T cells; and (C) CD8+ T cells (expressing IFN-γ and/or IL-2) depicted as box plots. Those participants with baseline anti-N Ab are shown in red. Dashed lines represent individual changes from study day 1 to day 15. Numbers represent number with a positive result/number tested.

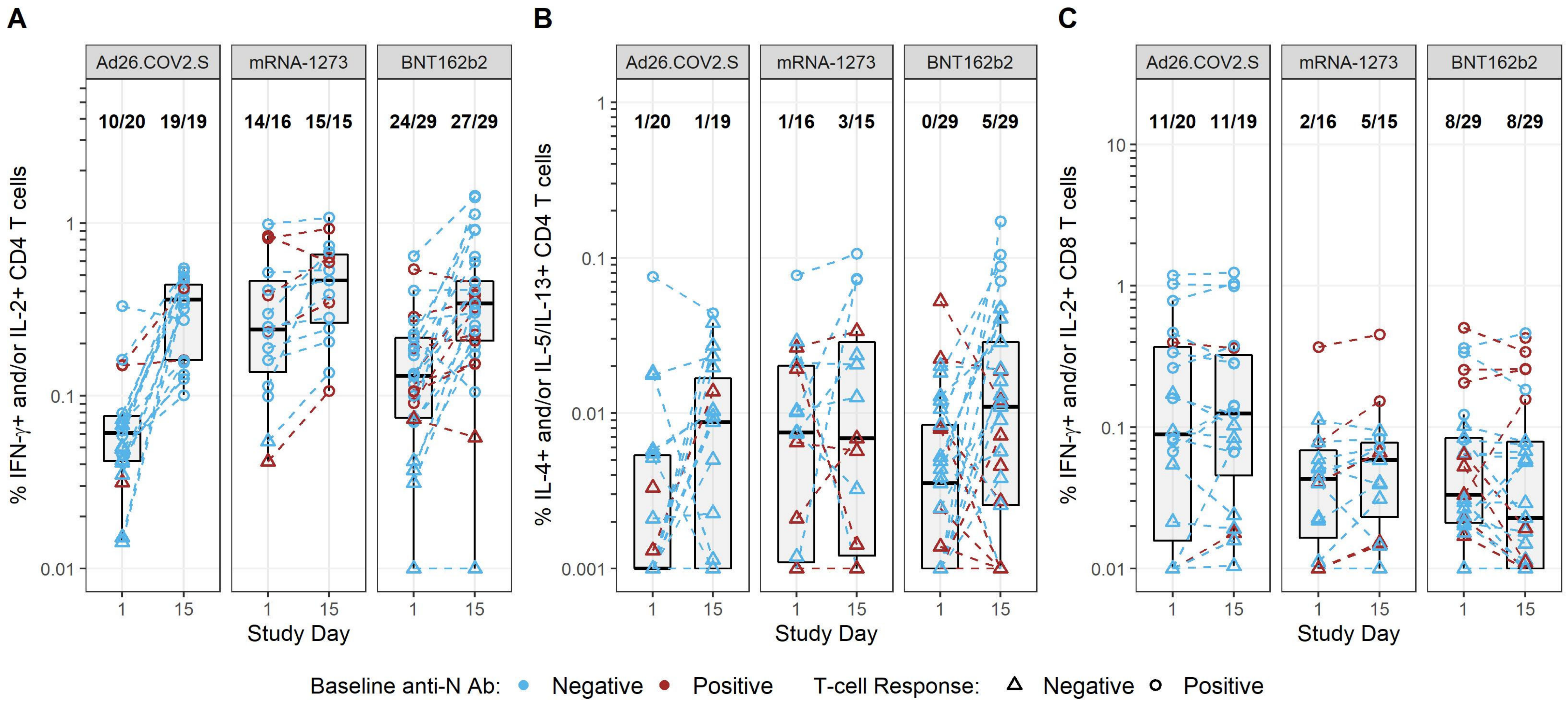

Supplementary Table 1: Solicited reactogenicity by study group*

	Priming regimen (number)		
Reaction	Ad26.COV2.S (n = 20)	mRNA-1273 (n = 16)	BNT162b2 (n = 31)
Injection site	Mild/Moderate/Severe	Mild/Moderate/Severe	Mild/Moderate/Severe
Erythema/redness	0/0/0	0/0/0	2/0/0
Induration/swelling	1/0/0	0/0/0	0/1/0
Pain/tenderness	11/0/0	3/1/1	13/2/0
Systemic			
Malaise/fatigue	9/4/0	4/2/1	7/6/0
Myalgia	7/1/0	3/0/1	14/2/0
Headache	7/3/0	3/0/0	4/1/0
Nausea	4/1/0	3/1/0	0/1/0
Chills	1/1/0	1/1/0	4/0/0
Arthralgia	3/0/0	2/1/0	3/1/0
Fever	0/0/0	0/0/0	0/0/0

*Reactogenicity as determined by FDA Toxicity Grading Scale for Healthy Adults

Supplementary Table 2: Participants experiencing related and unrelated unsolicited adverse events up to Day 91 of the study, reported overall and for each Preferred Term (MedDRA code classification), by study group, and maximum severity.

	**Priming regimen (number)**		
**MedDRA Preferred Term classification**	**Ad26.COV2.S (n=20)**	**mRNA-1273 (n=16)**	**BNT162b2 (n=31)**
Participants reporting at least one **related AE**			
Overall	3 mild<br>0 moderate<br>0 severe	0 mild<br>1 moderate<br>0 severe	2 mild<br>0 moderate<br>0 severe
Lymphadenopathy	2 mild	0	0
Axillary pain	0	0	1 mild
Neck pain	0	0	1 mild
Dizziness	1 mild	0	0
Night sweats	0	1 moderate	0
Participants reporting at least one **unrelated AE**			
Overall	3 mild<br>3 moderate<br>1 severe	2 mild<br>2 moderate<br>1 severe	6 mild<br>2 moderate<br>1 severe<br>1 potentially life-threatening
Micrognathia	0	1 mild	0
Food poisoning	0	0	1 mild
Injection site hemorrhage	1 mild	0	0
Cholelithiasis	1 moderate	0	0
COVID-19	0	0	2 mild
Viral respiratory tract infection	0	0	1 mild
Upper respiratory tract infection	1 mild	0	0
Arthropod bite	0	0	1 mild
Heat stroke	0	0	1 potentially life-threatening
Ligament rupture	0	0	1 mild
Ligament strain	0	0	1 mild
Muscle strain	0	1 moderate	0
Elevated diastolic blood pressure	1 severe	0	1 mild
Elevated blood pressure	1 mild	1 severe	1 severe
Elevated systolic blood pressure	2 mild	0	1 mild
Decreased heart rate	0	1 mild	0
Arthralgia	1 mild	0	0
Musculoskeletal stiffness	0	1 moderate	1 moderate
Dizziness	0	0	1 mild
Sciatica	0	0	1 moderate
Seizure	0	0	1 potentially life-threatening
Vaginal hemorrhage	1 moderate	0	0
Dry throat	0	0	1 mild
Rhinorrhea	1 moderate	0	0

Supplementary Table 3: IgG Serum Binding Antibody Response to S-2P-WA-1- IgG Serum Binding Antibody Response to S-2P-WA-1[1] Antigen by 4-plex ECLIA V.2, by Group and Timepoint - Results are reported in Arbitrary Units (AU/mL)

	Group 15E [Dosed Janssen, Boost Novavax] (N=20)	Group 16E [Dosed Moderna, Boost Novavax] (N=16)	Group 17E [Dosed Pfizer, Boost Novavax] (N=31)
**Day 1 Visit (Pre-boost)**			
N (non-missing)	20	16	31
Median (P_{25}, P_{75}), AU/mL	10373.13 (8060.81-32526.97)	65092.05 (25622.86-205833.85)	44439.85 (18729.05-318619.52)
Minimum - Maximum, AU/mL	881.91-83475.95	10632.23-1844666.14	3900.34-1757721.04
Geometric Mean (95% CI), AU/mL	12557.15 (7025.54-22444.12)	74443.97 (36420.46-152164.61)	63882.77 (34292.75-119005.00)
**Day 15 Visit (14 days post-boost)**			
N (non-missing)	19	15	29
Median (P_{25}, P_{75}), AU/mL	146076.16 (49261.36-259582.10)	326541.44 (210363.05-587599.72)	302269.61 (205689.44-500575.28)
Minimum - Maximum, AU/mL	13025.84-475200.23	144568.58-1970839.91	69101.43-2437065.08
Geometric Mean (95% CI), AU/mL	106456.56 (64175.13-176594.88)	379229.00 (250871.33-573260.54)	347764.27 (248962.39-485776.12)
N* (non-missing pre- and post-boost)	19	15	29
Participants with 2-fold rise[2], 95% CI	94.7% (74.0%-99.9%)	80.0% (51.9%-95.7%)	69.0% (49.2%-84.7%)
Participants with 4-fold rise[2], 95% CI	68.4% (43.4%-87.4%)	53.3% (26.6%-78.7%)	55.2% (35.7%-73.6%)
Geometric Mean Fold Rise[2], 95% CI	7.78 (4.76-12.72)	5.09 (2.84-9.12)	5.68 (3.43-9.40)
**Day 29 Visit (28 days post-boost)**			
N (non-missing)	18	14	29
Median (P_{25}, P_{75}), AU/mL	122832.89 (42775.97-183330.38)	270570.04 (219961.96-567224.37)	296805.69 (230510.52-550595.37)
Minimum - Maximum, AU/mL	7727.43-480272.08	115904.54-1718955.28	66078.17-3165292.78
Geometric Mean (95% CI), AU/mL	85100.61 (50759.23-142675.79)	352744.33 (222418.81-559433.65)	350684.75 (254371.85-483464.65)
N* (non-missing pre- and post-boost)	18	14	29
Participants with 2-fold rise[2], 95% CI	88.9% (65.3%-98.6%)	78.6% (49.2%-95.3%)	72.4% (52.8%-87.3%)
Participants with 4-fold rise[2], 95% CI	72.2% (46.5%-90.3%)	50.0% (23.0%-77.0%)	55.2% (35.7%-73.6%)
Geometric Mean Fold Rise[2], 95% CI	6.04 (3.89-9.39)	5.96 (2.86-12.42)	5.73 (3.32-9.89)
**Day 91 Visit (3 months post-boost)**			
N (non-missing)	19	13	25
Median (P_{25}, P_{75}), AU/mL	50932.92 (14135.83-108266.32)	217386.57 (130543.53-480954.80)	224552.69 (142885.68-343044.98)
Minimum - Maximum, AU/mL	4632.08-482583.77	12093.60-3263584.27	22468.83-2786966.72

	Group 15E [Dosed Janssen, Boost Novavax] (N=20)	Group 16E [Dosed Moderna, Boost Novavax] (N=16)	Group 17E [Dosed Pfizer, Boost Novavax] (N=31)
Geometric Mean (95% CI), AU/mL	48361.23 (26217.02-89209.56)	227195.55 (97916.16-527163.42)	257385.26 (163253.73-405792.69)
N* (non-missing pre- and post-boost)	19	13	25
Participants with 2-fold rise[2], 95% CI	78.9% (54.4%-93.9%)	53.8% (25.1%-80.8%)	64.0% (42.5%-82.0%)
Participants with 4-fold rise[2], 95% CI	31.6% (12.6%-56.6%)	46.2% (19.2%-74.9%)	52.0% (31.3%-72.2%)
Geometric Mean Fold Rise[2], 95% CI	3.53 (2.30-5.43)	4.01 (1.50-10.73)	3.47 (1.88-6.38)

[1] Antibody values below the lower limit of quantification (LLOQ = 34.18 AU/mL) were assigned a value equal to LLOQ/2. Values greater than the upper limit of quantification (ULOQ = 19136250 AU/mL) are taken as reported, or a ceiling value equivalent to the ULOQ is assigned if values are not provided.

[2] Relative to pre-vaccination (Day 1 Visit) levels, among participants with no missing observations at both pre- and post-boost timepoints.

Supplementary Table 4: IgG Serum Binding Antibody Response to S-2P-WA-1 by Age - IgG Serum Binding Antibody Response to S-2P-WA-1[1] Antigen by 4-plex ECLIA V.2, by Group, Age Group and Timepoint - Results are reported in Arbitrary Units (AU/mL)

	Group 15E [Dosed Janssen, Boost Novavax] Age 18-55 yo (N=11)	Group 15E [Dosed Janssen, Boost Novavax] Age ≥56 yo (N=9)	Group 16E [Dosed Moderna, Boost Novavax] Age 18-55 yo (N=9)	Group 16E [Dosed Moderna, Boost Novavax] Age ≥56 yo (N=7)	Group 17E [Dosed Pfizer, Boost Novavax] Age 18-55 yo (N=25)	Group 17E [Dosed Pfizer, Boost Novavax] Age ≥56 yo (N=6)
**Day 1 Visit (Pre-boost)**						
N (non-missing)	11	9	9	7	25	6
Median (P_{25}, P_{75}), AU/mL	19196.97 (7934.56-73759.03)	9148.26 (8284.87-11542.29)	67843.31 (21053.27-244272.50)	62340.78 (27650.36-167395.20)	48680.01 (24545.72-318619.52)	16160.57 (4333.84-74518.17)
Minimum - Maximum, AU/mL	1426.98-83475.95	881.91-24045.05	10632.23-1844666.14	23595.36-330373.00	8760.79-1757721.04	3900.34-422014.74
Geometric Mean (95% CI), AU/mL	17207.81 (6669.23-44399.26)	8543.76 (4179.62-17464.72)	81237.93 (23288.63-283383.01)	66537.01 (26997.65-163983.65)	81945.03 (42168.27-159242.69)	22636.28 (3440.19-148945.77)
**Day 15 Visit (14 days post-boost)**						
N (non-missing)	10	9	9	6	23	6
Median (P_{25}, P_{75}), AU/mL	163809.52 (116024.08-214062.46)	99013.93 (39637.38-259582.10)	348079.78 (275932.07-511191.75)	268687.10 (157355.83-987353.35)	341578.56 (205689.44-696168.13)	229428.52 (167620.71-409100.81)
Minimum - Maximum, AU/mL	13195.56-475200.23	13025.84-296580.88	200075.59-1970839.91	144568.58-1051360.08	69101.43-2437065.08	155631.62-428973.98
Geometric Mean (95% CI), AU/mL	129899.07 (63558.47-265484.21)	85336.55 (36092.94-201765.94)	403617.94 (236788.66-687986.66)	345380.78 (137422.57-868037.06)	379328.62 (251591.31-571920.42)	249258.95 (157998.87-393230.83)
N* (non-missing pre- and post-boost)	10	9	9	6	23	6
Participants with 2-fold rise[2], 95% CI	100.0% (69.2%-100.0%)	88.9% (51.8%-99.7%)	77.8% (40.0%-97.2%)	83.3% (35.9%-99.6%)	65.2% (42.7%-83.6%)	83.3% (35.9%-99.6%)
Participants with 4-fold rise[2], 95% CI	70.0% (34.8%-93.3%)	66.7% (29.9%-92.5%)	55.6% (21.2%-86.3%)	50.0% (11.8%-88.2%)	47.8% (26.8%-69.4%)	83.3% (35.9%-99.6%)
Geometric Mean Fold Rise[2], 95% CI	6.21 (3.82-10.10)	9.99 (3.68-27.11)	4.97 (2.12-11.64)	5.29 (1.73-16.17)	4.78 (2.75-8.32)	11.01 (2.56-47.36)
**Day 29 Visit (28 days post-boost)**						
N (non-missing)	9	9	8	6	23	6
Median (P_{25}, P_{75}), AU/mL	126584.20 (80543.57-211222.49)	49019.17 (42775.97-162976.54)	334985.93 (233585.47-513456.74)	243844.98 (172301.32-1141807.80)	316625.63 (230510.52-573063.20)	278540.07 (195174.83-296805.69)
Minimum - Maximum, AU/mL	7727.43-480272.08	18520.42-197629.30	115904.54-893627.76	141482.31-1718955.28	66078.17-3165292.78	114762.81-429115.44
Geometric Mean (95% CI), AU/mL	104698.05 (41738.89-262625.15)	69171.43 (35291.53-135576.04)	335966.57 (198631.98-568254.58)	376425.20 (125429.01-1129690.29)	384717.45 (260220.22-568777.91)	245878.77 (153293.90-394382.09)
N* (non-missing pre- and post-boost)	9	9	8	6	23	6
Participants with 2-fold rise[2], 95% CI	77.8% (40.0%-97.2%)	100.0% (66.4%-100.0%)	75.0% (34.9%-96.8%)	83.3% (35.9%-99.6%)	69.6% (47.1%-86.8%)	83.3% (35.9%-99.6%)
Participants with 4-fold rise[2], 95% CI	66.7% (29.9%-92.5%)	77.8% (40.0%-97.2%)	50.0% (15.7%-84.3%)	50.0% (11.8%-88.2%)	47.8% (26.8%-69.4%)	83.3% (35.9%-99.6%)
Geometric Mean Fold Rise[2], 95% CI	4.51 (2.69-7.57)	8.10 (3.72-17.61)	6.11 (1.95-19.18)	5.76 (1.54-21.55)	4.85 (2.65-8.87)	10.86 (2.20-53.62)

	Group 15E [Dosed Janssen, Boost Novavax] Age 18-55 yo (N=11)	Group 15E [Dosed Janssen, Boost Novavax] Age ≥56 yo (N=9)	Group 16E [Dosed Moderna, Boost Novavax] Age 18-55 yo (N=9)	Group 16E [Dosed Moderna, Boost Novavax] Age ≥56 yo (N=7)	Group 17E [Dosed Pfizer, Boost Novavax] Age 18-55 yo (N=25)	Group 17E [Dosed Pfizer, Boost Novavax] Age ≥56 yo (N=6)
**Day 91 Visit (3 months post-boost)**						
N (non-missing)	10	9	7	6	20	5
Median (P_{25}, P_{75}), AU/mL	90833.04 (50932.92-221816.66)	25281.83 (13463.03-45848.45)	217386.57 (61105.09-480954.80)	202162.89 (130543.53-512664.75)	283285.44 (184633.97-700369.69)	124865.04 (47062.48-209083.81)
Minimum - Maximum, AU/mL	4632.08-482583.77	8966.31-87060.98	12093.60-1480868.62	120432.83-3263584.27	82692.49-2786966.72	22468.83-213767.68
Geometric Mean (95% CI), AU/mL	83351.88 (31351.27-221603.02)	26412.57 (13591.63-51327.48)	170341.89 (41248.62-703450.47)	317924.90 (84902.20-1190502.01)	334719.99 (211234.61-530393.54)	89989.47 (26357.40-307242.14)
N* (non-missing pre- and post-boost)	10	9	7	6	20	5
Participants with 2-fold rise[2], 95% CI	90.0% (55.5%-99.7%)	66.7% (29.9%-92.5%)	42.9% (9.9%-81.6%)	66.7% (22.3%-95.7%)	60.0% (36.1%-80.9%)	80.0% (28.4%-99.5%)
Participants with 4-fold rise[2], 95% CI	30.0% (6.7%-65.2%)	33.3% (7.5%-70.1%)	42.9% (9.9%-81.6%)	50.0% (11.8%-88.2%)	50.0% (27.2%-72.8%)	60.0% (14.7%-94.7%)
Geometric Mean Fold Rise[2], 95% CI	3.99 (1.89-8.42)	3.09 (1.78-5.36)	3.40 (0.65-17.95)	4.86 (0.96-24.65)	3.44 (1.70-6.97)	3.58 (0.55-23.36)

[1] Antibody values below the lower limit of quantification (LLOQ = 34.18 AU/mL) were assigned a value equal to LLOQ/2. Values greater than the upper limit of quantification (ULOQ = 19136250 AU/mL) are taken as reported, or a ceiling value equivalent to the ULOQ is assigned if values are not provided.
[2] Relative to pre-vaccination (Day 1 Visit) levels, among participants with no missing observations at both pre- and post-boost timepoints.

Supplementary Table 5: IgG Serum Binding Antibody Response to S-2P-B.1.351 - IgG Serum Binding Antibody Response to S-2P-B.1.351[1] Antigen by 4-plex ECLIA V.2, by Group and Timepoint

	Group 15E [Dosed Janssen, Boost Novavax] (N=20)	Group 16E [Dosed Moderna, Boost Novavax] (N=16)	Group 17E [Dosed Pfizer, Boost Novavax] (N=31)
**Day 1 Visit (Pre-boost)**			
N (non-missing)	20	16	31
Median (P_{25}, P_{75}), AU/mL	4056.37 (3039.46-8265.83)	27210.71 (10202.69-82185.54)	24758.17 (9843.99-177320.22)
Minimum - Maximum, AU/mL	484.25-45707.28	5821.21-762278.99	1714.23-963126.14
Geometric Mean (95% CI), AU/mL	4982.89 (2962.68-8380.64)	31266.61 (15255.97-64079.88)	33970.36 (18291.78-63087.63)
GM Fold Decrease Relative to WA-1[2], 95% CI	2.52 (2.05-3.10)	2.38 (2.17-2.62)	1.88 (1.77-2.00)
**Day 15 Visit (14 days post-boost)**			
N (non-missing)	19	15	29
Median (P_{25}, P_{75}), AU/mL	71731.16 (28027.95-118369.29)	152527.12 (110356.83-302992.32)	173893.96 (102285.40-261001.45)
Minimum - Maximum, AU/mL	3477.28-176050.85	52452.84-738341.81	38030.39-1261412.33
Geometric Mean (95% CI), AU/mL	53011.83 (30464.72-92246.20)	168794.28 (114386.17-249081.75)	188301.06 (134647.46-263334.25)
GM Fold Decrease Relative to WA-1[2], 95% CI	2.01 (1.80-2.24)	2.25 (2.02-2.50)	1.85 (1.76-1.94)
N* (non-missing pre- and post-boost)	19	15	29
Participants with 2-fold rise[3], 95% CI	100.0% (82.4%-100.0%)	86.7% (59.5%-98.3%)	72.4% (52.8%-87.3%)
Participants with 4-fold rise[3], 95% CI	84.2% (60.4%-96.6%)	53.3% (26.6%-78.7%)	55.2% (35.7%-73.6%)
Geometric Mean Fold Rise[3], 95% CI	10.02 (6.28-15.97)	5.40 (3.06-9.54)	5.83 (3.60-9.46)
**Day 29 Visit (28 days post-boost)**			
N (non-missing)	18	14	29
Median (P_{25}, P_{75}), AU/mL	55621.02 (21755.63-86479.63)	130710.31 (103035.82-288706.02)	152001.96 (106966.64-293888.80)
Minimum - Maximum, AU/mL	3920.72-169320.54	56267.08-481898.29	34994.80-1521054.80
Geometric Mean (95% CI), AU/mL	39462.85 (22827.90-68219.85)	155985.12 (102267.23-237919.41)	180657.65 (130440.93-250206.65)
GM Fold Decrease Relative to WA-1[2], 95% CI	2.16 (1.91-2.44)	2.26 (1.98-2.59)	1.94 (1.83-2.06)
N* (non-missing pre- and post-boost)	18	14	29
Participants with 2-fold rise[3], 95% CI	94.4% (72.7%-99.9%)	85.7% (57.2%-98.2%)	72.4% (52.8%-87.3%)
Participants with 4-fold rise[3], 95% CI	77.8% (52.4%-93.6%)	57.1% (28.9%-82.3%)	55.2% (35.7%-73.6%)
Geometric Mean Fold Rise[3], 95% CI	7.20 (4.75-10.89)	6.27 (3.11-12.67)	5.60 (3.33-9.40)
**Day 91 Visit (3 months post-boost)**			

	Group 15E [Dosed Janssen, Boost Novavax] (N=20)	Group 16E [Dosed Moderna, Boost Novavax] (N=16)	Group 17E [Dosed Pfizer, Boost Novavax] (N=31)
N (non-missing)	19	13	25
Median (P_{25}, P_{75}), AU/mL	21536.03 (7819.36-47328.90)	104325.69 (50369.91-181673.55)	134674.76 (87291.12-165408.65)
Minimum - Maximum, AU/mL	2057.20-210453.13	6655.78-889069.35	10563.25-1670778.43
Geometric Mean (95% CI), AU/mL	21114.63 (11378.12-39182.88)	96873.73 (44354.95-211577.72)	133183.89 (84117.94-210869.96)
GM Fold Decrease Relative to WA-1[2], 95% CI	2.29 (2.02-2.60)	2.35 (2.06-2.67)	1.93 (1.83-2.04)
N* (non-missing pre- and post-boost)	19	13	25
Participants with 2-fold rise[3], 95% CI	73.7% (48.8%-90.9%)	61.5% (31.6%-86.1%)	64.0% (42.5%-82.0%)
Participants with 4-fold rise[3], 95% CI	52.6% (28.9%-75.6%)	46.2% (19.2%-74.9%)	52.0% (31.3%-72.2%)
Geometric Mean Fold Rise[3], 95% CI	3.99 (2.53-6.28)	4.11 (1.58-10.72)	3.35 (1.86-6.01)

[1] Antibody values below the lower limit of quantification (LLOQ = 10.254 AU/mL) were assigned a value equal to LLOQ/2. Values greater than the upper limit of quantification (ULOQ = 5740875 AU/mL) are taken as reported, or a ceiling value equivalent to the ULOQ is assigned if values are not provided.

[2] Relative to 4-plex WA-1antibody levels at same visit.

[3] Relative to pre-vaccination (Day 1 Visit) levels, among participants with no missing observations at both pre- and post-boost timepoints.

Supplementary Table 5: IgG Serum Binding Antibody Response to S-2P-B.1.351 by Age- IgG Serum Binding Antibody Response to S-2P-B.1.351[1] Antigen by 4-plex ECLIA V.2, by Group, Age Group and Timepoint

	Group 15E [Dosed Janssen, Boost Novavax] Age 18-55 yo (N=11)	Group 15E [Dosed Janssen, Boost Novavax] Age ≥56 yo (N=9)	Group 16E [Dosed Moderna, Boost Novavax] Age 18-55 yo (N=9)	Group 16E [Dosed Moderna, Boost Novavax] Age ≥56 yo (N=7)	Group 17E [Dosed Pfizer, Boost Novavax] Age 18-55 yo (N=25)	Group 17E [Dosed Pfizer, Boost Novavax] Age ≥56 yo (N=6)
**Day 1 Visit (Pre-boost)**						
N (non-missing)	11	9	9	7	25	6
Median (P_{25}, P_{75}), AU/mL	6549.21 (2788.66-21653.18)	3888.15 (3290.27-5792.02)	34730.09 (8837.99-83200.96)	22632.32 (11567.40-81170.11)	25929.75 (14855.01-196804.36)	7278.23 (2388.59-33641.46)
Minimum - Maximum, AU/mL	484.25-45707.28	1194.00-8486.33	5821.21-762278.99	8524.31-118920.92	3829.39-963126.14	1714.23-175144.23
Geometric Mean (95% CI), AU/mL	6355.65 (2550.92-15835.18)	3701.00 (2220.23-6169.36)	35121.58 (10090.54-122245.74)	26925.33 (10793.15-67169.76)	45156.93 (23525.19-86679.34)	10375.26 (1676.07-64225.14)
GM Fold Decrease Relative to WA-1[2], 95% CI	2.71 (2.12-3.45)	2.31 (1.52-3.50)	2.31 (1.96-2.73)	2.47 (2.22-2.75)	1.81 (1.69-1.94)	2.18 (1.97-2.42)
**Day 15 Visit (14 days post-boost)**						
N (non-missing)	10	9	9	6	23	6
Median (P_{25}, P_{75}), AU/mL	92380.03 (46299.98-115511.97)	48449.54 (16537.36-151321.68)	153178.47 (146405.05-231338.79)	136651.95 (61045.29-302992.32)	182629.96 (102285.40-365550.42)	120213.10 (91639.50-221529.62)
Minimum - Maximum, AU/mL	5838.59-166053.05	3477.28-176050.85	93873.47-738341.81	52452.84-410838.05	38030.39-1261412.33	64340.38-231199.68
Geometric Mean (95% CI), AU/mL	63828.81 (31416.17-129682.15)	43128.91 (15482.79-120140.00)	191744.78 (118744.97-309622.04)	139414.83 (58404.36-332791.86)	208342.76 (138836.89-312645.33)	127782.42 (75638.37-215873.85)
GM Fold Decrease Relative to WA-1[2], 95% CI	2.04 (1.77-2.34)	1.98 (1.61-2.44)	2.10 (1.87-2.38)	2.48 (1.97-3.11)	1.82 (1.75-1.90)	1.95 (1.54-2.47)
N* (non-missing pre- and post-boost)	10	9	9	6	23	6
Participants with 2-fold rise[3], 95% CI	100.0% (69.2%-100.0%)	100.0% (66.4%-100.0%)	88.9% (51.8%-99.7%)	83.3% (35.9%-99.6%)	69.6% (47.1%-86.8%)	83.3% (35.9%-99.6%)
Participants with 4-fold rise[3], 95% CI	80.0% (44.4%-97.5%)	88.9% (51.8%-99.7%)	55.6% (21.2%-86.3%)	50.0% (11.8%-88.2%)	47.8% (26.8%-69.4%)	83.3% (35.9%-99.6%)
Geometric Mean Fold Rise[3], 95% CI	8.74 (4.83-15.82)	11.65 (4.88-27.84)	5.46 (2.27-13.14)	5.32 (2.01-14.07)	4.80 (2.85-8.10)	12.32 (3.09-49.08)
**Day 29 Visit (28 days post-boost)**						
N (non-missing)	9	9	8	6	23	6
Median (P_{25}, P_{75}), AU/mL	64359.33 (29536.76-86479.63)	26372.75 (19027.73-83331.29)	150208.46 (116817.70-249547.34)	116856.69 (67023.63-411904.67)	165698.24 (123140.45-312995.45)	117470.57 (80646.59-158559.63)
Minimum - Maximum, AU/mL	3920.72-169320.54	4025.35-117194.71	59405.95-459487.32	56267.08-481898.29	34994.80-1521054.80	58938.99-253250.79
Geometric Mean (95% CI), AU/mL	46933.20 (19547.95-112683.16)	33181.55 (14252.40-77251.20)	163105.12 (96467.60-275774.23)	146972.90 (56492.53-382369.74)	202390.83 (137277.96-298387.66)	116880.18 (67573.15-202165.77)
GM Fold Decrease Relative to WA-1[2], 95% CI	2.23 (1.95-2.55)	2.08 (1.64-2.66)	2.06 (1.80-2.36)	2.56 (1.94-3.38)	1.90 (1.80-2.01)	2.10 (1.66-2.66)
N* (non-missing pre- and post-boost)	9	9	8	6	23	6
Participants with 2-fold rise[3], 95% CI	88.9% (51.8%-99.7%)	100.0% (66.4%-100.0%)	87.5% (47.3%-99.7%)	83.3% (35.9%-99.6%)	69.6% (47.1%-86.8%)	83.3% (35.9%-99.6%)

	Group 15E [Dosed Janssen, Boost Novavax] Age 18-55 yo (N=11)	Group 15E [Dosed Janssen, Boost Novavax] Age ≥56 yo (N=9)	Group 16E [Dosed Moderna, Boost Novavax] Age 18-55 yo (N=9)	Group 16E [Dosed Moderna, Boost Novavax] Age ≥56 yo (N=7)	Group 17E [Dosed Pfizer, Boost Novavax] Age 18-55 yo (N=25)	Group 17E [Dosed Pfizer, Boost Novavax] Age ≥56 yo (N=6)
Participants with 4-fold rise[3], 95% CI	66.7% (29.9%-92.5%)	88.9% (51.8%-99.7%)	50.0% (15.7%-84.3%)	66.7% (22.3%-95.7%)	47.8% (26.8%-69.4%)	83.3% (35.9%-99.6%)
Geometric Mean Fold Rise[3], 95% CI	5.78 (3.22-10.36)	8.97 (4.53-17.76)	6.82 (2.10-22.20)	5.61 (1.93-16.33)	4.66 (2.64-8.23)	11.27 (2.51-50.63)
**Day 91 Visit (3 months post-boost)**						
N (non-missing)	10	9	7	6	20	5
Median (P_{25}, P_{75}), AU/mL	38519.24 (19424.30-88357.79)	9268.45 (6538.76-21536.03)	122797.32 (32188.62-183245.26)	85634.27 (50369.91-181673.55)	159437.50 (101614.85-295247.45)	56558.65 (18940.88-117922.78)
Minimum - Maximum, AU/mL	2145.23-210453.13	2057.20-47328.90	6655.78-767438.12	44081.99-889069.35	43355.49-1670778.43	10563.25-134674.76
Geometric Mean (95% CI), AU/mL	35303.99 (13817.70-90201.11)	11927.20 (5384.18-26421.53)	82683.26 (20780.03-328994.88)	116535.75 (35900.17-378287.37)	174918.43 (111942.97-273321.84)	44762.68 (11121.09-180171.00)
GM Fold Decrease Relative to WA-1[2], 95% CI	2.36 (2.15-2.59)	2.21 (1.68-2.92)	2.06 (1.76-2.41)	2.73 (2.30-3.24)	1.91 (1.81-2.02)	2.01 (1.60-2.52)
N* (non-missing pre- and post-boost)	10	9	7	6	20	5
Participants with 2-fold rise[3], 95% CI	80.0% (44.4%-97.5%)	66.7% (29.9%-92.5%)	57.1% (18.4%-90.1%)	66.7% (22.3%-95.7%)	60.0% (36.1%-80.9%)	80.0% (28.4%-99.5%)
Participants with 4-fold rise[3], 95% CI	60.0% (26.2%-87.8%)	44.4% (13.7%-78.8%)	42.9% (9.9%-81.6%)	50.0% (11.8%-88.2%)	50.0% (27.2%-72.8%)	60.0% (14.7%-94.7%)
Geometric Mean Fold Rise[3], 95% CI	4.84 (2.20-10.62)	3.22 (1.83-5.67)	3.85 (0.70-21.25)	4.45 (1.04-19.03)	3.23 (1.65-6.29)	3.89 (0.58-25.85)

[1] Antibody values below the lower limit of quantification (LLOQ = 10.254 AU/mL) were assigned a value equal to LLOQ/2. Values greater than the upper limit of quantification (ULOQ = 5740875 AU/mL) are taken as reported, or a ceiling value equivalent to the ULOQ is assigned if values are not provided.

[2] Relative to 4-plex WA-1antibody levels at same visit.

[3] Relative to pre-vaccination (Day 1 Visit) levels, among participants with no missing observations at both pre- and post-boost timepoints.

Supplementary Table 7: **IgG Serum Binding Antibody Response to S-2P-WA-1-** IgG Serum Binding Antibody Response to S-2P-WA-1 Antigen by FFP 10-plex23 ECLIA, by Group and Timepoint

	Group 15E [Dosed Janssen, Boost Novavax] (N=20)	Group 16E [Dosed Moderna, Boost Novavax] (N=16)	Group 17E [Dosed Pfizer, Boost Novavax] (N=31)
**Day 1 Visit (Pre-boost)**			
N (non-missing)	20	16	31
Median (P_{25}, P_{75}), AUC	3271.00 (2821.00-11152.50)	19037.00 (9303.50-40306.50)	11527.00 (7611.00-44583.00)
Minimum - Maximum, AUC	235.30-21940.00	3579.00-67737.00	1294.00-68218.00
Geometric Mean (95% CI), AUC	4123.01 (2352.67-7225.51)	18310.68 (11647.69-28785.21)	14083.75 (9270.68-21395.63)
**Day 15 Visit (14 days post-boost)**			
N (non-missing)	19	15	29
Median (P_{25}, P_{75}), AUC	30774.00 (13551.00-40770.00)	46064.00 (40567.00-65034.00)	44216.00 (39094.00-53637.00)
Minimum - Maximum, AUC	3761.00-53165.00	36840.00-71573.00	22476.00-69847.00
Geometric Mean (95% CI), AUC	22969.71 (15905.34-33171.73)	50130.93 (44100.34-56986.19)	44295.73 (39894.35-49182.71)
N* (non-missing pre- and post-boost)	19	15	29
Participants with 2-fold rise[1], 95% CI	84.2% (60.4%-96.6%)	60.0% (32.3%-83.7%)	62.1% (42.3%-79.3%)
Participants with 4-fold rise[1], 95% CI	57.9% (33.5%-79.7%)	46.7% (21.3%-73.4%)	44.8% (26.4%-64.3%)
Geometric Mean Fold Rise[1], 95% CI	5.19 (3.26-8.26)	2.82 (1.84-4.34)	3.24 (2.19-4.79)
**Day 29 Visit (28 days post-boost)**			
N (non-missing)	18	14	29
Median (P_{25}, P_{75}), AUC	28764.00 (11756.00-35657.00)	47069.00 (40489.00-63157.00)	45201.00 (38254.00-50048.00)
Minimum - Maximum, AUC	2854.00-71573.00	25422.00-69990.00	20338.00-69255.00
Geometric Mean (95% CI), AUC	20950.53 (13923.52-31523.99)	46053.86 (38716.70-54781.47)	42857.80 (38031.14-48297.02)
N* (non-missing pre- and post-boost)	18	14	29
Participants with 2-fold rise[1], 95% CI	83.3% (58.6%-96.4%)	50.0% (23.0%-77.0%)	55.2% (35.7%-73.6%)
Participants with 4-fold rise[1], 95% CI	44.4% (21.5%-69.2%)	42.9% (17.7%-71.1%)	44.8% (26.4%-64.3%)
Geometric Mean Fold Rise[1], 95% CI	4.60 (2.93-7.24)	2.86 (1.75-4.66)	3.13 (2.07-4.74)
**Day 91 Visit (3 months post-boost)**			
N (non-missing)	19	13	25
Median (P_{25}, P_{75}), AUC	16726.00 (8595.00-26665.00)	45519.00 (39828.00-55270.00)	41913.00 (32287.00-58893.00)
Minimum - Maximum, AUC	1271.00-52895.00	22584.00-70459.00	4969.00-67766.00

	Group 15E [Dosed Janssen, Boost Novavax] (N=20)	Group 16E [Dosed Moderna, Boost Novavax] (N=16)	Group 17E [Dosed Pfizer, Boost Novavax] (N=31)
Geometric Mean (95% CI), AUC	15093.62 (9651.71-23603.83)	44220.58 (35889.04-54486.26)	38320.95 (30415.62-48280.96)
N* (non-missing pre- and post-boost)	19	13	25
Participants with 2-fold rise[1], 95% CI	73.7% (48.8%-90.9%)	53.8% (25.1%-80.8%)	60.0% (38.7%-78.9%)
Participants with 4-fold rise[1], 95% CI	31.6% (12.6%-56.6%)	38.5% (13.9%-68.4%)	40.0% (21.1%-61.3%)
Geometric Mean Fold Rise[1], 95% CI	3.41 (2.26-5.14)	2.81 (1.65-4.76)	2.50 (1.66-3.77)

[1] Relative to pre-vaccination (Day 1 Visit) levels, among participants with non-missing observations at both pre- and post-boost timepoints.

Supplementary Table 8: IgG Serum Binding Antibody Response to S-2P-WA-1 by Age - IgG Serum Binding Antibody Response to S-2P-WA-1 Antigen by FFP 10-plex23 ECLIA, by Group, Age Group and Timepoint

	Group 15E [Dosed Janssen, Boost Novavax] Age 18-55 yo (N=11)	Group 15E [Dosed Janssen, Boost Novavax] Age ≥56 yo (N=9)	Group 16E [Dosed Moderna, Boost Novavax] Age 18-55 yo (N=9)	Group 16E [Dosed Moderna, Boost Novavax] Age ≥56 yo (N=7)	Group 17E [Dosed Pfizer, Boost Novavax] Age 18-55 yo (N=25)	Group 17E [Dosed Pfizer, Boost Novavax] Age ≥56 yo (N=6)
**Day 1 Visit (Pre-boost)**						
N (non-missing)	11	9	9	7	25	6
Median (P_{25}, P_{75}), AUC	6503.00 (2647.00-19626.00)	3188.00 (2997.00-3996.00)	20359.00 (8639.00-42268.00)	16846.00 (9968.00-38345.00)	14918.00 (7849.00-46284.00)	6203.50 (1429.00-20513.00)
Minimum - Maximum, AUC	506.00-21940.00	235.30-10141.00	3579.00-67737.00	7872.00-51622.00	3674.00-68218.00	1294.00-44583.00
Geometric Mean (95% CI), AUC	5497.96 (2318.66-13036.65)	2900.36 (1297.44-6483.63)	18107.21 (8596.23-38141.24)	18575.66 (9361.67-36858.27)	17100.49 (11311.55-25852.06)	6273.48 (1415.30-27807.92)
**Day 15 Visit (14 days post-boost)**						
N (non-missing)	10	9	9	6	23	6
Median (P_{25}, P_{75}), AUC	34017.00 (26497.00-40364.00)	22421.00 (13551.00-40770.00)	46064.00 (45586.00-54738.00)	55146.50 (40567.00-65533.00)	44478.00 (39094.00-56975.00)	39671.50 (36260.00-48903.00)
Minimum - Maximum, AUC	3761.00-53165.00	5457.00-44122.00	37695.00-71043.00	36840.00-71573.00	22476.00-69847.00	35551.00-51035.00
Geometric Mean (95% CI), AUC	25972.22 (14830.25-45485.17)	20038.89 (11170.06-35949.41)	48691.01 (41694.41-56861.69)	52371.04 (38848.79-70600.04)	45072.89 (39602.06-51299.49)	41438.75 (35319.01-48618.85)
N* (non-missing pre- and post-boost)	10	9	9	6	23	6
Participants with 2-fold rise[1], 95% CI	80.0% (44.4%-97.5%)	88.9% (51.8%-99.7%)	55.6% (21.2%-86.3%)	66.7% (22.3%-95.7%)	56.5% (34.5%-76.8%)	83.3% (35.9%-99.6%)
Participants with 4-fold rise[1], 95% CI	50.0% (18.7%-81.3%)	66.7% (29.9%-92.5%)	33.3% (7.5%-70.1%)	66.7% (22.3%-95.7%)	39.1% (19.7%-61.5%)	66.7% (22.3%-95.7%)
Geometric Mean Fold Rise[1], 95% CI	4.01 (2.45-6.58)	6.91 (2.80-17.03)	2.69 (1.40-5.15)	3.04 (1.42-6.50)	2.69 (1.82-3.97)	6.61 (1.74-25.09)
**Day 29 Visit (28 days post-boost)**						
N (non-missing)	9	9	8	6	23	6
Median (P_{25}, P_{75}), AUC	29755.00 (22237.00-43002.00)	17167.00 (11756.00-34848.00)	48640.00 (40182.00-56589.50)	44190.50 (40489.00-64239.00)	45819.00 (35985.00-57307.00)	42418.50 (40775.00-43987.00)
Minimum - Maximum, AUC	2854.00-71573.00	6873.00-39232.00	27831.00-63785.00	25422.00-69990.00	20338.00-69255.00	28178.00-50048.00
Geometric Mean (95% CI), AUC	24244.75 (11530.01-50980.67)	18103.92 (10800.38-30346.31)	46376.82 (36995.60-58136.91)	45626.73 (31144.98-66842.20)	43437.75 (37486.55-50333.75)	40705.46 (33204.78-49900.47)
N* (non-missing pre- and post-boost)	9	9	8	6	23	6
Participants with 2-fold rise[1], 95% CI	66.7% (29.9%-92.5%)	100.0% (66.4%-100.0%)	50.0% (15.7%-84.3%)	50.0% (11.8%-88.2%)	47.8% (26.8%-69.4%)	83.3% (35.9%-99.6%)
Participants with 4-fold rise[1], 95% CI	44.4% (13.7%-78.8%)	44.4% (13.7%-78.8%)	37.5% (8.5%-75.5%)	50.0% (11.8%-88.2%)	39.1% (19.7%-61.5%)	66.7% (22.3%-95.7%)
Geometric Mean Fold Rise[1], 95% CI	3.39 (2.10-5.48)	6.24 (2.72-14.30)	3.02 (1.40-6.53)	2.65 (1.12-6.25)	2.59 (1.70-3.94)	6.49 (1.67-25.15)

	Group 15E [Dosed Janssen, Boost Novavax] Age 18-55 yo (N=11)	Group 15E [Dosed Janssen, Boost Novavax] Age ≥56 yo (N=9)	Group 16E [Dosed Moderna, Boost Novavax] Age 18-55 yo (N=9)	Group 16E [Dosed Moderna, Boost Novavax] Age ≥56 yo (N=7)	Group 17E [Dosed Pfizer, Boost Novavax] Age 18-55 yo (N=25)	Group 17E [Dosed Pfizer, Boost Novavax] Age ≥56 yo (N=6)
**Day 91 Visit (3 months post-boost)**						
N (non-missing)	10	9	7	6	20	5
Median (P_{25}, P_{75}), AUC	26328.00 (16257.00-49139.00)	12962.00 (7329.00-16726.00)	45519.00 (43078.00-50832.00)	47549.00 (30665.00-63690.00)	45382.00 (33246.00-61918.00)	39043.00 (25284.00-41913.00)
Minimum - Maximum, AUC	1271.00-52895.00	4530.00-28381.00	22584.00-62518.00	26095.00-70459.00	17562.00-67766.00	4969.00-51535.00
Geometric Mean (95% CI), AUC	19868.42 (8980.01-43959.24)	11121.41 (7054.79-17532.18)	43870.84 (32677.57-58898.21)	44632.13 (29160.60-68312.29)	42465.86 (35407.91-50930.69)	25411.00 (7826.67-82502.36)
N* (non-missing pre- and post-boost)	10	9	7	6	20	5
Participants with 2-fold rise[1], 95% CI	70.0% (34.8%-93.3%)	77.8% (40.0%-97.2%)	57.1% (18.4%-90.1%)	50.0% (11.8%-88.2%)	55.0% (31.5%-76.9%)	80.0% (28.4%-99.5%)
Participants with 4-fold rise[1], 95% CI	30.0% (6.7%-65.2%)	33.3% (7.5%-70.1%)	42.9% (9.9%-81.6%)	33.3% (4.3%-77.7%)	40.0% (19.1%-63.9%)	40.0% (5.3%-85.3%)
Geometric Mean Fold Rise[1], 95% CI	3.07 (1.66-5.69)	3.83 (1.95-7.53)	3.00 (1.15-7.85)	2.59 (1.21-5.56)	2.25 (1.42-3.55)	3.81 (1.01-14.37)

[1] Relative to pre-vaccination (Day 1 Visit) levels, among participants with non-missing observations at both pre- and post-boost timepoints.

Supplementary Table 9: IgG Serum Binding Antibody Response to S-2P-B.351- IgG Serum Binding Antibody Response to S-2P-B.1.351 Antigen by FFP 10-plex23 ECLIA, by Group and Timepoint

	Group 15E [Dosed Janssen, Boost Novavax] (N=20)	Group 16E [Dosed Moderna, Boost Novavax] (N=16)	Group 17E [Dosed Pfizer, Boost Novavax] (N=31)
**Day 1 Visit (Pre-boost)**			
N (non-missing)	20	16	31
Median (P_{25}, P_{75}), AUC	1246.00 (978.95-2537.50)	6305.50 (3465.50-23164.00)	5577.00 (2518.00-34067.00)
Minimum - Maximum, AUC	147.00-13739.00	1692.00-57278.00	596.30-57037.00
Geometric Mean (95% CI), AUC	1490.38 (878.63-2528.05)	8121.67 (4535.28-14544.10)	7695.73 (4702.81-12593.37)
GM Fold Decrease Relative to WA-1[1], 95% CI	2.77 (2.27-3.37)	2.25 (1.84-2.76)	1.83 (1.65-2.03)
**Day 15 Visit (14 days post-boost)**			
N (non-missing)	19	15	29
Median (P_{25}, P_{75}), AUC	16276.00 (6376.00-27066.00)	34298.00 (25819.00-45268.00)	33480.00 (22212.00-38510.00)
Minimum - Maximum, AUC	1067.00-35657.00	18632.00-62933.00	11970.00-61300.00
Geometric Mean (95% CI), AUC	12221.52 (7549.79-19784.05)	35359.99 (29312.99-42654.43)	29907.22 (25349.54-35284.35)
GM Fold Decrease Relative to WA-1[1], 95% CI	1.88 (1.62-2.18)	1.42 (1.31-1.53)	1.48 (1.38-1.59)
N* (non-missing pre- and post-boost)	19	15	29
Participants with 2-fold rise[2], 95% CI	89.5% (66.9%-98.7%)	66.7% (38.4%-88.2%)	62.1% (42.3%-79.3%)
Participants with 4-fold rise[2], 95% CI	84.2% (60.4%-96.6%)	53.3% (26.6%-78.7%)	55.2% (35.7%-73.6%)
Geometric Mean Fold Rise[2], 95% CI	8.01 (4.91-13.07)	4.28 (2.48-7.39)	4.03 (2.62-6.21)
**Day 29 Visit (28 days post-boost)**			
N (non-missing)	18	14	29
Median (P_{25}, P_{75}), AUC	13504.00 (6068.00-18833.00)	32911.50 (25837.00-41306.00)	31477.00 (22620.00-36772.00)
Minimum - Maximum, AUC	1199.00-62933.00	9070.00-51168.00	8870.00-59841.00
Geometric Mean (95% CI), AUC	10675.96 (6334.38-17993.24)	30089.61 (22836.67-39646.08)	28612.56 (23973.56-34149.24)
GM Fold Decrease Relative to WA-1[1], 95% CI	1.96 (1.66-2.33)	1.53 (1.35-1.73)	1.50 (1.40-1.60)
N* (non-missing pre- and post-boost)	18	14	29
Participants with 2-fold rise[2], 95% CI	88.9% (65.3%-98.6%)	64.3% (35.1%-87.2%)	55.2% (35.7%-73.6%)
Participants with 4-fold rise[2], 95% CI	77.8% (52.4%-93.6%)	50.0% (23.0%-77.0%)	51.7% (32.5%-70.6%)
Geometric Mean Fold Rise[2], 95% CI	6.81 (4.18-11.10)	4.19 (2.27-7.74)	3.86 (2.42-6.16)
**Day 91 Visit (3 months post-boost)**			
N (non-missing)	19	13	25

	Group 15E [Dosed Janssen, Boost Novavax] (N=20)	Group 16E [Dosed Moderna, Boost Novavax] (N=16)	Group 17E [Dosed Pfizer, Boost Novavax] (N=31)
Median (P_{25}, P_{75}), AUC	8443.00 (3700.00-13249.00)	32961.00 (18798.00-38726.00)	25251.00 (18754.00-40991.00)
Minimum - Maximum, AUC	505.50-35532.00	10960.00-57166.00	2041.00-56520.00
Geometric Mean (95% CI), AUC	6723.73 (3990.92-11327.86)	26478.42 (19068.38-36768.01)	24620.12 (18379.44-32979.81)
GM Fold Decrease Relative to WA-1[1], 95% CI	2.24 (1.93-2.62)	1.67 (1.46-1.91)	1.56 (1.45-1.67)
N* (non-missing pre- and post-boost)	19	13	25
Participants with 2-fold rise[2], 95% CI	78.9% (54.4%-93.9%)	61.5% (31.6%-86.1%)	56.0% (34.9%-75.6%)
Participants with 4-fold rise[2], 95% CI	42.1% (20.3%-66.5%)	46.2% (19.2%-74.9%)	48.0% (27.8%-68.7%)
Geometric Mean Fold Rise[2], 95% CI	4.41 (2.73-7.12)	3.84 (1.97-7.47)	2.92 (1.79-4.77)

[1] Relative to 10-plex23 WA-1 antibody levels at same visit.

[2] Relative to pre-vaccination (Day 1 Visit) levels, among participants with non-missing observations at both pre- and post-boost timepoints.

Supplementary Table 10: IgG Serum Binding Antibody Response to S-2P-B.351 by Age - IgG Serum Binding Antibody Response to S-2P-B.1.351 Antigen by FFP 10-plex23 ECLIA, by Group, Age Group and Timepoint

	Group 15E [Dosed Janssen, Boost Novavax] Age 18-55 yo (N=11)	Group 15E [Dosed Janssen, Boost Novavax] Age ≥56 yo (N=9)	Group 16E [Dosed Moderna, Boost Novavax] Age 18-55 yo (N=9)	Group 16E [Dosed Moderna, Boost Novavax] Age ≥56 yo (N=7)	Group 17E [Dosed Pfizer, Boost Novavax] Age 18-55 yo (N=25)	Group 17E [Dosed Pfizer, Boost Novavax] Age ≥56 yo (N=6)
**Day 1 Visit (Pre-boost)**						
N (non-missing)	11	9	9	7	25	6
Median (P_{25}, P_{75}), AUC	1952.00 (954.90-6027.00)	1093.00 (1003.00-1712.00)	9883.00 (2722.00-24483.00)	4652.00 (4209.00-21845.00)	8139.00 (4399.00-34642.00)	2297.00 (759.00-7870.00)
Minimum - Maximum, AUC	161.30-13739.00	147.00-2919.00	1692.00-57278.00	2603.00-30807.00	1318.00-57037.00	596.30-31868.00
Geometric Mean (95% CI), AUC	1980.93 (849.93-4616.95)	1052.60 (529.91-2090.86)	8935.99 (3398.70-23494.80)	7182.77 (3063.57-16840.52)	9727.86 (5875.00-16107.43)	2898.81 (603.87-13915.44)
GM Fold Decrease Relative to WA-1[1], 95% CI	2.78 (2.01-3.84)	2.76 (2.08-3.65)	2.03 (1.54-2.67)	2.59 (1.82-3.68)	1.76 (1.57-1.96)	2.16 (1.60-2.93)
**Day 15 Visit (14 days post-boost)**						
N (non-missing)	10	9	9	6	23	6
Median (P_{25}, P_{75}), AUC	17938.50 (12727.00-24575.00)	12192.00 (5262.00-27066.00)	33302.00 (31961.00-39322.00)	37799.50 (25819.00-50451.00)	34544.00 (24048.00-40742.00)	23359.00 (20695.00-34778.00)
Minimum - Maximum, AUC	1679.00-35657.00	1067.00-32763.00	24845.00-59697.00	18632.00-62933.00	11970.00-61300.00	16214.00-35799.00
Geometric Mean (95% CI), AUC	14289.58 (7521.03-27149.47)	10272.76 (4309.65-24486.80)	34963.25 (28269.29-43242.29)	35963.57 (22538.18-57386.10)	31446.15 (25849.21-38254.97)	24674.14 (17759.41-34281.14)
GM Fold Decrease Relative to WA-1[1], 95% CI	1.82 (1.60-2.07)	1.95 (1.42-2.68)	1.39 (1.29-1.50)	1.46 (1.19-1.79)	1.43 (1.32-1.55)	1.68 (1.39-2.02)
N* (non-missing pre- and post-boost)	10	9	9	6	23	6
Participants with 2-fold rise[2], 95% CI	90.0% (55.5%-99.7%)	88.9% (51.8%-99.7%)	66.7% (29.9%-92.5%)	66.7% (22.3%-95.7%)	56.5% (34.5%-76.8%)	83.3% (35.9%-99.6%)
Participants with 4-fold rise[2], 95% CI	80.0% (44.4%-97.5%)	88.9% (51.8%-99.7%)	44.4% (13.7%-78.8%)	66.7% (22.3%-95.7%)	47.8% (26.8%-69.4%)	83.3% (35.9%-99.6%)
Geometric Mean Fold Rise[2], 95% CI	6.71 (3.73-12.07)	9.76 (3.83-24.88)	3.91 (1.72-8.91)	4.91 (1.87-12.90)	3.32 (2.11-5.23)	8.51 (2.37-30.54)
**Day 29 Visit (28 days post-boost)**						
N (non-missing)	9	9	8	6	23	6
Median (P_{25}, P_{75}), AUC	16425.00 (8323.00-30799.00)	9471.00 (4961.00-18068.00)	33850.00 (29039.50-40691.50)	28043.50 (20654.00-49974.00)	34209.00 (21668.00-41842.00)	25192.50 (22620.00-30538.00)
Minimum - Maximum, AUC	1316.00-62933.00	1199.00-23032.00	16767.00-49969.00	9070.00-51168.00	8870.00-59841.00	13852.00-36271.00
Geometric Mean (95% CI), AUC	13426.25 (5578.06-32316.63)	8489.05 (4065.44-17725.99)	32751.21 (24775.28-43294.83)	26874.19 (13719.65-52641.45)	29782.01 (24074.69-36842.35)	24539.58 (17313.29-34781.99)
GM Fold Decrease Relative to WA-1[1], 95% CI	1.81 (1.45-2.24)	2.13 (1.58-2.88)	1.42 (1.30-1.54)	1.70 (1.26-2.29)	1.46 (1.35-1.57)	1.66 (1.39-1.98)
N* (non-missing pre- and post-boost)	9	9	8	6	23	6
Participants with 2-fold rise[2], 95% CI	88.9% (51.8%-99.7%)	88.9% (51.8%-99.7%)	62.5% (24.5%-91.5%)	66.7% (22.3%-95.7%)	47.8% (26.8%-69.4%)	83.3% (35.9%-99.6%)

	Group 15E [Dosed Janssen, Boost Novavax] Age 18-55 yo (N=11)	Group 15E [Dosed Janssen, Boost Novavax] Age ≥56 yo (N=9)	Group 16E [Dosed Moderna, Boost Novavax] Age 18-55 yo (N=9)	Group 16E [Dosed Moderna, Boost Novavax] Age ≥56 yo (N=7)	Group 17E [Dosed Pfizer, Boost Novavax] Age 18-55 yo (N=25)	Group 17E [Dosed Pfizer, Boost Novavax] Age ≥56 yo (N=6)
Participants with 4-fold rise[2], 95% CI	66.7% (29.9%-92.5%)	88.9% (51.8%-99.7%)	50.0% (15.7%-84.3%)	50.0% (11.8%-88.2%)	43.5% (23.2%-65.5%)	83.3% (35.9%-99.6%)
Geometric Mean Fold Rise[2], 95% CI	5.75 (2.86-11.56)	8.06 (3.53-18.44)	4.62 (1.69-12.62)	3.67 (1.37-9.84)	3.14 (1.91-5.16)	8.47 (2.13-33.64)
**Day 91 Visit (3 months post-boost)**						
N (non-missing)	10	9	7	6	20	5
Median (P_{25}, P_{75}), AUC	10731.50 (5657.00-27389.00)	4447.00 (3062.00-8443.00)	32961.00 (23071.00-38726.00)	26973.00 (13633.00-43862.00)	31057.00 (19269.50-42753.00)	25082.00 (12518.00-25251.00)
Minimum - Maximum, AUC	505.50-35532.00	1193.00-15546.00	10960.00-44556.00	11292.00-57166.00	9936.00-56520.00	2041.00-37480.00
Geometric Mean (95% CI), AUC	9261.74 (3768.53-22762.12)	4710.58 (2613.92-8489.01)	27642.08 (17921.65-42634.75)	25182.57 (12560.84-50487.22)	28182.00 (22254.89-35687.67)	14340.48 (3399.79-60488.79)
GM Fold Decrease Relative to WA-1[1], 95% CI	2.15 (1.81-2.54)	2.36 (1.74-3.21)	1.59 (1.34-1.87)	1.77 (1.35-2.33)	1.51 (1.41-1.61)	1.77 (1.34-2.35)
N* (non-missing pre- and post-boost)	10	9	7	6	20	5
Participants with 2-fold rise[2], 95% CI	80.0% (44.4%-97.5%)	77.8% (40.0%-97.2%)	57.1% (18.4%-90.1%)	66.7% (22.3%-95.7%)	50.0% (27.2%-72.8%)	80.0% (28.4%-99.5%)
Participants with 4-fold rise[2], 95% CI	40.0% (12.2%-73.8%)	44.4% (13.7%-78.8%)	57.1% (18.4%-90.1%)	33.3% (4.3%-77.7%)	45.0% (23.1%-68.5%)	60.0% (14.7%-94.7%)
Geometric Mean Fold Rise[2], 95% CI	4.35 (1.92-9.84)	4.48 (2.29-8.76)	4.22 (1.24-14.35)	3.44 (1.36-8.70)	2.61 (1.50-4.51)	4.63 (0.97-22.03)

[1] Relative to 10-plex23 WA-1 antibody levels at same visit.

[2] Relative to pre-vaccination (Day 1 Visit) levels, among participants with non-missing observations at both pre- and post-boost timepoints.

Supplementary Table 6: IgG Serum Binding Antibody Response to S-2P-B.1.617.2- IgG Serum Binding Antibody Response to S-2P-B.1.617.2 Antigen by FFP 10-plex23 ECLIA, by Group and Timepoint

	Group 15E [Dosed Janssen, Boost Novavax] (N=20)	Group 16E [Dosed Moderna, Boost Novavax] (N=16)	Group 17E [Dosed Pfizer, Boost Novavax] (N=31)
**Day 1 Visit (Pre-boost)**			
N (non-missing)	20	16	31
Median (P_{25}, P_{75}), AUC	2400.00 (1491.00-4816.00)	12498.00 (5431.50-29211.50)	9422.00 (3577.00-39540.00)
Minimum - Maximum, AUC	241.60-17709.00	2395.00-60118.00	881.50-64927.00
Geometric Mean (95% CI), AUC	2558.69 (1563.82-4186.46)	11872.12 (7062.10-19958.26)	10428.93 (6576.87-16537.14)
GM Fold Decrease Relative to WA-1[1], 95% CI	1.61 (1.34-1.93)	1.54 (1.34-1.78)	1.35 (1.25-1.46)
**Day 15 Visit (14 days post-boost)**			
N (non-missing)	19	15	29
Median (P_{25}, P_{75}), AUC	22357.00 (8876.00-34134.00)	40587.00 (35080.00-53074.00)	40156.00 (27209.00-45152.00)
Minimum - Maximum, AUC	2261.00-46636.00	21700.00-66024.00	14707.00-67140.00
Geometric Mean (95% CI), AUC	17186.74 (11268.72-26212.74)	41302.86 (35166.30-48510.25)	35909.49 (30979.84-41623.57)
GM Fold Decrease Relative to WA-1[1], 95% CI	1.34 (1.24-1.44)	1.21 (1.14-1.30)	1.23 (1.17-1.30)
N* (non-missing pre- and post-boost)	19	15	29
Participants with 2-fold rise[2], 95% CI	100.0% (82.4%-100.0%)	60.0% (32.3%-83.7%)	62.1% (42.3%-79.3%)
Participants with 4-fold rise[2], 95% CI	68.4% (43.4%-87.4%)	53.3% (26.6%-78.7%)	51.7% (32.5%-70.6%)
Geometric Mean Fold Rise[2], 95% CI	6.39 (4.28-9.53)	3.47 (2.14-5.64)	3.57 (2.38-5.36)
**Day 29 Visit (28 days post-boost)**			
N (non-missing)	18	14	29
Median (P_{25}, P_{75}), AUC	17951.00 (8030.00-24231.00)	37578.50 (32865.00-44624.00)	39025.00 (30425.00-45426.00)
Minimum - Maximum, AUC	1505.00-66024.00	20879.00-63306.00	13282.00-66506.00
Geometric Mean (95% CI), AUC	14825.08 (9202.62-23882.63)	37407.77 (31604.59-44276.52)	34856.19 (29860.57-40687.57)
GM Fold Decrease Relative to WA-1[1], 95% CI	1.41 (1.27-1.58)	1.23 (1.17-1.29)	1.23 (1.17-1.29)
N* (non-missing pre- and post-boost)	18	14	29
Participants with 2-fold rise[2], 95% CI	94.4% (72.7%-99.9%)	57.1% (28.9%-82.3%)	58.6% (38.9%-76.5%)
Participants with 4-fold rise[2], 95% CI	61.1% (35.7%-82.7%)	50.0% (23.0%-77.0%)	48.3% (29.4%-67.5%)
Geometric Mean Fold Rise[2], 95% CI	5.33 (3.59-7.90)	3.53 (2.01-6.19)	3.46 (2.25-5.34)

	Group 15E [Dosed Janssen, Boost Novavax] (N=20)	Group 16E [Dosed Moderna, Boost Novavax] (N=16)	Group 17E [Dosed Pfizer, Boost Novavax] (N=31)
**Day 91 Visit (3 months post-boost)**			
N (non-missing)	19	13	25
Median (P_{25}, P_{75}), AUC	9435.00 (5099.00-20660.00)	37200.00 (25455.00-43552.00)	33231.00 (24504.00-46365.00)
Minimum - Maximum, AUC	562.40-41921.00	16585.00-65734.00	3169.00-62540.00
Geometric Mean (95% CI), AUC	9451.27 (5664.54-15769.44)	32383.00 (25355.82-41357.72)	29771.29 (22790.30-38890.64)
GM Fold Decrease Relative to WA-1[1], 95% CI	1.60 (1.43-1.78)	1.37 (1.27-1.47)	1.29 (1.21-1.36)
N* (non-missing pre- and post-boost)	19	13	25
Participants with 2-fold rise[2], 95% CI	78.9% (54.4%-93.9%)	53.8% (25.1%-80.8%)	56.0% (34.9%-75.6%)
Participants with 4-fold rise[2], 95% CI	31.6% (12.6%-56.6%)	46.2% (19.2%-74.9%)	36.0% (18.0%-57.5%)
Geometric Mean Fold Rise[2], 95% CI	3.51 (2.37-5.21)	3.25 (1.77-5.95)	2.59 (1.67-4.00)

[1] Relative to 10-plex23 WA-1 antibody levels at same visit.
[2] Relative to pre-vaccination (Day 1 Visit) levels, among participants with non-missing observations at both pre- and post-boost timepoints.

Supplementary Table 12: IgG Serum Binding Antibody Response to S-2P-B.1.617.2 by Age- IgG Serum Binding Antibody Response to S-2P-B.1.617.2 Antigen by FFP 10-plex23 ECLIA, by Group, Age Group and Timepoint

	Group 15E [Dosed Janssen, Boost Novavax] Age 18-55 yo (N=11)	Group 15E [Dosed Janssen, Boost Novavax] Age ≥56 yo (N=9)	Group 16E [Dosed Moderna, Boost Novavax] Age 18-55 yo (N=9)	Group 16E [Dosed Moderna, Boost Novavax] Age ≥56 yo (N=7)	Group 17E [Dosed Pfizer, Boost Novavax] Age 18-55 yo (N=25)	Group 17E [Dosed Pfizer, Boost Novavax] Age ≥56 yo (N=6)
**Day 1 Visit (Pre-boost)**						
N (non-missing)	11	9	9	7	25	6
Median (P_{25}, P_{75}), AUC	3523.00 (1474.00-11260.00)	2023.00 (1508.00-3074.00)	13399.00 (3895.00-28506.00)	11597.00 (6282.00-29917.00)	9529.00 (6156.00-43499.00)	3048.50 (990.00-12481.00)
Minimum - Maximum, AUC	241.60-17709.00	480.30-5010.00	2395.00-60118.00	4581.00-36857.00	1991.00-64927.00	881.50-39540.00
Geometric Mean (95% CI), AUC	3176.87 (1351.61-7467.05)	1964.03 (1164.33-3312.99)	11857.34 (4933.38-28499.02)	11891.15 (5672.84-24925.66)	13150.93 (8324.59-20775.41)	3968.15 (837.21-18807.91)
GM Fold Decrease Relative to WA-1[1], 95% CI	1.73 (1.41-2.13)	1.48 (1.03-2.12)	1.53 (1.21-1.93)	1.56 (1.25-1.96)	1.30 (1.20-1.41)	1.58 (1.23-2.03)
**Day 15 Visit (14 days post-boost)**						
N (non-missing)	10	9	9	6	23	6
Median (P_{25}, P_{75}), AUC	25465.50 (17551.00-31109.00)	18166.00 (8876.00-34134.00)	40275.00 (36316.00-44121.00)	46925.50 (32657.00-56023.00)	40378.00 (27738.00-47795.00)	30113.50 (25719.00-42417.00)
Minimum - Maximum, AUC	2261.00-46636.00	2957.00-42735.00	31207.00-63299.00	21700.00-66024.00	14707.00-67140.00	23479.00-43315.00
Geometric Mean (95% CI), AUC	19197.76 (10207.71-36105.44)	15198.37 (7589.17-30436.86)	40695.33 (34783.40-47612.06)	42231.21 (27450.69-64970.14)	37129.58 (31059.23-44386.36)	31592.53 (23968.78-41641.17)
GM Fold Decrease Relative to WA-1[1], 95% CI	1.35 (1.22-1.51)	1.32 (1.15-1.51)	1.20 (1.12-1.28)	1.24 (1.05-1.47)	1.21 (1.14-1.30)	1.31 (1.14-1.50)
N* (non-missing pre- and post-boost)	10	9	9	6	23	6
Participants with 2-fold rise[2], 95% CI	100.0% (69.2%-100.0%)	100.0% (66.4%-100.0%)	55.6% (21.2%-86.3%)	66.7% (22.3%-95.7%)	56.5% (34.5%-76.8%)	83.3% (35.9%-99.6%)
Participants with 4-fold rise[2], 95% CI	70.0% (34.8%-93.3%)	66.7% (29.9%-92.5%)	44.4% (13.7%-78.8%)	66.7% (22.3%-95.7%)	47.8% (26.8%-69.4%)	66.7% (22.3%-95.7%)
Geometric Mean Fold Rise[2], 95% CI	5.37 (3.31-8.72)	7.74 (3.64-16.44)	3.43 (1.59-7.42)	3.54 (1.64-7.61)	2.89 (1.93-4.34)	7.96 (2.16-29.28)
**Day 29 Visit (28 days post-boost)**						
N (non-missing)	9	9	8	6	23	6
Median (P_{25}, P_{75}), AUC	23267.00 (11699.00-38722.00)	12918.00 (7560.00-22004.00)	37576.00 (34037.00-43400.50)	37584.50 (28693.00-54747.00)	39819.00 (26165.00-47390.00)	33997.00 (30425.00-39025.00)
Minimum - Maximum, AUC	1505.00-66024.00	3084.00-35683.00	25016.00-46569.00	20879.00-63306.00	13282.00-66506.00	18410.00-42155.00
Geometric Mean (95% CI), AUC	17366.73 (7250.65-41596.71)	12655.40 (6956.12-23024.18)	37091.15 (31424.26-43779.99)	37834.14 (24661.67-58042.41)	35658.43 (29560.45-43014.36)	31944.62 (23440.04-43534.85)
GM Fold Decrease Relative to WA-1[1], 95% CI	1.40 (1.17-1.67)	1.43 (1.20-1.70)	1.25 (1.17-1.34)	1.21 (1.10-1.32)	1.22 (1.15-1.29)	1.27 (1.12-1.45)

	Group 15E [Dosed Janssen, Boost Novavax] Age 18-55 yo (N=11)	Group 15E [Dosed Janssen, Boost Novavax] Age ≥56 yo (N=9)	Group 16E [Dosed Moderna, Boost Novavax] Age 18-55 yo (N=9)	Group 16E [Dosed Moderna, Boost Novavax] Age ≥56 yo (N=7)	Group 17E [Dosed Pfizer, Boost Novavax] Age 18-55 yo (N=25)	Group 17E [Dosed Pfizer, Boost Novavax] Age ≥56 yo (N=6)
N* (non-missing pre- and post-boost)	9	9	8	6	23	6
Participants with 2-fold rise[2], 95% CI	88.9% (51.8%-99.7%)	100.0% (66.4%-100.0%)	62.5% (24.5%-91.5%)	50.0% (11.8%-88.2%)	52.2% (30.6%-73.2%)	83.3% (35.9%-99.6%)
Participants with 4-fold rise[2], 95% CI	66.7% (29.9%-92.5%)	55.6% (21.2%-86.3%)	50.0% (15.7%-84.3%)	50.0% (11.8%-88.2%)	43.5% (23.2%-65.5%)	66.7% (22.3%-95.7%)
Geometric Mean Fold Rise[2], 95% CI	4.41 (2.57-7.56)	6.44 (3.29-12.61)	3.83 (1.57-9.37)	3.17 (1.22-8.25)	2.78 (1.79-4.31)	8.05 (2.07-31.28)
**Day 91 Visit (3 months post-boost)**						
N (non-missing)	10	9	7	6	20	5
Median (P_{25}, P_{75}), AUC	16662.00 (7664.00-38040.00)	7562.00 (3823.00-9435.00)	37200.00 (27132.00-38643.00)	34503.50 (20998.00-48560.00)	34360.50 (24708.50-47933.50)	28769.00 (15149.00-37125.00)
Minimum - Maximum, AUC	562.40-41921.00	2795.00-21385.00	16585.00-43775.00	18742.00-65734.00	11664.00-62540.00	3169.00-43167.00
Geometric Mean (95% CI), AUC	12616.35 (4983.67-31938.74)	6856.60 (4162.85-11293.45)	31521.81 (23176.30-42872.45)	33417.50 (19601.96-56970.28)	33496.12 (26939.12-41649.10)	18578.47 (4941.19-69853.51)
GM Fold Decrease Relative to WA-1[1], 95% CI	1.57 (1.33-1.86)	1.62 (1.35-1.94)	1.39 (1.27-1.53)	1.34 (1.13-1.57)	1.27 (1.19-1.35)	1.37 (1.11-1.69)
N* (non-missing pre- and post-boost)	10	9	7	6	20	5
Participants with 2-fold rise[2], 95% CI	80.0% (44.4%-97.5%)	77.8% (40.0%-97.2%)	57.1% (18.4%-90.1%)	50.0% (11.8%-88.2%)	50.0% (27.2%-72.8%)	80.0% (28.4%-99.5%)
Participants with 4-fold rise[2], 95% CI	30.0% (6.7%-65.2%)	33.3% (7.5%-70.1%)	57.1% (18.4%-90.1%)	33.3% (4.3%-77.7%)	35.0% (15.4%-59.2%)	40.0% (5.3%-85.3%)
Geometric Mean Fold Rise[2], 95% CI	3.53 (1.78-7.00)	3.49 (2.06-5.92)	3.68 (1.25-10.86)	2.80 (1.15-6.82)	2.28 (1.40-3.70)	4.29 (1.10-16.68)

[1] Relative to 10-plex23 WA-1 antibody levels at same visit.

[2] Relative to pre-vaccination (Day 1 Visit) levels, among participants with non-missing observations at both pre- and post-boost timepoints.

Supplementary Table 13: IgG Serum Binding Antibody Response to S-2P-B.1.1.529- IgG Serum Binding Antibody Response to S-2P-B.1.1.529 Antigen by FFP 10-plex23 ECLIA, by Group and Timepoint

	Group 15E [Dosed Janssen, Boost Novavax] (N=20)	Group 16E [Dosed Moderna, Boost Novavax] (N=16)	Group 17E [Dosed Pfizer, Boost Novavax] (N=31)
**Day 1 Visit (Pre-boost)**			
N (non-missing)	20	16	31
Median (P_{25}, P_{75}), AUC	505.90 (295.50-1257.80)	2756.00 (1332.50-10678.00)	2445.00 (1065.00-18667.00)
Minimum - Maximum, AUC	26.24-7013.00	633.80-43689.00	231.50-49084.00
Geometric Mean (95% CI), AUC	535.39 (276.96-1034.96)	3511.12 (1750.16-7043.90)	3269.64 (1828.82-5845.59)
GM Fold Decrease Relative to WA-1[1], 95% CI	7.70 (5.30-11.19)	5.22 (3.83-7.10)	4.31 (3.51-5.29)
**Day 15 Visit (14 days post-boost)**			
N (non-missing)	19	15	29
Median (P_{25}, P_{75}), AUC	6737.00 (2777.00-14229.00)	20325.00 (12188.00-32309.00)	17103.00 (10486.00-24687.00)
Minimum - Maximum, AUC	384.10-29243.00	9490.00-55783.00	4839.00-51077.00
Geometric Mean (95% CI), AUC	5968.57 (3459.42-10297.64)	21524.83 (15803.89-29316.71)	16656.77 (13114.36-21156.05)
GM Fold Decrease Relative to WA-1[1], 95% CI	3.85 (3.13-4.74)	2.33 (1.93-2.81)	2.66 (2.31-3.06)
N* (non-missing pre- and post-boost)	19	15	29
Participants with 2-fold rise[2], 95% CI	94.7% (74.0%-99.9%)	73.3% (44.9%-92.2%)	65.5% (45.7%-82.1%)
Participants with 4-fold rise[2], 95% CI	84.2% (60.4%-96.6%)	60.0% (32.3%-83.7%)	58.6% (38.9%-76.5%)
Geometric Mean Fold Rise[2], 95% CI	11.84 (6.64-21.12)	5.99 (3.14-11.41)	5.36 (3.29-8.73)
**Day 29 Visit (28 days post-boost)**			
N (non-missing)	18	14	29
Median (P_{25}, P_{75}), AUC	6829.50 (2516.00-9502.00)	16882.00 (12354.00-31095.00)	16053.00 (8983.00-23687.00)
Minimum - Maximum, AUC	465.70-55783.00	3341.00-41839.00	3493.00-49088.00
Geometric Mean (95% CI), AUC	5164.32 (2788.59-9564.03)	17202.81 (11290.70-26210.67)	15484.34 (11969.45-20031.40)
GM Fold Decrease Relative to WA-1[1], 95% CI	4.06 (3.15-5.22)	2.68 (2.07-3.45)	2.77 (2.39-3.20)
N* (non-missing pre- and post-boost)	18	14	29
Participants with 2-fold rise[2], 95% CI	94.4% (72.7%-99.9%)	71.4% (41.9%-91.6%)	62.1% (42.3%-79.3%)
Participants with 4-fold rise[2], 95% CI	88.9% (65.3%-98.6%)	50.0% (23.0%-77.0%)	51.7% (32.5%-70.6%)
Geometric Mean Fold Rise[2], 95% CI	9.97 (5.39-18.44)	5.72 (2.82-11.61)	4.98 (2.93-8.47)
**Day 91 Visit (3 months post-boost)**			

	Group 15E [Dosed Janssen, Boost Novavax] (N=20)	Group 16E [Dosed Moderna, Boost Novavax] (N=16)	Group 17E [Dosed Pfizer, Boost Novavax] (N=31)
N (non-missing)	19	13	25
Median (P_{25}, P_{75}), AUC	3443.00 (1416.00-6761.00)	16540.00 (9054.00-26872.00)	14942.00 (7406.00-23486.00)
Minimum - Maximum, AUC	240.90-25942.00	4135.00-55757.00	855.60-52984.00
Geometric Mean (95% CI), AUC	3184.43 (1790.03-5665.05)	14911.93 (8938.59-24877.04)	13089.37 (8831.07-19401.00)
GM Fold Decrease Relative to WA-1[1], 95% CI	4.74 (3.83-5.87)	2.97 (2.16-4.06)	2.93 (2.42-3.54)
N* (non-missing pre- and post-boost)	19	13	25
Participants with 2-fold rise[2], 95% CI	84.2% (60.4%-96.6%)	61.5% (31.6%-86.1%)	56.0% (34.9%-75.6%)
Participants with 4-fold rise[2], 95% CI	68.4% (43.4%-87.4%)	46.2% (19.2%-74.9%)	52.0% (31.3%-72.2%)
Geometric Mean Fold Rise[2], 95% CI	6.32 (3.58-11.14)	5.09 (2.24-11.56)	3.51 (1.93-6.36)

[1] Relative to 10-plex23 WA-1 antibody levels at same visit.

[2] Relative to pre-vaccination (Day 1 Visit) levels, among participants with non-missing observations at both pre- and post-boost timepoints.

Supplementary Table 14: IgG Serum Binding Antibody Response to S-2P-B.1.1.529 by Age- IgG Serum Binding Antibody Response to S-2P-B.1.1.529 Antigen by FFP 10-plex23 ECLIA, by Group, Age Group and Timepoint

	Group 15E [Dosed Janssen, Boost Novavax] Age 18-55 yo (N=11)	Group 15E [Dosed Janssen, Boost Novavax] Age ≥56 yo (N=9)	Group 16E [Dosed Moderna, Boost Novavax] Age 18-55 yo (N=9)	Group 16E [Dosed Moderna, Boost Novavax] Age ≥56 yo (N=7)	Group 17E [Dosed Pfizer, Boost Novavax] Age 18-55 yo (N=25)	Group 17E [Dosed Pfizer, Boost Novavax] Age ≥56 yo (N=6)
**Day 1 Visit (Pre-boost)**						
N (non-missing)	11	9	9	7	25	6
Median (P_{25}, P_{75}), AUC	676.80 (309.60-3610.00)	378.40 (230.80-570.40)	3637.00 (1038.00-12958.00)	1732.00 (1627.00-8398.00)	2734.00 (1372.00-20408.00)	1084.00 (274.40-3267.00)
Minimum - Maximum, AUC	50.74-7013.00	26.24-1547.00	633.80-43689.00	934.20-22772.00	535.80-49084.00	231.50-14472.00
Geometric Mean (95% CI), AUC	822.28 (307.11-2201.65)	316.89 (125.12-802.58)	3989.56 (1271.99-12513.09)	2979.31 (1053.60-8424.71)	4131.22 (2197.85-7765.28)	1233.83 (241.06-6315.09)
GM Fold Decrease Relative to WA-1[1], 95% CI	6.69 (3.46-12.91)	9.15 (6.20-13.51)	4.54 (2.89-7.12)	6.23 (3.71-10.48)	4.14 (3.23-5.30)	5.08 (3.64-7.10)
**Day 15 Visit (14 days post-boost)**						
N (non-missing)	10	9	9	6	23	6
Median (P_{25}, P_{75}), AUC	8661.00 (6034.00-12547.00)	6737.00 (2626.00-14293.00)	20325.00 (18369.00-28140.00)	21643.00 (11857.00-41356.00)	18369.00 (10095.00-27419.00)	12288.00 (10486.00-17103.00)
Minimum - Maximum, AUC	692.70-29243.00	384.10-18152.00	10432.00-49047.00	9490.00-55783.00	4839.00-51077.00	6884.00-21073.00
Geometric Mean (95% CI), AUC	7094.75 (3380.93-14888.07)	4925.65 (1866.07-13001.63)	21274.95 (14777.34-30629.55)	21905.16 (10343.73-46389.08)	17936.82 (13464.59-23894.49)	12540.97 (8289.56-18972.78)
GM Fold Decrease Relative to WA-1[1], 95% CI	3.66 (2.89-4.64)	4.07 (2.70-6.14)	2.29 (1.84-2.84)	2.39 (1.51-3.80)	2.51 (2.13-2.96)	3.30 (2.50-4.37)
N* (non-missing pre- and post-boost)	10	9	9	6	23	6
Participants with 2-fold rise[2], 95% CI	90.0% (55.5%-99.7%)	100.0% (66.4%-100.0%)	77.8% (40.0%-97.2%)	66.7% (22.3%-95.7%)	60.9% (38.5%-80.3%)	83.3% (35.9%-99.6%)
Participants with 4-fold rise[2], 95% CI	80.0% (44.4%-97.5%)	88.9% (51.8%-99.7%)	55.6% (21.2%-86.3%)	66.7% (22.3%-95.7%)	52.2% (30.6%-73.2%)	83.3% (35.9%-99.6%)
Geometric Mean Fold Rise[2], 95% CI	9.26 (4.93-17.42)	15.54 (4.96-48.74)	5.33 (2.11-13.45)	7.12 (2.04-24.86)	4.54 (2.63-7.83)	10.16 (2.74-37.67)
**Day 29 Visit (28 days post-boost)**						
N (non-missing)	9	9	8	6	23	6
Median (P_{25}, P_{75}), AUC	8992.00 (3366.00-14532.00)	4426.00 (2123.00-8850.00)	20716.00 (13798.50-29956.00)	13516.00 (12328.00-37646.00)	17521.00 (8514.00-31050.00)	13241.00 (8983.00-14205.00)
Minimum - Maximum, AUC	481.50-55783.00	465.70-11252.00	6040.00-38898.00	3341.00-41839.00	3493.00-49088.00	7041.00-21670.00
Geometric Mean (95% CI), AUC	6907.95 (2358.77-20230.78)	3860.80 (1712.42-8704.50)	18988.08 (11518.98-31300.26)	15080.79 (5743.59-39597.19)	16453.48 (12010.47-22540.07)	12269.52 (8142.53-18488.26)
GM Fold Decrease Relative to WA-1[1], 95% CI	3.51 (2.32-5.30)	4.69 (3.29-6.68)	2.44 (1.84-3.24)	3.03 (1.68-5.45)	2.64 (2.22-3.14)	3.32 (2.59-4.25)
N* (non-missing pre- and post-boost)	9	9	8	6	23	6
Participants with 2-fold rise[2], 95% CI	88.9% (51.8%-99.7%)	100.0% (66.4%-100.0%)	75.0% (34.9%-96.8%)	66.7% (22.3%-95.7%)	56.5% (34.5%-76.8%)	83.3% (35.9%-99.6%)

	Group 15E [Dosed Janssen, Boost Novavax] Age 18-55 yo (N=11)	Group 15E [Dosed Janssen, Boost Novavax] Age ≥56 yo (N=9)	Group 16E [Dosed Moderna, Boost Novavax] Age 18-55 yo (N=9)	Group 16E [Dosed Moderna, Boost Novavax] Age ≥56 yo (N=7)	Group 17E [Dosed Pfizer, Boost Novavax] Age 18-55 yo (N=25)	Group 17E [Dosed Pfizer, Boost Novavax] Age ≥56 yo (N=6)
Participants with 4-fold rise[2], 95% CI	77.8% (40.0%-97.2%)	100.0% (66.4%-100.0%)	50.0% (15.7%-84.3%)	50.0% (11.8%-88.2%)	43.5% (23.2%-65.5%)	83.3% (35.9%-99.6%)
Geometric Mean Fold Rise[2], 95% CI	8.16 (3.25-20.47)	12.18 (4.44-33.46)	6.42 (2.10-19.66)	4.90 (1.45-16.62)	4.16 (2.29-7.56)	9.94 (2.46-40.17)
**Day 91 Visit (3 months post-boost)**						
N (non-missing)	10	9	7	6	20	5
Median (P_{25}, P_{75}), AUC	4749.00 (2431.00-19368.00)	2172.00 (1416.00-3883.00)	16540.00 (11431.00-26872.00)	12839.00 (5617.00-36424.00)	20329.50 (8271.50-30480.00)	9425.00 (4358.00-14942.00)
Minimum - Maximum, AUC	240.90-25942.00	593.80-6983.00	4245.00-40648.00	4135.00-55757.00	3626.00-52984.00	855.60-20067.00
Geometric Mean (95% CI), AUC	4579.31 (1662.51-12613.53)	2126.83 (1167.59-3874.15)	15873.44 (8092.77-31134.69)	13863.53 (4675.16-41110.33)	15667.94 (10766.75-22800.23)	6375.97 (1330.36-30557.78)
GM Fold Decrease Relative to WA-1[1], 95% CI	4.34 (3.04-6.19)	5.23 (3.90-7.00)	2.76 (1.86-4.11)	3.22 (1.64-6.33)	2.71 (2.20-3.35)	3.99 (2.40-6.62)
N* (non-missing pre- and post-boost)	10	9	7	6	20	5
Participants with 2-fold rise[2], 95% CI	80.0% (44.4%-97.5%)	88.9% (51.8%-99.7%)	57.1% (18.4%-90.1%)	66.7% (22.3%-95.7%)	50.0% (27.2%-72.8%)	80.0% (28.4%-99.5%)
Participants with 4-fold rise[2], 95% CI	70.0% (34.8%-93.3%)	66.7% (29.9%-92.5%)	57.1% (18.4%-90.1%)	33.3% (4.3%-77.7%)	50.0% (27.2%-72.8%)	60.0% (14.7%-94.7%)
Geometric Mean Fold Rise[2], 95% CI	5.98 (2.36-15.12)	6.71 (2.87-15.71)	5.66 (1.30-24.61)	4.51 (1.33-15.27)	3.20 (1.60-6.41)	5.05 (0.95-26.86)

[1] Relative to 10-plex23 WA-1 antibody levels at same visit.

[2] Relative to pre-vaccination (Day 1 Visit) levels, among participants with non-missing observations at both pre- and post-boost timepoints.

Supplementary Table 15: Neutralization Antibodies Titer (ID50) to Pseudovirus D614G[1], by Group and Timepoint

	Group 15E Dosed Janssen Boost Novavax (N=20)	Group 16E Dosed Moderna Boost Novavax (N=16)	Group 17E Dosed Pfizer/BioNTech Boost Novavax (N=31)
**Day 1 Visit (Pre-boost)**			
N (non-missing)	20	16	31
Positive Response(%)[2]	18 (90.0%)	16 (100.0%)	30 (96.8%)
Median (P_{25}, P_{75})	76.66 (27.48-118.96)	188.91 (87.86-2218.82)	133.13 (36.89-3070.66)
Minimum - Maximum	5.00-776.25	31.62-5751.18	5.00-129232.64
Geometric Mean (95% CI)	64.48 (33.65-123.55)	347.37 (137.96-874.60)	355.30 (141.98-889.15)
**Day 15 Visit (14 days post-boost)**			
N (non-missing)	19	15	29
Positive Response(%)[2]	19 (100.0%)	15 (100.0%)	29 (100.0%)
Median (P_{25}, P_{75})	620.09 (230.55-1672.64)	2944.92 (1076.54-5570.08)	2873.23 (1007.94-5571.81)
Minimum - Maximum	72.99-11561.58	471.13-9930.65	167.56-45887.51
Geometric Mean (95% CI)	619.78 (342.68-1120.97)	2431.34 (1407.84-4198.95)	2839.93 (1786.26-4515.13)
N* (non-missing pre- and post-boost)	19	15	29
Participants with ≥ 2-fold rise[3], 95% CI	100.0% (82.4%-100.0%)	80.0% (51.9%-95.7%)	65.5% (45.7%-82.1%)
Participants with ≥ 4-fold rise[3], 95% CI	68.4% (43.4%-87.4%)	66.7% (38.4%-88.2%)	55.2% (35.7%-73.6%)
Geometric Mean Fold Rise[3], 95% CI	8.40 (5.21-13.56)	6.51 (3.33-12.72)	8.30 (4.22-16.31)
**Day 29 Visit (28 days post-boost)**			
N (non-missing)	18	14	29
Positive Response(%)[2]	18 (100.0%)	14 (100.0%)	29 (100.0%)
Median (P_{25}, P_{75})	625.95 (206.84-974.52)	1737.93 (792.90-5620.49)	2505.32 (1256.18-4179.07)
Minimum - Maximum	39.94-7383.85	410.84-14003.20	232.23-55446.28
Geometric Mean (95% CI)	499.25 (269.68-924.25)	1978.27 (1024.75-3819.03)	2681.75 (1762.56-4080.31)
N* (non-missing pre- and post-boost)	18	14	29
Participants with ≥ 2-fold rise[3], 95% CI	88.9% (65.3%-98.6%)	64.3% (35.1%-87.2%)	58.6% (38.9%-76.5%)
Participants with ≥ 4-fold rise[3], 95% CI	72.2% (46.5%-90.3%)	50.0% (23.0%-77.0%)	55.2% (35.7%-73.6%)
Geometric Mean Fold Rise[3], 95% CI	6.43 (4.17-9.89)	6.44 (2.36-17.55)	7.83 (3.86-15.92)
**Day 91 Visit (3 months post-boost)**			
N (non-missing)	19	13	25

	Group 15E<br>Dosed Janssen<br>Boost Novavax<br>(N=20)	Group 16E<br>Dosed Moderna<br>Boost Novavax<br>(N=16)	Group 17E<br>Dosed Pfizer/BioNTech<br>Boost Novavax<br>(N=31)
Positive Response(%)[2]	19 (100.0%)	13 (100.0%)	25 (100.0%)
Median (P_{25}, P_{75})	212.85 (136.60-723.32)	1452.82 (588.71-3610.51)	2073.13 (820.04-3280.30)
Minimum - Maximum	29.57-4577.55	304.91-23688.86	74.24-70003.68
Geometric Mean (95% CI)	305.52 (149.47-624.50)	1661.38 (776.81-3553.24)	1741.71 (949.23-3195.82)
N* (non-missing pre- and post-boost)	19	13	25
Participants with ≥ 2-fold rise[3], 95% CI	84.2% (60.4%-96.6%)	61.5% (31.6%-86.1%)	56.0% (34.9%-75.6%)
Participants with ≥ 4-fold rise[3], 95% CI	52.6% (28.9%-75.6%)	46.2% (19.2%-74.9%)	52.0% (31.3%-72.2%)
Geometric Mean Fold Rise[3], 95% CI	4.14 (2.58-6.65)	5.68 (1.89-17.02)	3.90 (1.82-8.35)

[1] Values below the lower limit of detection (LLOD = 10) were assigned the value of LLOD/2. Values between the LLOD and the lower limit of quantification (LLOQ = 18.5) are taken as reported, or a value of LLOQ/2 is assigned if observations are reported as <LLOQ but no value is provided. Values greater than the upper limit of quantification (ULOQ = 45118) are taken as reported, or a ceiling value equivalent to the ULOQ is assigned if values are not provided.

[2] Positive response is defined as an ID titer above the LLOD.

[3] Relative to pre-vaccination (Day 1 Visit) levels, among participants with non-missing observations at both pre- and post-boost timepoints.

Supplementary Table 16: Neutralization Antibodies Titer (ID50) to Pseudovirus D614G[1], by Group, Age Group and Timepoint

	Group 15E Dosed Janssen Boost Novavax Age 18-55 yo (N=11)	Group 15E Dosed Janssen Boost Novavax Age ≥56 yo (N=9)	Group 16E Dosed Moderna Boost Novavax Age 18-55 yo (N=9)	Group 16E Dosed Moderna Boost Novavax Age ≥56 yo (N=7)	Group 17E Dosed Pfizer/BioNTech Boost Novavax Age 18-55 yo (N=25)	Group 17E Dosed Pfizer/BioNTech Boost Novavax Age ≥56 yo (N=6)
**Day 1 Visit (Pre-boost)**						
N (non-missing)	11	9	9	7	25	6
Positive Response(%)[2]	10 (90.9%)	8 (88.9%)	9 (100.0%)	7 (100.0%)	25 (100.0%)	5 (83.3%)
Median (P_{25}, P_{75})	110.15 (28.92-408.48)	36.64 (27.31-60.31)	203.88 (89.26-3671.88)	117.30 (83.80-825.97)	372.92 (65.02-3070.66)	53.16 (21.90-166.30)
Minimum - Maximum	5.00-776.25	5.00-94.97	31.62-5751.18	55.92-3611.67	27.84-129232.64	5.00-4268.05
Geometric Mean (95% CI)	109.32 (39.21-304.81)	33.82 (16.84-67.91)	420.56 (96.11-1840.30)	271.66 (63.60-1160.42)	514.85 (187.72-1412.02)	75.76 (6.77-847.55)
**Day 15 Visit (14 days post-boost)**						
N (non-missing)	10	9	9	6	23	6
Positive Response(%)[2]	10 (100.0%)	9 (100.0%)	9 (100.0%)	6 (100.0%)	23 (100.0%)	6 (100.0%)
Median (P_{25}, P_{75})	773.20 (581.46-1858.14)	301.01 (185.04-1217.85)	2944.92 (1093.39-3955.75)	2474.73 (837.17-5570.08)	3521.30 (951.44-6954.82)	1397.10 (1021.07-3097.88)
Minimum - Maximum	72.99-11561.58	139.77-1941.32	1056.64-9846.83	471.13-9930.65	167.56-45887.51	904.63-5063.46
Geometric Mean (95% CI)	838.91 (318.11-2212.34)	442.75 (198.30-988.55)	2608.89 (1293.60-5261.55)	2187.41 (640.20-7473.87)	3228.37 (1836.71-5674.48)	1737.34 (845.97-3567.91)
N* (non-missing pre- and post-boost)	10	9	9	6	23	6
Participants with ≥ 2-fold rise[3], 95% CI	100.0% (69.2%-100.0%)	100.0% (66.4%-100.0%)	77.8% (40.0%-97.2%)	83.3% (35.9%-99.6%)	60.9% (38.5%-80.3%)	83.3% (35.9%-99.6%)
Participants with ≥4-fold rise[3], 95% CI	50.0% (18.7%-81.3%)	88.9% (51.8%-99.7%)	66.7% (29.9%-92.5%)	66.7% (22.3%-95.7%)	47.8% (26.8%-69.4%)	83.3% (35.9%-99.6%)
Geometric Mean Fold Rise[3], 95% CI	5.64 (2.90-10.94)	13.09 (6.50-26.36)	6.20 (2.59-14.87)	7.00 (1.58-31.04)	6.36 (3.03-13.37)	22.93 (3.58-146.78)
**Day 29 Visit (28 days post-boost)**						
N (non-missing)	9	9	8	6	23	6
Positive Response(%)[2]	9 (100.0%)	9 (100.0%)	8 (100.0%)	6 (100.0%)	23 (100.0%)	6 (100.0%)
Median (P_{25}, P_{75})	754.01 (580.98-884.35)	231.11 (183.85-974.52)	1905.61 (1190.54-4788.12)	914.64 (775.44-12025.41)	2505.32 (1257.36-5662.21)	2033.59 (1196.23-3121.77)
Minimum - Maximum	39.94-7383.85	121.47-1190.00	545.35-5678.16	410.84-14003.20	232.23-55446.28	752.27-3803.83
Geometric Mean (95% CI)	710.19 (228.16-2210.63)	350.97 (176.66-697.27)	2052.36 (997.12-4224.34)	1883.62 (380.16-9333.12)	2962.28 (1774.85-4944.16)	1831.42 (925.11-3625.59)
N* (non-missing pre- and post-boost)	9	9	8	6	23	6
Participants with ≥ 2-fold rise[3], 95% CI	77.8% (40.0%-97.2%)	100.0% (66.4%-100.0%)	62.5% (24.5%-91.5%)	66.7% (22.3%-95.7%)	52.2% (30.6%-73.2%)	83.3% (35.9%-99.6%)
Participants with ≥4-fold rise[3], 95% CI	55.6% (21.2%-86.3%)	88.9% (51.8%-99.7%)	50.0% (15.7%-84.3%)	50.0% (11.8%-88.2%)	47.8% (26.8%-69.4%)	83.3% (35.9%-99.6%)
Geometric Mean Fold Rise[3], 95% CI	3.98 (2.30-6.87)	10.38 (5.79-18.61)	6.77 (1.62-28.31)	6.03 (0.79-45.76)	5.84 (2.71-12.59)	24.18 (3.25-179.76)

	Group 15E Dosed Janssen Boost Novavax Age 18-55 yo (N=11)	Group 15E Dosed Janssen Boost Novavax Age ≥56 yo (N=9)	Group 16E Dosed Moderna Boost Novavax Age 18-55 yo (N=9)	Group 16E Dosed Moderna Boost Novavax Age ≥56 yo (N=7)	Group 17E Dosed Pfizer/BioNTech Boost Novavax Age 18-55 yo (N=25)	Group 17E Dosed Pfizer/BioNTech Boost Novavax Age ≥56 yo (N=6)
**Day 91 Visit (3 months post-boost)**						
N (non-missing)	10	9	7	6	20	5
Positive Response(%)[2]	10 (100.0%)	9 (100.0%)	7 (100.0%)	6 (100.0%)	20 (100.0%)	5 (100.0%)
Median (P_{25}, P_{75})	603.17 (180.11-2311.50)	153.26 (136.60-212.85)	1288.05 (588.71-3708.20)	1929.53 (453.13-3364.99)	2297.43 (1010.23-3608.43)	750.21 (185.18-1826.10)
Minimum - Maximum	29.57-4577.55	34.28-512.38	316.35-5426.56	304.91-23688.86	74.24-70003.68	136.12-3116.55
Geometric Mean (95% CI)	607.05 (189.66-1943.07)	142.47 (73.85-274.86)	1523.87 (575.07-4038.06)	1837.58 (356.33-9476.44)	2236.80 (1153.22-4338.51)	640.29 (116.09-3531.39)
N* (non-missing pre- and post-boost)	10	9	7	6	20	5
Participants with ≥ 2-fold rise[3], 95% CI	70.0% (34.8%-93.3%)	100.0% (66.4%-100.0%)	57.1% (18.4%-90.1%)	66.7% (22.3%-95.7%)	50.0% (27.2%-72.8%)	80.0% (28.4%-99.5%)
Participants with ≥4-fold rise[3], 95% CI	50.0% (18.7%-81.3%)	55.6% (21.2%-86.3%)	57.1% (18.4%-90.1%)	33.3% (4.3%-77.7%)	45.0% (23.1%-68.5%)	80.0% (28.4%-99.5%)
Geometric Mean Fold Rise[3], 95% CI	4.08 (1.57-10.59)	4.21 (3.07-5.78)	5.50 (1.22-24.83)	5.88 (0.60-57.79)	3.35 (1.36-8.27)	7.17 (1.21-42.64)

[1] Values below the lower limit of detection (LLOD = 10) were assigned the value of LLOD/2. Values between the LLOD and the lower limit of quantification (LLOQ = 18.5) are taken as reported, or a value of LLOQ/2 is assigned if observations are reported as <LLOQ but no value is provided. Values greater than the upper limit of quantification (ULOQ = 45118) are taken as reported, or a ceiling value equivalent to the ULOQ is assigned if values are not provided.

[2] Positive response is defined as an ID titer above the LLOD.

[3] Relative to pre-vaccination (Day 1 Visit) levels, among participants with non-missing observations at both pre- and post-boost timepoints.

Supplementary Table 17: Neutralization Antibodies Titer (ID50) to Pseudovirus B.1.351[1], by Group and Timepoint

	Group 15E Dosed Janssen Boost Novavax (N=20)	Group 16E Dosed Moderna Boost Novavax (N=16)	Group 17E Dosed Pfizer/BioNTech Boost Novavax (N=31)
**Day 1 Visit (Pre-boost)**			
N (non-missing)	20	16	31
Positive Response(%)[2]	10 (50.0%)	15 (93.8%)	24 (77.4%)
Median (P_{25}, P_{75})	7.64 (5.00-35.33)	30.71 (16.66-422.18)	31.93 (10.46-853.71)
Minimum - Maximum	5.00-131.02	5.00-3956.67	5.00-41823.74
Geometric Mean (95% CI)	14.74 (8.12-26.74)	69.95 (24.37-200.76)	88.62 (34.93-224.84)
GM Fold Decrease Relative to D614G[3], 95% CI	5.64 (4.08-7.80)	4.97 (3.56-6.93)	4.20 (3.47-5.08)
**Day 29 Visit (28 days post-boost)**			
N (non-missing)	18	14	29
Positive Response(%)[2]	17 (94.4%)	14 (100.0%)	29 (100.0%)
Median (P_{25}, P_{75})	133.18 (29.14-411.37)	641.93 (204.11-1300.66)	845.19 (586.05-1582.19)
Minimum - Maximum	5.00-1685.43	91.15-5031.11	87.51-64832.15
Geometric Mean (95% CI)	112.57 (52.02-243.59)	568.68 (291.85-1108.11)	1040.55 (631.42-1714.78)
GM Fold Decrease Relative to D614G[3], 95% CI	4.44 (3.00-6.55)	3.48 (2.69-4.49)	2.58 (2.07-3.21)
N* (non-missing pre- and post-boost)	18	14	29
Participants with ≥ 2-fold rise[4], 95% CI	83.3% (58.6%-96.4%)	78.6% (49.2%-95.3%)	79.3% (60.3%-92.0%)
Participants with ≥ 4-fold rise[4], 95% CI	61.1% (35.7%-82.7%)	64.3% (35.1%-87.2%)	58.6% (38.9%-76.5%)
Geometric Mean Fold Rise[4], 95% CI	6.77 (3.64-12.59)	9.75 (3.80-24.98)	12.30 (6.30-24.03)
**Day 91 Visit (3 months post-boost)**			
N (non-missing)	19	13	25
Positive Response(%)[2]	15 (78.9%)	13 (100.0%)	25 (100.0%)
Median (P_{25}, P_{75})	44.98 (16.92-127.07)	476.73 (102.16-577.91)	379.86 (227.59-731.46)
Minimum - Maximum	5.00-777.52	22.66-2382.32	13.84-58054.02
Geometric Mean (95% CI)	48.11 (21.96-105.44)	291.20 (116.89-725.47)	408.30 (204.60-814.82)
GM Fold Decrease Relative to D614G[3], 95% CI	6.35 (4.86-8.30)	5.71 (3.93-8.28)	4.27 (3.47-5.25)
N* (non-missing pre- and post-boost)	19	13	25
Participants with ≥ 2-fold rise[4], 95% CI	57.9% (33.5%-79.7%)	69.2% (38.6%-90.9%)	60.0% (38.7%-78.9%)

	Group 15E<br>Dosed Janssen<br>Boost Novavax<br>(N=20)	Group 16E<br>Dosed Moderna<br>Boost Novavax<br>(N=16)	Group 17E<br>Dosed Pfizer/BioNTech<br>Boost Novavax<br>(N=31)
Participants with ≥ 4-fold rise[4], 95% CI	47.4% (24.4%-71.1%)	46.2% (19.2%-74.9%)	52.0% (31.3%-72.2%)
Geometric Mean Fold Rise[4], 95% CI	3.08 (1.51-6.28)	5.22 (1.53-17.82)	3.85 (1.71-8.69)

[1] Values below the lower limit of detection (LLOD = 10) were assigned the value of LLOD/2.
[2] Positive response is defined as an ID titer above the LLOD.
[3] Relative to D614G titer at same visit, among participants with positive response to D614G.
[4] Relative to pre-vaccination (Day 1 Visit) levels, among participants with non-missing observations at both pre- and post-boost timepoints.

Supplementary Table 18: Neutralization Antibodies Titer (ID50) to Pseudovirus B.1.351[1], by Group, Age Group and Timepoint

	Group 15E Dosed Janssen Boost Novavax Age 18-55 yo (N=11)	Group 15E Dosed Janssen Boost Novavax Age ≥56 yo (N=9)	Group 16E Dosed Moderna Boost Novavax Age 18-55 yo (N=9)	Group 16E Dosed Moderna Boost Novavax Age ≥56 yo (N=7)	Group 17E Dosed Pfizer/BioNTech Boost Novavax Age 18-55 yo (N=25)	Group 17E Dosed Pfizer/BioNTech Boost Novavax Age ≥56 yo (N=6)
**Day 1 Visit (Pre-boost)**						
N (non-missing)	11	9	9	7	25	6
Positive Response(%)[2]	6 (54.5%)	4 (44.4%)	9 (100.0%)	6 (85.7%)	21 (84.0%)	3 (50.0%)
Median (P_{25}, P_{75})	31.43 (5.00-118.31)	5.00 (5.00-17.26)	33.61 (21.50-935.84)	24.21 (10.63-196.15)	145.48 (12.91-853.71)	18.47 (5.00-37.25)
Minimum - Maximum	5.00-131.02	5.00-25.86	13.11-3956.67	5.00-648.21	5.00-41823.74	5.00-912.53
Geometric Mean (95% CI)	22.19 (8.13-60.57)	8.94 (5.07-15.76)	109.00 (20.85-569.71)	39.55 (8.10-193.17)	122.92 (42.54-355.18)	22.67 (2.66-193.47)
GM Fold Decrease Relative to D614G[3], 95% CI	5.78 (3.24-10.31)	5.48 (3.82-7.86)	3.86 (2.43-6.13)	6.87 (4.23-11.16)	4.19 (3.35-5.23)	4.25 (2.63-6.88)
**Day 29 Visit (28 days post-boost)**						
N (non-missing)	9	9	8	6	23	6
Positive Response(%)[2]	9 (100.0%)	8 (88.9%)	8 (100.0%)	6 (100.0%)	23 (100.0%)	6 (100.0%)
Median (P_{25}, P_{75})	146.80 (128.38-490.92)	36.01 (24.18-175.55)	749.86 (390.42-1220.99)	293.09 (204.11-1300.66)	913.78 (611.79-2712.39)	410.03 (237.06-1055.62)
Minimum - Maximum	14.45-1685.43	5.00-871.35	170.18-2552.55	91.15-5031.11	87.51-64832.15	217.35-1623.97
Geometric Mean (95% CI)	194.40 (69.53-543.52)	65.18 (18.61-228.33)	663.55 (301.34-1461.18)	462.94 (99.95-2144.18)	1267.72 (705.29-2278.66)	488.10 (202.75-1175.05)
GM Fold Decrease Relative to D614G[3], 95% CI	3.65 (2.41-5.54)	5.38 (2.56-11.31)	3.09 (2.20-4.34)	4.07 (2.46-6.73)	2.34 (1.84-2.96)	3.75 (2.01-6.99)
N* (non-missing pre- and post-boost)	9	9	8	6	23	6
Participants with ≥ 2-fold rise[4], 95% CI	88.9% (51.8%-99.7%)	77.8% (40.0%-97.2%)	75.0% (34.9%-96.8%)	83.3% (35.9%-99.6%)	78.3% (56.3%-92.5%)	83.3% (35.9%-99.6%)
Participants with ≥4-fold rise[4], 95% CI	44.4% (13.7%-78.8%)	77.8% (40.0%-97.2%)	62.5% (24.5%-91.5%)	66.7% (22.3%-95.7%)	52.2% (30.6%-73.2%)	83.3% (35.9%-99.6%)
Geometric Mean Fold Rise[4], 95% CI	6.29 (2.78-14.23)	7.29 (2.35-22.58)	9.54 (2.32-39.18)	10.03 (1.68-60.01)	10.63 (4.83-23.40)	21.53 (4.48-103.52)
**Day 91 Visit (3 months post-boost)**						
N (non-missing)	10	9	7	6	20	5
Positive Response(%)[2]	9 (90.0%)	6 (66.7%)	7 (100.0%)	6 (100.0%)	20 (100.0%)	5 (100.0%)
Median (P_{25}, P_{75})	96.78 (35.27-490.60)	21.24 (5.00-70.47)	242.14 (102.16-477.62)	548.08 (26.82-1229.24)	406.11 (299.70-1445.29)	116.11 (48.94-341.01)
Minimum - Maximum	5.00-777.52	5.00-98.42	72.36-2282.37	22.66-2382.32	13.84-58054.02	22.13-508.26
Geometric Mean (95% CI)	100.25 (31.26-321.57)	21.28 (8.17-55.47)	296.85 (102.88-856.55)	284.75 (35.85-2261.92)	558.23 (260.03-1198.43)	116.86 (23.05-592.34)
GM Fold Decrease Relative to D614G[3], 95% CI	6.06 (4.29-8.54)	6.69 (4.04-11.08)	5.13 (3.53-7.47)	6.45 (2.72-15.30)	4.01 (3.15-5.10)	5.48 (3.36-8.92)
N* (non-missing pre- and post-boost)	10	9	7	6	20	5

	Group 15E<br>Dosed Janssen<br>Boost Novavax<br>Age 18-55 yo<br>(N=11)	Group 15E<br>Dosed Janssen<br>Boost Novavax<br>Age ≥56 yo<br>(N=9)	Group 16E<br>Dosed Moderna<br>Boost Novavax<br>Age 18-55 yo<br>(N=9)	Group 16E<br>Dosed Moderna<br>Boost Novavax<br>Age ≥56 yo<br>(N=7)	Group 17E<br>Dosed Pfizer/BioNTech<br>Boost Novavax<br>Age 18-55 yo<br>(N=25)	Group 17E<br>Dosed Pfizer/BioNTech<br>Boost Novavax<br>Age ≥56 yo<br>(N=6)
Participants with ≥ 2-fold rise[4], 95% CI	50.0% (18.7%-81.3%)	66.7% (29.9%-92.5%)	71.4% (29.0%-96.3%)	66.7% (22.3%-95.7%)	55.0% (31.5%-76.9%)	80.0% (28.4%-99.5%)
Participants with ≥4-fold rise[4], 95% CI	50.0% (18.7%-81.3%)	44.4% (13.7%-78.8%)	57.1% (18.4%-90.1%)	33.3% (4.3%-77.7%)	50.0% (27.2%-72.8%)	60.0% (14.7%-94.7%)
Geometric Mean Fold Rise[4], 95% CI	3.89 (1.13-13.44)	2.38 (0.96-5.89)	4.52 (0.70-28.96)	6.17 (0.59-64.60)	3.87 (1.44-10.41)	3.81 (0.66-21.90)

[1] Values below the lower limit of detection (LLOD = 10) were assigned the value of LLOD/2.
[2] Positive response is defined as an ID titer above the LLOD.
[3] Relative to D614G titer at same visit, among participants with positive response to D614G.
[4] Relative to pre-vaccination (Day 1 Visit) levels, among participants with non-missing observations at both pre- and post-boost timepoints.

Supplementary Table 19: Neutralization Antibodies Titer (ID50) to Pseudovirus B.1.1.529 BA.1[1], by Group and Timepoint

	Group 15E Dosed Janssen Boost Novavax (N=20)	Group 16E Dosed Moderna Boost Novavax (N=16)	Group 17E Dosed Pfizer/BioNTech Boost Novavax (N=31)
**Day 1 Visit (Pre-boost)**			
N (non-missing)	20	16	31
Positive Response(%)[2]	10 (50.0%)	12 (75.0%)	21 (67.7%)
Median (P_{25}, P_{75})	8.17 (5.00-30.27)	20.63 (9.20-348.50)	24.33 (5.00-1177.76)
Minimum - Maximum	5.00-208.65	5.00-1952.71	5.00-26001.46
Geometric Mean (95% CI)	13.36 (7.63-23.39)	47.67 (15.32-148.32)	87.47 (30.75-248.82)
GM Fold Decrease Relative to D614G[3], 95% CI	6.42 (4.38-9.41)	7.29 (4.90-10.84)	4.26 (3.09-5.87)
**Day 15 Visit (14 days post-boost)**			
N (non-missing)	19	15	29
Positive Response(%)[2]	19 (100.0%)	15 (100.0%)	29 (100.0%)
Median (P_{25}, P_{75})	156.71 (28.09-257.84)	485.27 (347.03-2943.32)	643.80 (402.67-2195.29)
Minimum - Maximum	11.45-4928.23	49.49-22686.27	20.99-27553.25
Geometric Mean (95% CI)	126.81 (61.79-260.24)	749.46 (323.18-1738.00)	816.04 (472.76-1408.58)
GM Fold Decrease Relative to D614G[3], 95% CI	4.89 (3.67-6.50)	3.24 (1.89-5.57)	3.48 (2.71-4.46)
N* (non-missing pre- and post-boost)	19	15	29
Participants with ≥ 2-fold rise[4], 95% CI	94.7% (74.0%-99.9%)	86.7% (59.5%-98.3%)	69.0% (49.2%-84.7%)
Participants with ≥ 4-fold rise[4], 95% CI	78.9% (54.4%-93.9%)	86.7% (59.5%-98.3%)	55.2% (35.7%-73.6%)
Geometric Mean Fold Rise[4], 95% CI	10.01 (5.76-17.38)	14.59 (7.47-28.50)	9.71 (4.61-20.45)
**Day 29 Visit (28 days post-boost)**			
N (non-missing)	18	14	29
Positive Response(%)[2]	17 (94.4%)	14 (100.0%)	29 (100.0%)
Median (P_{25}, P_{75})	173.84 (34.18-208.46)	641.78 (210.24-1574.14)	1141.57 (260.29-2932.79)
Minimum - Maximum	5.00-4432.10	123.30-9878.85	21.03-38464.75
Geometric Mean (95% CI)	114.81 (51.74-254.75)	704.28 (313.97-1579.80)	1181.12 (639.02-2183.10)
GM Fold Decrease Relative to D614G[3], 95% CI	4.35 (3.27-5.78)	2.81 (1.79-4.42)	2.27 (1.62-3.18)
N* (non-missing pre- and post-boost)	18	14	29
Participants with ≥ 2-fold rise[4], 95% CI	94.4% (72.7%-99.9%)	92.9% (66.1%-99.8%)	75.9% (56.5%-89.7%)

	Group 15E Dosed Janssen Boost Novavax (N=20)	Group 16E Dosed Moderna Boost Novavax (N=16)	Group 17E Dosed Pfizer/BioNTech Boost Novavax (N=31)
Participants with ≥ 4-fold rise[4], 95% CI	83.3% (58.6%-96.4%)	85.7% (57.2%-98.2%)	65.5% (45.7%-82.1%)
Geometric Mean Fold Rise[4], 95% CI	8.60 (5.33-13.88)	17.74 (7.24-43.44)	14.05 (6.55-30.13)
**Day 91 Visit (3 months post-boost)**			
N (non-missing)	19	13	25
Positive Response(%)[2]	18 (94.7%)	13 (100.0%)	24 (96.0%)
Median (P_{25}, P_{75})	68.99 (31.19-154.60)	1026.26 (187.89-2050.07)	765.03 (389.59-1760.52)
Minimum - Maximum	5.00-2933.63	68.20-2683.10	5.00-23147.93
Geometric Mean (95% CI)	82.90 (38.02-180.79)	589.29 (248.62-1396.74)	627.30 (307.69-1278.94)
GM Fold Decrease Relative to D614G[3], 95% CI	3.69 (2.77-4.91)	2.82 (1.95-4.08)	2.78 (2.11-3.66)
N* (non-missing pre- and post-boost)	19	13	25
Participants with ≥ 2-fold rise[4], 95% CI	89.5% (66.9%-98.7%)	92.3% (64.0%-99.8%)	56.0% (34.9%-75.6%)
Participants with ≥ 4-fold rise[4], 95% CI	73.7% (48.8%-90.9%)	69.2% (38.6%-90.9%)	56.0% (34.9%-75.6%)
Geometric Mean Fold Rise[4], 95% CI	6.54 (3.38-12.68)	14.99 (5.04-44.56)	5.80 (2.21-15.25)

[1] Values below the lower limit of detection (LLOD = 10) were assigned the value of LLOD/2.
[2] Positive response is defined as an ID titer above the LLOD.
[3] Relative to D614G titer at same visit, among participants with positive response to D614G.
[4] Relative to pre-vaccination (Day 1 Visit) levels, among participants with non-missing observations at both pre- and post-boost timepoints.

Supplementary Table 20: Neutralization Antibodies Titer (ID50) to Pseudovirus B.1.1.529 BA.1[1], by Group, Age Group and Timepoint

	Group 15E Dosed Janssen Boost Novavax Age 18-55 yo (N=11)	Group 15E Dosed Janssen Boost Novavax Age ≥56 yo (N=9)	Group 16E Dosed Moderna Boost Novavax Age 18-55 yo (N=9)	Group 16E Dosed Moderna Boost Novavax Age ≥56 yo (N=7)	Group 17E Dosed Pfizer/BioNTech Boost Novavax Age 18-55 yo (N=25)	Group 17E Dosed Pfizer/BioNTech Boost Novavax Age ≥56 yo (N=6)
**Day 1 Visit (Pre-boost)**						
N (non-missing)	11	9	9	7	25	6
Positive Response(%)[2]	7 (63.6%)	3 (33.3%)	8 (88.9%)	4 (57.1%)	19 (76.0%)	2 (33.3%)
Median (P_{25}, P_{75})	20.18 (5.00-80.47)	5.00 (5.00-11.35)	21.44 (17.58-559.38)	15.52 (5.00-137.63)	72.56 (10.90-1177.76)	5.00 (5.00-12.04)
Minimum - Maximum	5.00-208.65	5.00-24.18	5.00-1952.71	5.00-765.65	5.00-26001.46	5.00-3052.65
Geometric Mean (95% CI)	21.11 (8.45-52.74)	7.63 (4.58-12.73)	71.18 (12.47-406.15)	28.48 (4.53-178.86)	129.86 (40.59-415.42)	16.86 (1.14-250.40)
GM Fold Decrease Relative to D614G[3], 95% CI	7.44 (4.54-12.21)	5.34 (2.58-11.02)	5.91 (3.07-11.38)	9.54 (5.90-15.43)	3.96 (2.85-5.52)	6.07 (1.48-24.88)
**Day 15 Visit (14 days post-boost)**						
N (non-missing)	10	9	9	6	23	6
Positive Response(%)[2]	10 (100.0%)	9 (100.0%)	9 (100.0%)	6 (100.0%)	23 (100.0%)	6 (100.0%)
Median (P_{25}, P_{75})	187.11 (114.00-364.11)	84.44 (28.09-235.56)	620.15 (385.18-2943.32)	400.45 (147.73-1256.14)	868.55 (441.79-2511.79)	413.15 (190.27-1292.65)
Minimum - Maximum	13.87-4928.23	11.45-573.23	302.86-4415.17	49.49-22686.27	20.99-27553.25	145.46-2152.48
Geometric Mean (95% CI)	183.11 (58.05-577.57)	84.30 (30.51-232.96)	903.64 (395.75-2063.34)	566.09 (61.77-5187.88)	936.61 (488.46-1795.90)	481.17 (157.00-1474.66)
GM Fold Decrease Relative to D614G[3], 95% CI	4.58 (3.01-6.97)	5.25 (3.25-8.49)	2.89 (1.75-4.76)	3.86 (0.90-16.67)	3.45 (2.58-4.60)	3.61 (1.82-7.17)
N* (non-missing pre- and post-boost)	10	9	9	6	23	6
Participants with ≥ 2-fold rise[4], 95% CI	90.0% (55.5%-99.7%)	100.0% (66.4%-100.0%)	77.8% (40.0%-97.2%)	100.0% (54.1%-100.0%)	65.2% (42.7%-83.6%)	83.3% (35.9%-99.6%)
Participants with ≥4-fold rise[4], 95% CI	70.0% (34.8%-93.3%)	88.9% (51.8%-99.7%)	77.8% (40.0%-97.2%)	100.0% (54.1%-100.0%)	47.8% (26.8%-69.4%)	83.3% (35.9%-99.6%)
Geometric Mean Fold Rise[4], 95% CI	9.16 (3.82-21.93)	11.04 (4.67-26.12)	12.70 (4.33-37.24)	17.97 (6.75-47.84)	7.33 (3.24-16.58)	28.54 (3.45-236.08)
**Day 29 Visit (28 days post-boost)**						
N (non-missing)	9	9	8	6	23	6
Positive Response(%)[2]	8 (88.9%)	9 (100.0%)	8 (100.0%)	6 (100.0%)	23 (100.0%)	6 (100.0%)
Median (P_{25}, P_{75})	182.27 (138.00-660.15)	35.12 (34.18-195.05)	905.13 (419.07-1766.33)	356.25 (146.72-798.23)	1141.57 (424.85-6096.85)	807.85 (235.25-1572.46)
Minimum - Maximum	5.00-4432.10	20.44-239.62	185.82-8519.57	123.30-9878.85	21.03-38464.75	197.88-2112.58
Geometric Mean (95% CI)	193.20 (43.61-855.92)	68.22 (30.47-152.75)	914.17 (322.65-2590.13)	497.39 (89.71-2757.68)	1402.24 (671.93-2926.29)	611.80 (192.37-1945.77)
GM Fold Decrease Relative to D614G[3], 95% CI	3.68 (2.23-6.05)	5.14 (3.61-7.34)	2.25 (1.25-4.02)	3.79 (1.54-9.30)	2.11 (1.45-3.08)	2.99 (1.08-8.27)

	Group 15E Dosed Janssen Boost Novavax Age 18-55 yo (N=11)	Group 15E Dosed Janssen Boost Novavax Age ≥56 yo (N=9)	Group 16E Dosed Moderna Boost Novavax Age 18-55 yo (N=9)	Group 16E Dosed Moderna Boost Novavax Age ≥56 yo (N=7)	Group 17E Dosed Pfizer/BioNTech Boost Novavax Age 18-55 yo (N=25)	Group 17E Dosed Pfizer/BioNTech Boost Novavax Age ≥56 yo (N=6)
N* (non-missing pre- and post-boost)	9	9	8	6	23	6
Participants with ≥ 2-fold rise[4], 95% CI	88.9% (51.8%-99.7%)	100.0% (66.4%-100.0%)	100.0% (63.1%-100.0%)	83.3% (35.9%-99.6%)	73.9% (51.6%-89.8%)	83.3% (35.9%-99.6%)
Participants with ≥4-fold rise[4], 95% CI	66.7% (29.9%-92.5%)	100.0% (66.4%-100.0%)	87.5% (47.3%-99.7%)	83.3% (35.9%-99.6%)	60.9% (38.5%-80.3%)	83.3% (35.9%-99.6%)
Geometric Mean Fold Rise[4], 95% CI	8.28 (3.20-21.41)	8.94 (5.38-14.86)	19.36 (4.88-76.84)	15.79 (3.08-80.86)	10.97 (4.71-25.57)	36.29 (4.10-320.94)
**Day 91 Visit (3 months post-boost)**						
N (non-missing)	10	9	7	6	20	5
Positive Response(%)[2]	9 (90.0%)	9 (100.0%)	7 (100.0%)	6 (100.0%)	19 (95.0%)	5 (100.0%)
Median (P_{25}, P_{75})	141.10 (56.84-424.26)	51.57 (20.03-68.99)	681.42 (187.89-2050.07)	1167.75 (71.97-2630.14)	1213.84 (399.27-1896.66)	484.51 (59.04-713.94)
Minimum - Maximum	5.00-2933.63	11.03-98.35	69.28-2350.50	68.20-2683.10	5.00-23147.93	41.14-977.60
Geometric Mean (95% CI)	164.42 (42.62-634.32)	38.74 (20.92-71.74)	580.47 (176.74-1906.40)	599.75 (100.11-3592.92)	796.38 (353.93-1791.95)	241.50 (38.56-1512.25)
GM Fold Decrease Relative to D614G[3], 95% CI	3.69 (2.32-5.88)	3.68 (2.38-5.69)	2.63 (1.93-3.57)	3.06 (1.24-7.58)	2.81 (2.00-3.95)	2.65 (1.62-4.33)
N* (non-missing pre- and post-boost)	10	9	7	6	20	5
Participants with ≥ 2-fold rise[4], 95% CI	80.0% (44.4%-97.5%)	100.0% (66.4%-100.0%)	85.7% (42.1%-99.6%)	100.0% (54.1%-100.0%)	50.0% (27.2%-72.8%)	80.0% (28.4%-99.5%)
Participants with ≥4-fold rise[4], 95% CI	80.0% (44.4%-97.5%)	66.7% (29.9%-92.5%)	57.1% (18.4%-90.1%)	83.3% (35.9%-99.6%)	50.0% (27.2%-72.8%)	80.0% (28.4%-99.5%)
Geometric Mean Fold Rise[4], 95% CI	8.22 (2.27-29.73)	5.07 (3.00-8.60)	12.22 (1.94-76.79)	19.03 (3.16-114.75)	4.92 (1.61-15.01)	11.23 (0.66-191.24)

[1] Values below the lower limit of detection (LLOD = 10) were assigned the value of LLOD/2.

[2] Positive response is defined as an ID titer above the LLOD.

[3] Relative to D614G titer at same visit, among participants with positive response to D614G.

[4] Relative to pre-vaccination (Day 1 Visit) levels, among participants with non-missing observations at both pre- and post-boost timepoints.

Supplementary Table 21: Neutralization Antibodies Titer (ID50) to Pseudovirus B.1.1.529 BA.4/BA.5[1], by Group and Timepoint

	Group 15E Dosed Janssen Boost Novavax (N=20)	Group 16E Dosed Moderna Boost Novavax (N=16)	Group 17E Dosed Pfizer/BioNTech Boost Novavax (N=31)
**Day 1 Visit (Pre-boost)**			
N (non-missing)	20	16	31
Positive Response(%)[2]	9 (45.0%)	12 (75.0%)	23 (74.2%)
Median (P_{25}, P_{75})	5.00 (5.00-19.99)	22.59 (7.82-173.05)	19.76 (5.00-950.12)
Minimum - Maximum	5.00-94.69	5.00-1144.88	5.00-59156.68
Geometric Mean (95% CI)	11.03 (6.89-17.66)	36.92 (14.17-96.20)	75.45 (28.06-202.89)
GM Fold Decrease Relative to D614G[3], 95% CI	7.11 (5.69-8.90)	9.41 (6.68-13.24)	4.96 (3.74-6.58)
**Day 29 Visit (28 days post-boost)**			
N (non-missing)	18	14	29
Positive Response(%)[2]	15 (83.3%)	14 (100.0%)	29 (100.0%)
Median (P_{25}, P_{75})	111.25 (30.51-286.42)	471.18 (167.65-756.71)	773.16 (237.91-1718.11)
Minimum - Maximum	5.00-4977.91	30.58-9330.48	25.18-76962.97
Geometric Mean (95% CI)	93.75 (37.71-233.08)	400.69 (177.53-904.37)	657.35 (349.44-1236.58)
GM Fold Decrease Relative to D614G[3], 95% CI	5.33 (3.49-8.12)	4.94 (2.99-8.15)	4.08 (2.85-5.85)
N* (non-missing pre- and post-boost)	18	14	29
Participants with ≥ 2-fold rise[4], 95% CI	77.8% (52.4%-93.6%)	92.9% (66.1%-99.8%)	75.9% (56.5%-89.7%)
Participants with ≥ 4-fold rise[4], 95% CI	66.7% (41.0%-86.7%)	64.3% (35.1%-87.2%)	65.5% (45.7%-82.1%)
Geometric Mean Fold Rise[4], 95% CI	7.78 (3.84-15.79)	11.34 (4.68-27.47)	9.16 (4.86-17.24)
**Day 91 Visit (3 months post-boost)**			
N (non-missing)	19	13	25
Positive Response(%)[2]	16 (84.2%)	13 (100.0%)	22 (88.0%)
Median (P_{25}, P_{75})	60.41 (20.92-206.28)	871.50 (147.92-2313.95)	531.13 (210.49-1830.62)
Minimum - Maximum	5.00-2956.65	14.79-3424.10	5.00-34376.26
Geometric Mean (95% CI)	69.70 (27.55-176.36)	415.50 (135.37-1275.32)	449.53 (187.85-1075.73)
GM Fold Decrease Relative to D614G[3], 95% CI	4.38 (2.97-6.46)	4.00 (2.11-7.59)	3.87 (2.64-5.68)
N* (non-missing pre- and post-boost)	19	13	25
Participants with ≥ 2-fold rise[4], 95% CI	73.7% (48.8%-90.9%)	84.6% (54.6%-98.1%)	52.0% (31.3%-72.2%)

	Group 15E Dosed Janssen Boost Novavax (N=20)	Group 16E Dosed Moderna Boost Novavax (N=16)	Group 17E Dosed Pfizer/BioNTech Boost Novavax (N=31)
Participants with ≥ 4-fold rise[4], 95% CI	57.9% (33.5%-79.7%)	61.5% (31.6%-86.1%)	48.0% (27.8%-68.7%)
Geometric Mean Fold Rise[4], 95% CI	6.06 (2.80-13.12)	12.73 (3.73-43.45)	5.05 (2.09-12.18)

[1] Values below the lower limit of detection (LLOD = 10) were assigned the value of LLOD/2.
[2] Positive response is defined as an ID titer above the LLOD.
[3] Relative to D614G titer at same visit, among participants with positive response to D614G.
[4] Relative to pre-vaccination (Day 1 Visit) levels, among participants with non-missing observations at both pre- and post-boost timepoints.

Supplementary Table 22: Neutralization Antibodies Titer (ID50) to Pseudovirus B.1.1.529 BA.4/BA.5[1], by Group, Age Group and Timepoint

	Group 15E Dosed Janssen Boost Novavax Age 18-55 yo (N=11)	Group 15E Dosed Janssen Boost Novavax Age ≥56 yo (N=9)	Group 16E Dosed Moderna Boost Novavax Age 18-55 yo (N=9)	Group 16E Dosed Moderna Boost Novavax Age ≥56 yo (N=7)	Group 17E Dosed Pfizer/BioNTech Boost Novavax Age 18-55 yo (N=25)	Group 17E Dosed Pfizer/BioNTech Boost Novavax Age ≥56 yo (N=6)
**Day 1 Visit (Pre-boost)**						
N (non-missing)	11	9	9	7	25	6
Positive Response(%)[2]	7 (63.6%)	2 (22.2%)	8 (88.9%)	4 (57.1%)	20 (80.0%)	3 (50.0%)
Median (P_{25}, P_{75})	18.32 (5.00-49.87)	5.00 (5.00-5.00)	28.44 (11.32-209.85)	14.17 (5.00-136.24)	23.13 (12.77-950.12)	8.76 (5.00-15.86)
Minimum - Maximum	5.00-94.69	5.00-18.20	5.00-1144.88	5.00-226.54	5.00-59156.68	5.00-2161.44
Geometric Mean (95% CI)	16.84 (7.95-35.68)	6.58 (4.33-10.00)	53.26 (12.31-230.50)	23.04 (4.94-107.49)	104.50 (34.12-320.09)	19.42 (1.62-232.31)
GM Fold Decrease Relative to D614G[3], 95% CI	7.83 (5.44-11.27)	6.31 (4.68-8.51)	7.90 (4.67-13.35)	11.79 (7.10-19.59)	4.93 (3.57-6.80)	5.12 (2.17-12.11)
**Day 29 Visit (28 days post-boost)**						
N (non-missing)	9	9	8	6	23	6
Positive Response(%)[2]	8 (88.9%)	7 (77.8%)	8 (100.0%)	6 (100.0%)	23 (100.0%)	6 (100.0%)
Median (P_{25}, P_{75})	144.18 (97.25-178.19)	71.88 (26.53-286.42)	572.45 (348.26-773.23)	311.02 (93.74-487.98)	800.43 (319.64-2468.09)	144.58 (73.18-820.32)
Minimum - Maximum	5.00-4977.91	5.00-764.74	74.65-1742.21	30.58-9330.48	31.16-76962.97	25.18-1460.60
Geometric Mean (95% CI)	145.78 (35.60-597.00)	60.29 (14.85-244.88)	478.89 (218.96-1047.40)	315.91 (40.59-2458.60)	915.70 (467.43-1793.84)	184.49 (36.65-928.74)
GM Fold Decrease Relative to D614G[3], 95% CI	4.87 (3.32-7.16)	5.82 (2.46-13.78)	4.29 (2.39-7.68)	5.96 (1.88-18.87)	3.24 (2.34-4.47)	9.93 (2.89-34.06)
N* (non-missing pre- and post-boost)	9	9	8	6	23	6
Participants with ≥ 2-fold rise[4], 95% CI	77.8% (40.0%-97.2%)	77.8% (40.0%-97.2%)	87.5% (47.3%-99.7%)	100.0% (54.1%-100.0%)	73.9% (51.6%-89.8%)	83.3% (35.9%-99.6%)
Participants with ≥4-fold rise[4], 95% CI	55.6% (21.2%-86.3%)	77.8% (40.0%-97.2%)	62.5% (24.5%-91.5%)	66.7% (22.3%-95.7%)	60.9% (38.5%-80.3%)	83.3% (35.9%-99.6%)
Geometric Mean Fold Rise[4], 95% CI	6.61 (2.29-19.04)	9.17 (2.83-29.73)	11.91 (2.91-48.80)	10.63 (2.31-48.80)	9.07 (4.30-19.14)	9.50 (1.90-47.58)
**Day 91 Visit (3 months post-boost)**						
N (non-missing)	10	9	7	6	20	5
Positive Response(%)[2]	9 (90.0%)	7 (77.8%)	7 (100.0%)	6 (100.0%)	19 (95.0%)	3 (60.0%)
Median (P_{25}, P_{75})	99.76 (60.41-1235.70)	27.58 (14.74-46.16)	424.71 (147.92-983.82)	1640.78 (38.49-2426.27)	663.18 (382.09-1974.66)	210.49 (5.00-281.11)
Minimum - Maximum	5.00-2956.65	5.00-206.28	31.05-3424.10	14.79-2908.78	5.00-34376.26	5.00-980.80
Geometric Mean (95% CI)	158.44 (35.65-704.10)	27.99 (10.35-75.73)	383.64 (93.72-1570.40)	456.03 (39.27-5295.93)	720.89 (311.88-1666.30)	67.97 (3.24-1426.52)
GM Fold Decrease Relative to D614G[3], 95% CI	3.83 (2.13-6.90)	5.09 (2.74-9.46)	3.97 (1.98-7.97)	4.03 (0.91-17.92)	3.10 (2.19-4.39)	9.42 (2.27-39.14)
N* (non-missing pre- and post-boost)	10	9	7	6	20	5

	Group 15E<br>Dosed Janssen<br>Boost Novavax<br>Age 18-55 yo<br>(N=11)	Group 15E<br>Dosed Janssen<br>Boost Novavax<br>Age ≥56 yo<br>(N=9)	Group 16E<br>Dosed Moderna<br>Boost Novavax<br>Age 18-55 yo<br>(N=9)	Group 16E<br>Dosed Moderna<br>Boost Novavax<br>Age ≥56 yo<br>(N=7)	Group 17E<br>Dosed Pfizer/BioNTech<br>Boost Novavax<br>Age 18-55 yo<br>(N=25)	Group 17E<br>Dosed Pfizer/BioNTech<br>Boost Novavax<br>Age ≥56 yo<br>(N=6)
Participants with ≥ 2-fold rise[4], 95% CI	70.0% (34.8%-93.3%)	77.8% (40.0%-97.2%)	71.4% (29.0%-96.3%)	100.0% (54.1%-100.0%)	55.0% (31.5%-76.9%)	40.0% (5.3%-85.3%)
Participants with ≥4-fold rise[4], 95% CI	50.0% (18.7%-81.3%)	66.7% (29.9%-92.5%)	57.1% (18.4%-90.1%)	66.7% (22.3%-95.7%)	50.0% (27.2%-72.8%)	40.0% (5.3%-85.3%)
Geometric Mean Fold Rise[4], 95% CI	8.33 (1.94-35.77)	4.26 (2.10-8.63)	10.86 (1.29-91.10)	15.34 (2.17-108.45)	5.92 (2.08-16.82)	2.67 (0.31-23.28)

[1] Values below the lower limit of detection (LLOD = 10) were assigned the value of LLOD/2.
[2] Positive response is defined as an ID titer above the LLOD.
[3] Relative to D614G titer at same visit, among participants with positive response to D614G.
[4] Relative to pre-vaccination (Day 1 Visit) levels, among participants with non-missing observations at both pre- and post-boost timepoints.

Supplementaryry Table 23: Neutralization Antibodies Titer (ID50) to Pseudovirus B.1.1.529 BA.2.75[1], by Group and Timepoint

	Group 15E Dosed Janssen Boost Novavax (N=20)	Group 16E Dosed Moderna Boost Novavax (N=16)	Group 17E Dosed Pfizer/BioNTech Boost Novavax (N=31)
**Day 1 Visit (Pre-boost)**			
N (non-missing)	20	16	31
Positive Response(%)[2]	11 (55.0%)	13 (81.3%)	25 (80.6%)
Median (P_{25}, P_{75})	12.06 (5.00-24.87)	41.52 (11.33-349.35)	26.61 (10.80-1244.99)
Minimum - Maximum	5.00-315.79	5.00-1339.50	5.00-21469.73
Geometric Mean (95% CI)	13.76 (7.63-24.81)	56.03 (20.18-155.58)	96.54 (36.78-253.40)
GM Fold Decrease Relative to D614G[3], 95% CI	5.89 (3.81-9.09)	6.20 (4.30-8.94)	3.84 (2.79-5.29)
**Day 29 Visit (28 days post-boost)**			
N (non-missing)	18	14	29
Positive Response(%)[2]	17 (94.4%)	14 (100.0%)	29 (100.0%)
Median (P_{25}, P_{75})	79.02 (34.67-198.42)	262.47 (113.57-1008.01)	727.28 (318.90-1811.54)
Minimum - Maximum	5.00-4298.39	46.19-3868.83	28.94-37187.07
Geometric Mean (95% CI)	80.14 (37.51-171.19)	351.27 (153.05-806.18)	766.27 (451.43-1300.67)
GM Fold Decrease Relative to D614G[3], 95% CI	6.23 (4.14-9.37)	5.63 (3.52-9.00)	3.50 (2.63-4.66)
N* (non-missing pre- and post-boost)	18	14	29
Participants with ≥ 2-fold rise[4], 95% CI	77.8% (52.4%-93.6%)	78.6% (49.2%-95.3%)	62.1% (42.3%-79.3%)
Participants with ≥ 4-fold rise[4], 95% CI	66.7% (41.0%-86.7%)	57.1% (28.9%-82.3%)	58.6% (38.9%-76.5%)
Geometric Mean Fold Rise[4], 95% CI	5.51 (3.18-9.52)	6.34 (2.89-13.93)	8.43 (4.14-17.14)
**Day 91 Visit (3 months post-boost)**			
N (non-missing)	19	13	25
Positive Response(%)[2]	17 (89.5%)	13 (100.0%)	25 (100.0%)
Median (P_{25}, P_{75})	41.70 (14.57-146.33)	955.52 (166.48-1698.96)	1076.04 (400.44-2323.48)
Minimum - Maximum	5.00-2978.48	24.15-3328.26	17.87-28805.48
Geometric Mean (95% CI)	64.73 (26.45-158.41)	522.70 (201.04-1359.06)	731.48 (370.39-1444.58)
GM Fold Decrease Relative to D614G[3], 95% CI	4.72 (3.09-7.22)	3.18 (1.81-5.58)	2.38 (1.76-3.22)
N* (non-missing pre- and post-boost)	19	13	25
Participants with ≥ 2-fold rise[4], 95% CI	78.9% (54.4%-93.9%)	76.9% (46.2%-95.0%)	60.0% (38.7%-78.9%)

	Group 15E Dosed Janssen Boost Novavax (N=20)	Group 16E Dosed Moderna Boost Novavax (N=16)	Group 17E Dosed Pfizer/BioNTech Boost Novavax (N=31)
Participants with ≥ 4-fold rise[4], 95% CI	42.1% (20.3%-66.5%)	69.2% (38.6%-90.9%)	56.0% (34.9%-75.6%)
Geometric Mean Fold Rise[4], 95% CI	4.70 (2.20-10.04)	9.17 (3.18-26.48)	6.71 (2.92-15.42)

[1] Values below the lower limit of detection (LLOD = 10) were assigned the value of LLOD/2.
[2] Positive response is defined as an ID titer above the LLOD.
[3] Relative to D614G titer at same visit, among participants with positive response to D614G.
[4] Relative to pre-vaccination (Day 1 Visit) levels, among participants with non-missing observations at both pre- and post-boost timepoints.

Supplementary Table 24: Neutralization Antibodies Titer (ID50) to Pseudovirus B.1.1.529 BA.2.75[1], by Group, Age Group and Timepoint

	Group 15E Dosed Janssen Boost Novavax Age 18-55 yo (N=11)	Group 15E Dosed Janssen Boost Novavax Age ≥56 yo (N=9)	Group 16E Dosed Moderna Boost Novavax Age 18-55 yo (N=9)	Group 16E Dosed Moderna Boost Novavax Age ≥56 yo (N=7)	Group 17E Dosed Pfizer/BioNTech Boost Novavax Age 18-55 yo (N=25)	Group 17E Dosed Pfizer/BioNTech Boost Novavax Age ≥56 yo (N=6)
**Day 1 Visit (Pre-boost)**						
N (non-missing)	11	9	9	7	25	6
Positive Response(%)[2]	9 (81.8%)	2 (22.2%)	9 (100.0%)	4 (57.1%)	22 (88.0%)	3 (50.0%)
Median (P_{25}, P_{75})	14.59 (12.53-55.89)	5.00 (5.00-5.00)	44.93 (26.27-740.77)	10.64 (5.00-258.28)	74.79 (16.79-1244.99)	7.90 (5.00-16.83)
Minimum - Maximum	5.00-315.79	5.00-32.27	12.02-1339.50	5.00-440.43	5.00-21469.73	5.00-2050.10
Geometric Mean (95% CI)	24.63 (9.87-61.43)	6.75 (4.10-11.12)	92.79 (23.63-364.36)	29.29 (4.51-190.09)	142.67 (49.09-414.65)	18.97 (1.61-223.21)
GM Fold Decrease Relative to D614G[3], 95% CI	5.70 (3.24-10.02)	6.13 (2.59-14.47)	4.53 (2.95-6.96)	9.27 (5.06-17.01)	3.61 (2.51-5.19)	5.27 (2.12-13.07)
**Day 29 Visit (28 days post-boost)**						
N (non-missing)	9	9	8	6	23	6
Positive Response(%)[2]	9 (100.0%)	8 (88.9%)	8 (100.0%)	6 (100.0%)	23 (100.0%)	6 (100.0%)
Median (P_{25}, P_{75})	98.70 (67.00-260.08)	37.46 (23.05-93.61)	633.95 (137.09-1378.72)	179.46 (50.93-335.67)	730.42 (318.90-2812.50)	537.00 (298.13-958.12)
Minimum - Maximum	11.65-4298.39	5.00-247.33	111.09-3132.99	46.19-3868.83	28.94-37187.07	112.85-1811.54
Geometric Mean (95% CI)	140.03 (38.91-503.98)	45.86 (18.04-116.61)	508.05 (175.56-1470.27)	214.75 (39.38-1171.13)	855.09 (450.86-1621.75)	503.26 (183.10-1383.21)
GM Fold Decrease Relative to D614G[3], 95% CI	5.07 (2.93-8.77)	7.65 (3.80-15.39)	4.04 (2.37-6.89)	8.77 (3.57-21.53)	3.46 (2.51-4.79)	3.64 (1.52-8.72)
N* (non-missing pre- and post-boost)	9	9	8	6	23	6
Participants with ≥ 2-fold rise[4], 95% CI	77.8% (40.0%-97.2%)	77.8% (40.0%-97.2%)	87.5% (47.3%-99.7%)	66.7% (22.3%-95.7%)	56.5% (34.5%-76.8%)	83.3% (35.9%-99.6%)
Participants with ≥4-fold rise[4], 95% CI	55.6% (21.2%-86.3%)	77.8% (40.0%-97.2%)	50.0% (15.7%-84.3%)	66.7% (22.3%-95.7%)	52.2% (30.6%-73.2%)	83.3% (35.9%-99.6%)
Geometric Mean Fold Rise[4], 95% CI	4.46 (2.00-9.97)	6.79 (2.75-16.78)	7.10 (2.49-20.25)	5.46 (1.01-29.42)	6.25 (2.85-13.69)	26.53 (4.24-165.93)
**Day 91 Visit (3 months post-boost)**						
N (non-missing)	10	9	7	6	20	5
Positive Response(%)[2]	9 (90.0%)	8 (88.9%)	7 (100.0%)	6 (100.0%)	20 (100.0%)	5 (100.0%)
Median (P_{25}, P_{75})	101.37 (41.70-880.66)	16.88 (13.65-38.54)	626.36 (149.74-1977.59)	1181.32 (166.48-1698.96)	1189.53 (427.26-2756.88)	490.54 (91.97-1168.75)
Minimum - Maximum	5.00-2978.48	5.00-146.33	37.72-2463.01	24.15-3328.26	17.87-28805.48	33.27-2323.48
Geometric Mean (95% CI)	155.90 (37.07-655.68)	24.37 (10.57-56.23)	490.75 (125.34-1921.41)	562.62 (81.83-3868.32)	890.72 (420.44-1887.02)	332.69 (37.17-2977.63)
GM Fold Decrease Relative to D614G[3], 95% CI	3.89 (2.25-6.73)	5.85 (2.72-12.56)	3.11 (1.90-5.07)	3.27 (0.82-13.05)	2.51 (1.78-3.55)	1.92 (0.77-4.79)
N* (non-missing pre- and post-boost)	10	9	7	6	20	5

	Group 15E Dosed Janssen Boost Novavax Age 18-55 yo (N=11)	Group 15E Dosed Janssen Boost Novavax Age ≥56 yo (N=9)	Group 16E Dosed Moderna Boost Novavax Age 18-55 yo (N=9)	Group 16E Dosed Moderna Boost Novavax Age ≥56 yo (N=7)	Group 17E Dosed Pfizer/BioNTech Boost Novavax Age 18-55 yo (N=25)	Group 17E Dosed Pfizer/BioNTech Boost Novavax Age ≥56 yo (N=6)
Participants with ≥ 2-fold rise[4], 95% CI	80.0% (44.4%-97.5%)	77.8% (40.0%-97.2%)	57.1% (18.4%-90.1%)	100.0% (54.1%-100.0%)	55.0% (31.5%-76.9%)	80.0% (28.4%-99.5%)
Participants with ≥4-fold rise[4], 95% CI	50.0% (18.7%-81.3%)	33.3% (7.5%-70.1%)	57.1% (18.4%-90.1%)	83.3% (35.9%-99.6%)	50.0% (27.2%-72.8%)	80.0% (28.4%-99.5%)
Geometric Mean Fold Rise[4], 95% CI	5.97 (1.45-24.58)	3.61 (1.66-7.85)	6.27 (1.13-34.80)	14.30 (2.45-83.50)	5.65 (2.22-14.35)	13.43 (0.91-199.28)

[1] Values below the lower limit of detection (LLOD = 10) were assigned the value of LLOD/2.
[2] Positive response is defined as an ID titer above the LLOD.
[3] Relative to D614G titer at same visit, among participants with positive response to D614G.
[4] Relative to pre-vaccination (Day 1 Visit) levels, among participants with non-missing observations at both pre- and post-boost timepoints.

Supplementary Table 25: Neutralization Antibodies Titer (ID50) to Pseudovirus Omicron BQ.1.1[1], by Group and Timepoint

	Group 15E Dosed Janssen Boost Novavax (N=20)	Group 16E Dosed Moderna Boost Novavax (N=16)	Group 17E Dosed Pfizer/BioNTech Boost Novavax (N=31)
**Day 1 Visit (Pre-boost)**			
N (non-missing)	20	16	31
Positive Response(%)[2]	5 (25.0%)	7 (43.8%)	17 (54.8%)
Median (P_{25}, P_{75})	5.00 (5.00-7.65)	5.00 (5.00-42.72)	21.70 (5.00-187.12)
Minimum - Maximum	5.00-29.68	5.00-146.84	5.00-80240.95
Geometric Mean (95% CI)	6.78 (5.15-8.92)	12.42 (6.53-23.63)	38.39 (15.77-93.45)
GM Fold Decrease Relative to D614G[3], 95% CI	12.21 (7.51-19.86)	27.96 (17.89-43.72)	9.97 (6.32-15.72)
**Day 29 Visit (28 days post-boost)**			
N (non-missing)	18	14	29
Positive Response(%)[2]	12 (66.7%)	13 (92.9%)	28 (96.6%)
Median (P_{25}, P_{75})	29.98 (5.00-48.23)	62.44 (17.39-176.30)	114.26 (35.43-256.75)
Minimum - Maximum	5.00-237.70	5.00-847.55	5.00-42994.42
Geometric Mean (95% CI)	21.69 (12.01-39.17)	56.47 (24.83-128.43)	126.19 (64.35-247.48)
GM Fold Decrease Relative to D614G[3], 95% CI	23.02 (15.43-34.33)	35.03 (18.04-68.03)	21.25 (14.32-31.54)
N* (non-missing pre- and post-boost)	18	14	29
Participants with ≥ 2-fold rise[4], 95% CI	61.1% (35.7%-82.7%)	78.6% (49.2%-95.3%)	58.6% (38.9%-76.5%)
Participants with ≥ 4-fold rise[4], 95% CI	44.4% (21.5%-69.2%)	50.0% (23.0%-77.0%)	44.8% (26.4%-64.3%)
Geometric Mean Fold Rise[4], 95% CI	3.09 (1.84-5.19)	5.08 (2.75-9.38)	3.40 (1.79-6.47)
**Day 91 Visit (3 months post-boost)**			
N (non-missing)	19	13	25
Positive Response(%)[2]	13 (68.4%)	11 (84.6%)	22 (88.0%)
Median (P_{25}, P_{75})	18.93 (5.00-48.94)	114.19 (19.05-260.49)	111.08 (39.70-385.63)
Minimum - Maximum	5.00-475.09	5.00-541.97	5.00-56288.02
Geometric Mean (95% CI)	22.66 (11.54-44.52)	76.16 (29.30-197.96)	128.25 (53.79-305.80)
GM Fold Decrease Relative to D614G[3], 95% CI	13.48 (8.62-21.09)	21.81 (11.05-43.05)	13.58 (8.79-20.97)
N* (non-missing pre- and post-boost)	19	13	25
Participants with ≥ 2-fold rise[4], 95% CI	57.9% (33.5%-79.7%)	76.9% (46.2%-95.0%)	48.0% (27.8%-68.7%)

	Group 15E<br>Dosed Janssen<br>Boost Novavax<br>(N=20)	Group 16E<br>Dosed Moderna<br>Boost Novavax<br>(N=16)	Group 17E<br>Dosed Pfizer/BioNTech<br>Boost Novavax<br>(N=31)
Participants with ≥ 4-fold rise[4], 95% CI	26.3% (9.1%-51.2%)	53.8% (25.1%-80.8%)	44.0% (24.4%-65.1%)
Geometric Mean Fold Rise[4], 95% CI	3.29 (1.67-6.47)	6.45 (2.45-16.97)	2.77 (1.18-6.49)

[1] Values below the lower limit of detection (LLOD = 10) were assigned the value of LLOD/2.
[2] Positive response is defined as an ID titer above the LLOD.
[3] Relative to D614G titer at same visit, among participants with positive response to D614G.
[4] Relative to pre-vaccination (Day 1 Visit) levels, among participants with non-missing observations at both pre- and post-boost timepoints.

Supplementary Table 26: Neutralization Antibodies Titer (ID50) to Pseudovirus Omicron BQ.1.1[1], by Group and Timepoint

	Group 15E Dosed Janssen Boost Novavax Age 18-55 yo (N=11)	Group 15E Dosed Janssen Boost Novavax Age ≥56 yo (N=9)	Group 16E Dosed Moderna Boost Novavax Age 18-55 yo (N=9)	Group 16E Dosed Moderna Boost Novavax Age ≥56 yo (N=7)	Group 17E Dosed Pfizer/BioNTech Boost Novavax Age 18-55 yo (N=25)	Group 17E Dosed Pfizer/BioNTech Boost Novavax Age ≥56 yo (N=6)
**Day 1 Visit (Pre-boost)**						
N (non-missing)	11	9	9	7	25	6
Positive Response(%)[2]	4 (36.4%)	1 (11.1%)	4 (44.4%)	3 (42.9%)	15 (60.0%)	2 (33.3%)
Median (P_{25}, P_{75})	5.00 (5.00-15.92)	5.00 (5.00-5.00)	5.00 (5.00-54.31)	5.00 (5.00-38.13)	22.15 (5.00-187.12)	5.00 (5.00-159.82)
Minimum - Maximum	5.00-29.68	5.00-10.31	5.00-146.84	5.00-47.30	5.00-80240.95	5.00-219.90
Geometric Mean (95% CI)	8.15 (5.01-13.24)	5.42 (4.50-6.52)	14.29 (4.94-41.36)	10.37 (4.07-26.43)	46.85 (16.48-133.22)	16.73 (2.34-119.63)
GM Fold Decrease Relative to D614G[3], 95% CI	17.40 (7.71-39.26)	7.85 (5.15-11.96)	29.43 (14.73-58.77)	26.19 (12.29-55.81)	10.99 (6.89-17.52)	6.12 (0.78-48.23)
**Day 29 Visit (28 days post-boost)**						
N (non-missing)	9	9	8	6	23	6
Positive Response(%)[2]	8 (88.9%)	4 (44.4%)	7 (87.5%)	6 (100.0%)	23 (100.0%)	5 (83.3%)
Median (P_{25}, P_{75})	35.94 (25.07-38.12)	5.00 (5.00-48.23)	67.85 (30.98-191.59)	47.57 (17.00-88.46)	147.20 (56.43-422.41)	28.94 (16.14-71.76)
Minimum - Maximum	5.00-86.85	5.00-237.70	5.00-294.06	10.23-847.55	15.47-42994.42	5.00-163.51
Geometric Mean (95% CI)	30.07 (16.48-54.84)	15.65 (5.07-48.30)	60.93 (19.59-189.53)	51.02 (9.20-282.98)	183.31 (87.20-385.36)	30.16 (8.46-107.59)
GM Fold Decrease Relative to D614G[3], 95% CI	23.62 (11.64-47.94)	22.43 (12.85-39.14)	33.68 (17.76-63.87)	36.92 (6.78-201.14)	16.16 (11.11-23.50)	60.72 (20.52-179.62)
N* (non-missing pre- and post-boost)	9	9	8	6	23	6
Participants with ≥ 2-fold rise[4], 95% CI	77.8% (40.0%-97.2%)	44.4% (13.7%-78.8%)	75.0% (34.9%-96.8%)	83.3% (35.9%-99.6%)	60.9% (38.5%-80.3%)	50.0% (11.8%-88.2%)
Participants with ≥4-fold rise[4], 95% CI	44.4% (13.7%-78.8%)	44.4% (13.7%-78.8%)	62.5% (24.5%-91.5%)	33.3% (4.3%-77.7%)	47.8% (26.8%-69.4%)	33.3% (4.3%-77.7%)
Geometric Mean Fold Rise[4], 95% CI	3.31 (1.85-5.93)	2.89 (1.06-7.91)	5.70 (2.08-15.62)	4.36 (1.66-11.46)	4.02 (1.85-8.72)	1.80 (0.57-5.75)
**Day 91 Visit (3 months post-boost)**						
N (non-missing)	10	9	7	6	20	5
Positive Response(%)[2]	9 (90.0%)	4 (44.4%)	6 (85.7%)	5 (83.3%)	19 (95.0%)	3 (60.0%)
Median (P_{25}, P_{75})	38.19 (18.93-52.38)	5.00 (5.00-18.84)	114.19 (19.05-272.82)	152.24 (16.97-260.49)	123.69 (70.14-543.42)	26.14 (5.00-32.51)
Minimum - Maximum	5.00-475.09	5.00-68.94	5.00-541.97	5.00-348.05	5.00-56288.02	5.00-115.27
Geometric Mean (95% CI)	43.87 (16.03-120.10)	10.88 (4.98-23.77)	78.10 (17.69-344.81)	73.96 (12.38-441.84)	206.84 (80.97-528.40)	18.96 (3.58-100.35)
GM Fold Decrease Relative to D614G[3], 95% CI	13.84 (6.31-30.35)	13.10 (7.30-23.52)	19.51 (8.84-43.06)	24.85 (5.39-114.55)	10.81 (6.64-17.61)	33.77 (19.53-58.40)
N* (non-missing pre- and post-boost)	10	9	7	6	20	5

	Group 15E Dosed Janssen Boost Novavax Age 18-55 yo (N=11)	Group 15E Dosed Janssen Boost Novavax Age ≥56 yo (N=9)	Group 16E Dosed Moderna Boost Novavax Age 18-55 yo (N=9)	Group 16E Dosed Moderna Boost Novavax Age ≥56 yo (N=7)	Group 17E Dosed Pfizer/BioNTech Boost Novavax Age 18-55 yo (N=25)	Group 17E Dosed Pfizer/BioNTech Boost Novavax Age ≥56 yo (N=6)
Participants with ≥ 2-fold rise[4], 95% CI	70.0% (34.8%-93.3%)	44.4% (13.7%-78.8%)	71.4% (29.0%-96.3%)	83.3% (35.9%-99.6%)	55.0% (31.5%-76.9%)	20.0% (0.5%-71.6%)
Participants with ≥4-fold rise[4], 95% CI	30.0% (6.7%-65.2%)	22.2% (2.8%-60.0%)	42.9% (9.9%-81.6%)	66.7% (22.3%-95.7%)	50.0% (27.2%-72.8%)	20.0% (0.5%-71.6%)
Geometric Mean Fold Rise[4], 95% CI	5.13 (1.55-16.96)	2.01 (1.03-3.92)	6.56 (1.01-42.44)	6.31 (1.94-20.56)	3.68 (1.34-10.09)	0.89 (0.20-3.88)

[1] Values below the lower limit of detection (LLOD = 10) were assigned the value of LLOD/2.
[2] Positive response is defined as an ID titer above the LLOD.
[3] Relative to D614G titer at same visit, among participants with positive response to D614G.
[4] Relative to pre-vaccination (Day 1 Visit) levels, among participants with non-missing observations at both pre- and post-boost timepoints.

Supplementary Table 27: Neutralization Antibodies Titer (ID50) to Pseudovirus Omicron XBB.1[1], by Group and Timepoint

	Group 15E Dosed Janssen Boost Novavax (N=20)	Group 16E Dosed Moderna Boost Novavax (N=16)	Group 17E Dosed Pfizer/BioNTech Boost Novavax (N=31)
**Day 1 Visit (Pre-boost)**			
N (non-missing)	20	16	31
Positive Response(%)[2]	3 (15.0%)	5 (31.3%)	16 (51.6%)
Median (P_{25}, P_{75})	5.00 (5.00-5.00)	5.00 (5.00-30.10)	20.04 (5.00-64.37)
Minimum - Maximum	5.00-42.53	5.00-495.68	5.00-85936.77
Geometric Mean (95% CI)	6.33 (4.77-8.40)	10.98 (5.22-23.08)	23.99 (11.02-52.22)
GM Fold Decrease Relative to D614G[3], 95% CI	13.18 (7.90-21.97)	31.65 (19.26-52.00)	16.20 (8.89-29.52)
**Day 29 Visit (28 days post-boost)**			
N (non-missing)	18	14	29
Positive Response(%)[2]	5 (27.8%)	10 (71.4%)	24 (82.8%)
Median (P_{25}, P_{75})	5.00 (5.00-12.78)	24.37 (5.00-98.59)	32.35 (20.61-104.28)
Minimum - Maximum	5.00-184.26	5.00-395.86	5.00-25262.69
Geometric Mean (95% CI)	8.09 (5.03-12.99)	27.29 (11.54-64.54)	40.69 (21.34-77.60)
GM Fold Decrease Relative to D614G[3], 95% CI	61.73 (37.39-101.93)	72.49 (38.53-136.38)	65.90 (44.65-97.27)
N* (non-missing pre- and post-boost)	18	14	29
Participants with ≥ 2-fold rise[4], 95% CI	16.7% (3.6%-41.4%)	57.1% (28.9%-82.3%)	41.4% (23.5%-61.1%)
Participants with ≥ 4-fold rise[4], 95% CI	11.1% (1.4%-34.7%)	42.9% (17.7%-71.1%)	31.0% (15.3%-50.8%)
Geometric Mean Fold Rise[4], 95% CI	1.24 (0.91-1.70)	3.09 (1.65-5.78)	1.75 (1.00-3.07)
**Day 91 Visit (3 months post-boost)**			
N (non-missing)	19	13	25
Positive Response(%)[2]	6 (31.6%)	9 (69.2%)	20 (80.0%)
Median (P_{25}, P_{75})	5.00 (5.00-19.03)	59.71 (5.00-125.64)	34.11 (17.16-71.82)
Minimum - Maximum	5.00-136.71	5.00-268.24	5.00-32252.62
Geometric Mean (95% CI)	9.57 (5.62-16.29)	37.00 (14.97-91.45)	40.26 (18.82-86.16)
GM Fold Decrease Relative to D614G[3], 95% CI	31.93 (18.42-55.36)	44.90 (23.93-84.24)	43.26 (29.00-64.53)
N* (non-missing pre- and post-boost)	19	13	25
Participants with ≥ 2-fold rise[4], 95% CI	26.3% (9.1%-51.2%)	53.8% (25.1%-80.8%)	36.0% (18.0%-57.5%)

	Group 15E<br>Dosed Janssen<br>Boost Novavax<br>(N=20)	Group 16E<br>Dosed Moderna<br>Boost Novavax<br>(N=16)	Group 17E<br>Dosed Pfizer/BioNTech<br>Boost Novavax<br>(N=31)
Participants with ≥ 4-fold rise[4], 95% CI	15.8% (3.4%-39.6%)	46.2% (19.2%-74.9%)	32.0% (14.9%-53.5%)
Geometric Mean Fold Rise[4], 95% CI	1.49 (0.91-2.45)	4.00 (1.69-9.49)	1.44 (0.72-2.87)

[1] Values below the lower limit of detection (LLOD = 10) were assigned the value of LLOD/2.
[2] Positive response is defined as an ID titer above the LLOD.
[3] Relative to D614G titer at same visit, among participants with positive response to D614G.
[4] Relative to pre-vaccination (Day 1 Visit) levels, among participants with non-missing observations at both pre- and post-boost timepoints.

Supplementary Table 28: Neutralization Antibodies Titer (ID50) to Pseudovirus Omicron XBB.1[1], by Group, Age Group and Timepoint

	Group 15E Dosed Janssen Boost Novavax Age 18-55 yo (N=11)	Group 15E Dosed Janssen Boost Novavax Age ≥56 yo (N=9)	Group 16E Dosed Moderna Boost Novavax Age 18-55 yo (N=9)	Group 16E Dosed Moderna Boost Novavax Age ≥56 yo (N=7)	Group 17E Dosed Pfizer/BioNTech Boost Novavax Age 18-55 yo (N=25)	Group 17E Dosed Pfizer/BioNTech Boost Novavax Age ≥56 yo (N=6)
**Day 1 Visit (Pre-boost)**						
N (non-missing)	11	9	9	7	25	6
Positive Response(%)[2]	3 (27.3%)	0 (0.0%)	3 (33.3%)	2 (28.6%)	14 (56.0%)	2 (33.3%)
Median (P_{25}, P_{75})	5.00 (5.00-14.44)	5.00 (5.00-5.00)	5.00 (5.00-49.35)	5.00 (5.00-10.85)	20.89 (5.00-59.85)	5.00 (5.00-64.37)
Minimum - Maximum	5.00-42.53	5.00-5.00	5.00-495.68	5.00-63.56	5.00-85936.77	5.00-146.17
Geometric Mean (95% CI)	7.68 (4.57-12.93)	5.00 (5.00-5.00)	13.99 (3.86-50.78)	8.03 (3.32-19.46)	27.58 (10.94-69.49)	13.44 (2.63-68.56)
GM Fold Decrease Relative to D614G[3], 95% CI	18.56 (7.89-43.65)	8.59 (5.23-14.11)	30.05 (14.33-63.00)	33.82 (13.89-82.35)	18.67 (9.87-35.32)	7.97 (0.74-85.39)
**Day 29 Visit (28 days post-boost)**						
N (non-missing)	9	9	8	6	23	6
Positive Response(%)[2]	4 (44.4%)	1 (11.1%)	6 (75.0%)	4 (66.7%)	21 (91.3%)	3 (50.0%)
Median (P_{25}, P_{75})	5.00 (5.00-20.44)	5.00 (5.00-5.00)	33.36 (13.28-129.69)	13.99 (5.00-33.45)	44.65 (22.18-130.46)	13.39 (5.00-32.35)
Minimum - Maximum	5.00-184.26	5.00-12.78	5.00-215.20	5.00-395.86	5.00-25262.69	5.00-42.58
Geometric Mean (95% CI)	11.79 (4.57-30.36)	5.55 (4.36-7.06)	34.44 (10.30-115.08)	20.02 (3.62-110.82)	55.41 (26.38-116.38)	12.46 (4.26-36.47)
GM Fold Decrease Relative to D614G[3], 95% CI	60.26 (23.37-155.39)	63.24 (34.17-117.04)	59.60 (26.31-135.01)	94.11 (24.62-359.73)	53.46 (35.05-81.56)	146.93 (61.20-352.72)
N* (non-missing pre- and post-boost)	9	9	8	6	23	6
Participants with ≥ 2-fold rise[4], 95% CI	22.2% (2.8%-60.0%)	11.1% (0.3%-48.2%)	62.5% (24.5%-91.5%)	50.0% (11.8%-88.2%)	47.8% (26.8%-69.4%)	16.7% (0.4%-64.1%)
Participants with ≥4-fold rise[4], 95% CI	22.2% (2.8%-60.0%)	0.0% (0.0%-33.6%)	50.0% (15.7%-84.3%)	33.3% (4.3%-77.7%)	34.8% (16.4%-57.3%)	16.7% (0.4%-64.1%)
Geometric Mean Fold Rise[4], 95% CI	1.39 (0.73-2.66)	1.11 (0.87-1.41)	3.84 (1.37-10.80)	2.30 (0.92-5.73)	2.07 (1.07-3.98)	0.93 (0.26-3.28)
**Day 91 Visit (3 months post-boost)**						
N (non-missing)	10	9	7	6	20	5
Positive Response(%)[2]	5 (50.0%)	1 (11.1%)	5 (71.4%)	4 (66.7%)	17 (85.0%)	3 (60.0%)
Median (P_{25}, P_{75})	8.74 (5.00-44.90)	5.00 (5.00-5.00)	68.75 (5.00-125.64)	54.19 (5.00-125.69)	46.51 (22.50-95.54)	17.16 (5.00-34.11)
Minimum - Maximum	5.00-136.71	5.00-19.03	5.00-268.24	5.00-167.21	5.00-32252.62	5.00-35.94
Geometric Mean (95% CI)	15.01 (5.77-39.06)	5.80 (4.12-8.17)	39.84 (9.33-170.17)	33.93 (6.66-172.95)	52.49 (21.29-129.42)	13.94 (4.13-47.07)
GM Fold Decrease Relative to D614G[3], 95% CI	40.44 (16.15-101.26)	24.56 (11.69-51.61)	38.25 (15.22-96.12)	54.15 (16.13-181.83)	42.61 (25.83-70.31)	45.94 (26.84-78.64)
N* (non-missing pre- and post-boost)	10	9	7	6	20	5

	Group 15E Dosed Janssen Boost Novavax Age 18-55 yo (N=11)	Group 15E Dosed Janssen Boost Novavax Age ≥56 yo (N=9)	Group 16E Dosed Moderna Boost Novavax Age 18-55 yo (N=9)	Group 16E Dosed Moderna Boost Novavax Age ≥56 yo (N=7)	Group 17E Dosed Pfizer/BioNTech Boost Novavax Age 18-55 yo (N=25)	Group 17E Dosed Pfizer/BioNTech Boost Novavax Age ≥56 yo (N=6)
Participants with ≥ 2-fold rise[4], 95% CI	40.0% (12.2%-73.8%)	11.1% (0.3%-48.2%)	42.9% (9.9%-81.6%)	66.7% (22.3%-95.7%)	40.0% (19.1%-63.9%)	20.0% (0.5%-71.6%)
Participants with ≥4-fold rise[4], 95% CI	30.0% (6.7%-65.2%)	0.0% (0.0%-33.6%)	42.9% (9.9%-81.6%)	50.0% (11.8%-88.2%)	35.0% (15.4%-59.2%)	20.0% (0.5%-71.6%)
Geometric Mean Fold Rise[4], 95% CI	1.87 (0.71-4.92)	1.16 (0.82-1.63)	4.09 (0.91-18.42)	3.90 (0.99-15.47)	1.64 (0.73-3.69)	0.85 (0.14-5.19)

[1] Values below the lower limit of detection (LLOD = 10) were assigned the value of LLOD/2.
[2] Positive response is defined as an ID titer above the LLOD.
[3] Relative to D614G titer at same visit, among participants with positive response to D614G.
[4] Relative to pre-vaccination (Day 1 Visit) levels, among participants with non-missing observations at both pre- and post-boost timepoints.

Supplementary Table 29: Percent of CD4+ Cells Expressing IL2 and/or IFNγ in Response to Spike peptide pool (S1 + S2) by ICS, by Group and Timepoint

	Group 15E [Dosed Janssen, Boost Novavax] (N=20)	Group 16E [Dosed Moderna, Boost Novavax] (N=16)	Group 17E [Dosed Pfizer, Boost Novavax] (N=31)
**Day 1 Visit (Pre-boost)**			
**Participants with available sample[1]**			
N	20	16	29
Median (P_{25}, P_{75})	0.06 (0.04-0.08)	0.24 (0.14-0.46)	0.13 (0.07-0.22)
Minimum - Maximum	0.01-0.33	0.04-0.98	-0.06-0.65
**Participants with positive response[2]**			
N (%)	10 (50%)	14 (88%)	24 (83%)
Median (P_{25}, P_{75})	0.07 (0.06-0.15)	0.28 (0.19-0.52)	0.15 (0.11-0.22)
Minimum - Maximum	0.05-0.33	0.10-0.98	0.07-0.65
**Day 15 Visit (14 days post-boost)**			
**Participants with available sample[1]**			
N	19	15	29
Median (P_{25}, P_{75})	0.36 (0.16-0.46)	0.47 (0.24-0.68)	0.34 (0.21-0.46)
Minimum - Maximum	0.10-0.55	0.11-1.08	-0.13-1.43
**Participants with positive response[2]**			
N (%)	19 (100%)	15 (100%)	27 (93%)
Median (P_{25}, P_{75})	0.36 (0.16-0.46)	0.47 (0.24-0.68)	0.36 (0.23-0.60)
Minimum - Maximum	0.10-0.55	0.11-1.08	0.11-1.43

[1] Includes participants with assayed samples, excluding those for which the assay result was deemed unreliable.

[2] Defined as percent of cells expressing cytokine in response to the peptide pool being statistically significantly higher than in response to the negative control, by Fisher's exact test, while adjusting p-values for multiple comparisons.

Supplementary Table 30: Percent of CD4+ Cells Expressing IL2 and/or IFNγ in Response to Spike peptide pool (S1 + S2) by ICS, by Group, Age Group and Timepoint

	Group 15E [Dosed Janssen, Boost Novavax] Age 18-55 yo (N=11)	Group 15E [Dosed Janssen, Boost Novavax] Age ≥56 yo (N=9)	Group 16E [Dosed Moderna, Boost Novavax] Age 18-55 yo (N=9)	Group 16E [Dosed Moderna, Boost Novavax] Age ≥56 yo (N=7)	Group 17E [Dosed Pfizer, Boost Novavax] Age 18-55 yo (N=25)	Group 17E [Dosed Pfizer, Boost Novavax] Age ≥56 yo (N=6)
**Day 1 Visit (Pre-boost)**						
**Participants with available sample[1]**						
N	11	9	9	7	23	6
Median (P_{25}, P_{75})	0.06 (0.03-0.15)	0.06 (0.05-0.07)	0.30 (0.19-0.52)	0.23 (0.10-0.38)	0.13 (0.09-0.22)	0.09 (0.04-0.13)
Minimum - Maximum	0.01-0.33	0.04-0.08	0.04-0.98	0.05-0.84	-0.06-0.65	0.04-0.27
**Participants with positive response[2]**						
N (%)	5 (45%)	5 (56%)	8 (89%)	6 (86%)	20 (87%)	4 (67%)
Median (P_{25}, P_{75})	0.15 (0.15-0.16)	0.07 (0.06-0.07)	0.35 (0.21-0.67)	0.24 (0.16-0.38)	0.18 (0.11-0.22)	0.12 (0.09-0.20)
Minimum - Maximum	0.06-0.33	0.05-0.08	0.11-0.98	0.10-0.84	0.07-0.65	0.07-0.27
**Day 15 Visit (14 days post-boost)**						
**Participants with available sample[1]**						
N	10	9	9	6	23	6
Median (P_{25}, P_{75})	0.38 (0.27-0.42)	0.32 (0.13-0.46)	0.54 (0.35-0.74)	0.33 (0.21-0.59)	0.39 (0.21-0.64)	0.28 (0.18-0.31)
Minimum - Maximum	0.16-0.53	0.10-0.55	0.11-1.08	0.14-0.63	-0.13-1.43	0.15-0.34
**Participants with positive response[2]**						
N (%)	10 (100%)	9 (100%)	9 (100%)	6 (100%)	21 (91%)	6 (100%)
Median (P_{25}, P_{75})	0.38 (0.27-0.42)	0.32 (0.13-0.46)	0.54 (0.35-0.74)	0.33 (0.21-0.59)	0.41 (0.24-0.64)	0.28 (0.18-0.31)
Minimum - Maximum	0.16-0.53	0.10-0.55	0.11-1.08	0.14-0.63	0.11-1.43	0.15-0.34

[1] Includes participants with assayed samples, excluding those for which the assay result was deemed unreliable.
[2] Defined as percent of cells expressing cytokine in response to the peptide pool being statistically significantly higher than in response to the negative control, by Fisher's exact test, while adjusting p-values for multiple comparisons.

Supplementary Table 31: Percent of CD4+ Cells Expressing IL-4 and/or IL-5/IL-13 and CD154 in Response to Spike peptide pool (S1 + S2) by ICS, by Group and Timepoint

	Group 15E [Dosed Janssen, Boost Novavax] (N=20)	Group 16E [Dosed Moderna, Boost Novavax] (N=16)	Group 17E [Dosed Pfizer, Boost Novavax] (N=31)
**Day 1 Visit (Pre-boost)**			
**Participants with available sample[1]**			
N	20	16	29
Median (P_{25}, P_{75})	-0.00 (-0.01-0.01)	0.01 (0.00-0.02)	0.00 (-0.00-0.01)
Minimum - Maximum	-0.10-0.08	-0.02-0.08	-0.02-0.05
**Participants with positive response[2]**			
N (%)	1 (5%)	1 (6%)	0 (0%)
Median (P_{25}, P_{75})	0.08 (0.08-0.08)	0.08 (0.08-0.08)	
Minimum - Maximum	0.08-0.08	0.08-0.08	
**Day 15 Visit (14 days post-boost)**			
**Participants with available sample[1]**			
N	19	15	29
Median (P_{25}, P_{75})	0.01 (-0.00-0.02)	0.01 (-0.00-0.03)	0.01 (0.00-0.03)
Minimum - Maximum	-0.03-0.04	-0.01-0.11	-0.02-0.17
**Participants with positive response[2]**			
N (%)	1 (5%)	3 (20%)	5 (17%)
Median (P_{25}, P_{75})	0.04 (0.04-0.04)	0.07 (0.07-0.11)	0.09 (0.07-0.10)
Minimum - Maximum	0.04-0.04	0.07-0.11	0.05-0.17

[1] Includes participants with assayed samples, excluding those for which the assay result was deemed unreliable.

[2] Defined as percent of cells expressing cytokine in response to the peptide pool being statistically significantly higher than in response to the negative control, by Fisher's exact test, while adjusting p-values for multiple comparisons.

Supplementary Table 32: Percent of CD4+ Cells Expressing IL2 and/or IFNγ in Response to Spike peptide pool (S1 + S2) by ICS, by Group and Timepoint

	Group 15E [Dosed Janssen, Boost Novavax] Age 18-55 yo (N=11)	Group 15E [Dosed Janssen, Boost Novavax] Age ≥56 yo (N=9)	Group 16E [Dosed Moderna, Boost Novavax] Age 18-55 yo (N=9)	Group 16E [Dosed Moderna, Boost Novavax] Age ≥56 yo (N=7)	Group 17E [Dosed Pfizer, Boost Novavax] Age 18-55 yo (N=25)	Group 17E [Dosed Pfizer, Boost Novavax] Age ≥56 yo (N=6)
**Day 1 Visit (Pre-boost)**						
**Participants with available sample[1]**						
N	11	9	9	7	23	6
Median (P_{25}, P_{75})	-0.00 (-0.01-0.01)	-0.00 (-0.01-0.01)	0.01 (0.01-0.02)	0.00 (0.00-0.03)	0.00 (-0.00-0.01)	-0.00 (-0.01-0.00)
Minimum - Maximum	-0.04-0.02	-0.10-0.08	-0.02-0.08	-0.02-0.03	-0.02-0.05	-0.01-0.01
**Participants with positive response[2]**						
N (%)	0 (0%)	1 (11%)	1 (11%)	0 (0%)	0 (0%)	0 (0%)
Median (P_{25}, P_{75})		0.08 (0.08-0.08)	0.08 (0.08-0.08)			
Minimum - Maximum		0.08-0.08	0.08-0.08			
**Day 15 Visit (14 days post-boost)**						
**Participants with available sample[1]**						
N	10	9	9	6	23	6
Median (P_{25}, P_{75})	0.01 (-0.00-0.02)	0.01 (-0.00-0.01)	0.02 (0.00-0.07)	0.00 (-0.01-0.01)	0.01 (0.00-0.04)	0.01 (0.01-0.03)
Minimum - Maximum	-0.02-0.04	-0.03-0.04	-0.00-0.11	-0.01-0.03	-0.02-0.17	-0.01-0.05
**Participants with positive response[2]**						
N (%)	0 (0%)	1 (11%)	3 (33%)	0 (0%)	4 (17%)	1 (17%)
Median (P_{25}, P_{75})		0.04 (0.04-0.04)	0.07 (0.07-0.11)		0.10 (0.08-0.14)	0.05 (0.05-0.05)
Minimum - Maximum		0.04-0.04	0.07-0.11		0.07-0.17	0.05-0.05

[1] Includes participants with assayed samples, excluding those for which the assay result was deemed unreliable.
[2] Defined as percent of cells expressing cytokine in response to the peptide pool being statistically significantly higher than in response to the negative control, by Fisher's exact test, while adjusting p-values for multiple comparisons.

Supplementary Table 33: Percent of CD8+ Cells Expressing IL2 and/or IFNγ in Response to Spike peptide pool (S1 + S2) by ICS, by Group and Timepoint

	Group 15E [Dosed Janssen, Boost Novavax] (N=20)	Group 16E [Dosed Moderna, Boost Novavax] (N=16)	Group 17E [Dosed Pfizer, Boost Novavax] (N=31)
**Day 1 Visit (Pre-boost)**			
**Participants with available sample[1]**			
N	20	16	29
Median (P_{25}, P_{75})	0.09 (0.01-0.37)	0.04 (0.02-0.07)	0.03 (0.02-0.08)
Minimum - Maximum	0.00-1.20	-0.01-0.37	-0.01-0.51
**Participants with positive response[2]**			
N (%)	11 (55%)	2 (13%)	8 (28%)
Median (P_{25}, P_{75})	0.34 (0.08-0.79)	0.22 (0.08-0.37)	0.23 (0.10-0.35)
Minimum - Maximum	0.07-1.20	0.08-0.37	0.06-0.51
**Day 15 Visit (14 days post-boost)**			
**Participants with available sample[1]**			
N	19	15	29
Median (P_{25}, P_{75})	0.13 (0.02-0.36)	0.06 (0.02-0.08)	0.02 (0.00-0.08)
Minimum - Maximum	0.01-1.25	-0.01-0.45	-0.02-0.46
**Participants with positive response[2]**			
N (%)	11 (58%)	5 (33%)	8 (28%)
Median (P_{25}, P_{75})	0.29 (0.14-0.99)	0.08 (0.07-0.15)	0.26 (0.17-0.39)
Minimum - Maximum	0.07-1.25	0.07-0.45	0.08-0.46

[1] Includes participants with assayed samples, excluding those for which the assay result was deemed unreliable.
[2] Defined as percent of cells expressing cytokine in response to the peptide pool being statistically significantly higher than in response to the negative control, by Fisher's exact test, while adjusting p-values for multiple comparisons.

Supplementary Table 34: Percent of CD8+ Cells Expressing IL2 and/or IFNγ in Response to Spike peptide pool (S1 + S2) by ICS, by Group, Age Group and Timepoint

	Group 15E [Dosed Janssen, Boost Novavax] Age 18-55 yo (N=11)	Group 15E [Dosed Janssen, Boost Novavax] Age ≥56 yo (N=9)	Group 16E [Dosed Moderna, Boost Novavax] Age 18-55 yo (N=9)	Group 16E [Dosed Moderna, Boost Novavax] Age ≥56 yo (N=7)	Group 17E [Dosed Pfizer, Boost Novavax] Age 18-55 yo (N=25)	Group 17E [Dosed Pfizer, Boost Novavax] Age ≥56 yo (N=6)
**Day 1 Visit (Pre-boost)**						
**Participants with available sample[1]**						
N	11	9	9	7	23	6
Median (P_{25}, P_{75})	0.02 (0.00-0.34)	0.16 (0.08-0.47)	0.04 (0.01-0.06)	0.05 (0.02-0.08)	0.03 (0.02-0.08)	0.05 (-0.00-0.10)
Minimum - Maximum	0.00-1.04	0.05-1.20	-0.01-0.37	-0.01-0.08	-0.01-0.36	-0.00-0.51
**Participants with positive response[2]**						
N (%)	5 (45%)	6 (67%)	1 (11%)	1 (14%)	6 (26%)	2 (33%)
Median (P_{25}, P_{75})	0.34 (0.26-0.40)	0.32 (0.08-0.79)	0.37 (0.37-0.37)	0.08 (0.08-0.08)	0.23 (0.12-0.34)	0.29 (0.08-0.51)
Minimum - Maximum	0.08-1.04	0.07-1.20	0.37-0.37	0.08-0.08	0.06-0.36	0.08-0.51
**Day 15 Visit (14 days post-boost)**						
**Participants with available sample[1]**						
N	10	9	9	6	23	6
Median (P_{25}, P_{75})	0.07 (0.02-0.36)	0.14 (0.10-0.28)	0.04 (0.02-0.07)	0.07 (0.06-0.08)	0.02 (0.00-0.16)	0.05 (0.00-0.08)
Minimum - Maximum	0.01-0.99	0.02-1.25	0.01-0.45	-0.01-0.15	-0.02-0.46	-0.01-0.34
**Participants with positive response[2]**						
N (%)	5 (50%)	6 (67%)	2 (22%)	3 (50%)	6 (26%)	2 (33%)
Median (P_{25}, P_{75})	0.36 (0.29-0.38)	0.21 (0.14-1.04)	0.26 (0.07-0.45)	0.08 (0.07-0.15)	0.26 (0.19-0.43)	0.21 (0.08-0.34)
Minimum - Maximum	0.07-0.99	0.13-1.25	0.07-0.45	0.07-0.15	0.16-0.46	0.08-0.34

[1] Includes participants with assayed samples, excluding those for which the assay result was deemed unreliable.
[2] Defined as percent of cells expressing cytokine in response to the peptide pool being statistically significantly higher than in response to the negative control, by Fisher's exact test, while adjusting p-values for multiple comparisons.
