# Supplementary material for: Immunogenicity of NVX-CoV2373 heterologous boost against SARS-CoV-2 variants
Source: NPJ Vaccines. 2023 Jul 11;8:98. doi: 10.1038/s41541-023-00693-z (PMC10336079; doi:10.1038/s41541-023-00693-z)
Supplement: Supplementary file 2 — REPORTING SUMMARY [file 41541_2023_693_MOESM2_ESM.pdf]

nature portfolio

Corresponding author(s): Lyke, KE
Atmar, RL

Last updated by author(s): 26 May 2023

Reporting Summary

Nature Portfolio wishes to improve the reproducibility of the work that we publish. This form provides structure for consistency and transparency in reporting. For further information on Nature Portfolio policies, see our Editorial Policies and the Editorial Policy Checklist.

Statistics

For all statistical analyses, confirm that the following items are present in the figure legend, table legend, main text, or Methods section.

n/a	Confirmed	
☐	☒	The exact sample size (*n*) for each experimental group/condition, given as a discrete number and unit of measurement
☐	☒	A statement on whether measurements were taken from distinct samples or whether the same sample was measured repeatedly
☐	☒	The statistical test(s) used AND whether they are one- or two-sided<br>*Only common tests should be described solely by name; describe more complex techniques in the Methods section.*
☐	☒	A description of all covariates tested
☐	☒	A description of any assumptions or corrections, such as tests of normality and adjustment for multiple comparisons
☐	☒	A full description of the statistical parameters including central tendency (e.g. means) or other basic estimates (e.g. regression coefficient) AND variation (e.g. standard deviation) or associated estimates of uncertainty (e.g. confidence intervals)
☐	☒	For null hypothesis testing, the test statistic (e.g. *F*, *t*, *r*) with confidence intervals, effect sizes, degrees of freedom and *P* value noted<br>*Give P values as exact values whenever suitable.*
☒	☐	For Bayesian analysis, information on the choice of priors and Markov chain Monte Carlo settings
☒	☐	For hierarchical and complex designs, identification of the appropriate level for tests and full reporting of outcomes
☒	☐	Estimates of effect sizes (e.g. Cohen's *d*, Pearson's *r*), indicating how they were calculated

Our web collection on statistics for biologists contains articles on many of the points above.

Software and code

Policy information about availability of computer code

Data collection	No code was utilized in processing of data or analysis
Data analysis	No software was used

For manuscripts utilizing custom algorithms or software that are central to the research but not yet described in published literature, software must be made available to editors and reviewers. We strongly encourage code deposition in a community repository (e.g. GitHub). See the Nature Portfolio guidelines for submitting code & software for further information.

Data

Policy information about availability of data

All manuscripts must include a data availability statement. This statement should provide the following information, where applicable:

- Accession codes, unique identifiers, or web links for publicly available datasets
- A description of any restrictions on data availability
- For clinical datasets or third party data, please ensure that the statement adheres to our policy

All data generated or analyzed during this study are included in this published article and its supplementary information files.

Research involving human participants, their data, or biological material

Policy information about studies with human participants or human data. See also policy information about sex, gender (identity/presentation), and sexual orientation and race, ethnicity and racism.

Reporting on sex and gender	Sex was reported based on self-reporting methods.
Reporting on race, ethnicity, or other socially relevant groupings	Race and ethnic group is reported based upon self-reporting methods.
Population characteristics	Participants self reported age at enrollment and relevant identification documented
Recruitment	Open recruitment was made through the Infectious Diseases Clinical Research Consortium network across ten vaccine training and evaluation units within the United States
Ethics oversight	Advarra served as the IRB of record with site-specific IRB reliance agreements.

Note that full information on the approval of the study protocol must also be provided in the manuscript.

Field-specific reporting

Please select the one below that is the best fit for your research. If you are not sure, read the appropriate sections before making your selection.

☒ Life sciences ☐ Behavioural & social sciences ☐ Ecological, evolutionary & environmental sciences

For a reference copy of the document with all sections, see nature.com/documents/nr-reporting-summary-flat.pdf

Life sciences study design

All studies must disclose on these points even when the disclosure is negative.

Sample size	Safety and immunogenicity were covariate variables. With approximately 50 subjects in each group there is a 99.5% chance of observing at least one AE of probability 10%. Similarly, with approximately 25 subjects in each of the age subgroups, there is a 92.8% chance of observing at least one AE of probability 10%. Therefore, if no AEs of a given type occur in a Cohort 1 group, we can be relatively confident that they will occur in fewer than 10% of people once the vaccine is implemented.<br>A co-primary objective of this study is to evaluate the magnitude of SARS-CoV-2 specific antibody titers in serum samples. This objective is descriptive in nature and will be accomplished by estimating 95% confidence intervals (CI) for the geometric mean titer (GMT) at each timepoint when samples are collected.
Data exclusions	The safety analysis population includes all enrolled subjects who received at least one dose of study vaccine. Analyses for the safety population will include safety reported through the end of the study. The modified intent-to-treat (mITT) population includes all subjects who received at least one dose of vaccine and contributed both pre- and at least one post-vaccination venous blood sample for immunogenicity testing for which valid results were reported.<br>In the final analysis, protocol deviations will be reviewed to determine which protocol deviations may affect the analysis. The per protocol (PP) population will then be defined – and this includes all subjects in the mITT subset with the following exclusions:<br>• Data from all available visits for subjects found to be ineligible at baseline.<br>• Data from all visits subsequent to the protocol deviations that are considered to affect the science.<br>• Data from any visit that occurs substantially out of window.
Replication	Samples are processed in duplicate.
Randomization	Subjects in Cohorts 1 and 2 will not be randomized to study intervention. The study was open label, staged and competitive recruitment across all ten sites and study sites will administer product to which they have been assigned.
Blinding	This study is unblinded.

Reporting for specific materials, systems and methods

We require information from authors about some types of materials, experimental systems and methods used in many studies. Here, indicate whether each material, system or method listed is relevant to your study. If you are not sure if a list item applies to your research, read the appropriate section before selecting a response.

Materials & experimental systems

n/a	Involved in the study
☐	☒ Antibodies
☐	☐ Eukaryotic cell lines
☐	☐ Palaeontology and archaeology
☐	☐ Animals and other organisms
☐	☒ Clinical data
☐	☐ Dual use research of concern
☐	☐ Plants

Methods

n/a	Involved in the study
☒	☐ ChIP-seq
☒	☐ Flow cytometry
☒	☐ MRI-based neuroimaging

Antibodies

Antibodies used

Alexaflour-647 Novus Biologicals ab269823
CR3022 Sigma-Aldrich ZHU1077

Validation

Research validation through the laboratory of Dr. David Montefiori - Duke University

Eukaryotic cell lines

Policy information about cell lines and Sex and Gender in Research

Cell line source(s)

HEK 293T/17 cells- American Type Culture Collection-Cat# CRL-11268
293T/ACE2 cell -Drs. Michael Farzan and Huihui Mu- N/A
VeroE6 C1008 cells -ATCC -Cat# CRL-1586, RRID:CVCL_0574

Authentication

Refer to Supp Appendix of citations: 1. Atmar, R.L., Lyke, K.E. et al. Homologous and heterologous Covid-19 booster vaccinations. N Engl J Med 386, 1046-1057 (2022).
2. Lyke, K.E., Atmar R.L., et al. Rapid decline in vaccine-boosted neutralizing antibodies against SARS-CoV-2 Omicron variant. Cell Rep Med 3, 100679 (2022).

Mycoplasma contamination

Not tested.

Commonly misidentified lines
(See ICLAC register)

N/A

Palaeontology and Archaeology

Specimen provenance

N/A

Specimen deposition

N/A

Dating methods

N/A

☐ Tick this box to confirm that the raw and calibrated dates are available in the paper or in Supplementary Information.

Ethics oversight

N/A

Note that full information on the approval of the study protocol must also be provided in the manuscript.

Animals and other research organisms

Policy information about studies involving animals; ARRIVE guidelines recommended for reporting animal research, and Sex and Gender in Research

Laboratory animals

N/A

Wild animals

N/A

Reporting on sex

N/A

Field-collected samples

N/A

Ethics oversight	N/A

Note that full information on the approval of the study protocol must also be provided in the manuscript.

Clinical data

Policy information about clinical studies

All manuscripts should comply with the ICMJE guidelines for publication of clinical research and a completed CONSORT checklist must be included with all submissions.

Clinical trial registration	ClinicalTrials.gov: NCT04889209
Study protocol	Uploaded to NPJVaccines
Data collection	Supplementary Appendix provides the location and investigators for all ten clinical trial sites.
Outcomes	The primary outcome is safety and immunogenicity. Refer to protocol Section 9.4.3.

Dual use research of concern

Policy information about dual use research of concern

Hazards

Could the accidental, deliberate or reckless misuse of agents or technologies generated in the work, or the application of information presented in the manuscript, pose a threat to:

No	Yes	
☒	☐	Public health
☒	☐	National security
☒	☐	Crops and/or livestock
☒	☐	Ecosystems
☒	☐	Any other significant area

Experiments of concern

Does the work involve any of these experiments of concern:

No	Yes	
☒	☐	Demonstrate how to render a vaccine ineffective
☒	☐	Confer resistance to therapeutically useful antibiotics or antiviral agents
☒	☐	Enhance the virulence of a pathogen or render a nonpathogen virulent
☒	☐	Increase transmissibility of a pathogen
☒	☐	Alter the host range of a pathogen
☒	☐	Enable evasion of diagnostic/detection modalities
☒	☐	Enable the weaponization of a biological agent or toxin
☒	☐	Any other potentially harmful combination of experiments and agents
